# Clustering analysis and prognostic signature of lung adenocarcinoma based on the tumor microenvironment

**DOI:** 10.1038/s41598-022-15971-4

**Published:** 2022-07-14

**Authors:** Qingqing Shan, Yifan Zhang, Zongan Liang

**Affiliations:** 1 Department of Respiration, West China Hospital of Sichuan University, 37# Guo Xue Xiang, Chengdu, 610041 Sichuan China; 2 Department of Respiration, Chengdu First People’s Hospital, Chengdu, 610041 China

**Keywords:** Cancer, Computational biology and bioinformatics, Medical research, Oncology

## Abstract

Because of immunotherapy failure in lung adenocarcinoma, we have tried to find new potential biomarkers for differentiating different tumor subtypes and predicting prognosis. We identified two subtypes based on tumor microenvironment-related genes in this study. We used seven methods to analyze the immune cell infiltration between subgroups. Further analysis of tumor mutation load and immune checkpoint expression among different subgroups was performed. The least absolute shrinkage and selection operator Cox regression was applied for further selection. The selected genes were used to construct a prognostic 14-gene signature for LUAD. Next, a survival analysis and time-dependent receiver operating characteristics were performed to verify and evaluate the model. Gene set enrichment analyses and immune analysis in risk groups was also performed. According to the expression of genes related to the tumor microenvironment, lung adenocarcinoma can be divided into cold tumors and hot tumors. The signature we built was able to predict prognosis more accurately than previously known models. The signature-based tumor microenvironment provides further insight into the prediction of lung adenocarcinoma prognosis and may guide individualized treatment.

## Introduction

Lung cancer is the leading global cause of cancer-related death, and lung adenocarcinoma (LUAD) is the most common histological subtype^1^. LUAD is usually diagnosed as disseminated metastatic tumors at an advanced stage, and the 5-year overall survival (OS) rate of LUAD is less than 20%^2^. With the development of molecular targeted drugs and immunotherapy, the prognosis of lung cancer has improved. Nevertheless, drug resistance is inevitable for targeted therapy, and before immunotherapy, medical workers need to screen appropriate patients to improve efficacy. Currently, common screening indicators, including *PD-L1* (*CD274*) expression, tumor mutation burden (TMB), and other molecules, such as *CD28* and lactate dehydrogenase, are also used to assist in the judgment of immunotherapy efficacy^3^.

The tumor microenvironment (TME) composition varies with tumor types, including immune cells, stromal cells, blood vessels, and extracellular matrix (ECM)^4^. It is believed that “TME is not just a silent bystander but rather an active promoter of cancer progression”. In the early stage of tumor growth, there is a dynamic and reciprocal relationship between cancer cells and TME components to support the survival, local invasion, and metastatic spread of cancer cells. The TME coordinates a plan to promote angiogenesis, restore oxygen/nutrition supply, and remove metabolic waste to overcome anoxia and an acidic microenvironment. An increasing number of studies on the TME have identified a new goal of therapeutic intervention^5^.

Currently, targeting specific cells in the TME has become a new therapeutic strategy. Immune checkpoint blocking (ICB) is the first generation of antibody-based therapy for immune cells in the TME. ICB inhibits the activation and function of T cells by blocking receptor/ligand interactions (such as *CTLA4* and *PD-1*). Patients who respond to ICB have significant clinical benefits, but most patients do not respond to ICB^6^. Some of the reasons may be related to the immune microenvironment of the tumor. Among them, tumor-infiltrating lymphocyte (TIL) status is the most critical issue. Only in the presence of infiltrating lymphocytes can the antigen show immunogenicity^7^. According to TILs, some studies have classified tumors into so-called “cold tumors” and “hot tumors”. In short, hot tumors are tumors infiltrated by lymphocytes, while cold tumors are the opposite. The effective rate of PD-1/PD-L1 inhibitors in cold tumors may be lower, while tumors with high expression of PD-L1 in hot tumors are more likely to respond to PD-1/PD-L1 treatment^8^.

Therefore, scholars have developed many methods to study the tumor immune environment, immune cells, stromal cells, etc. Many signatures related to the TME have been designed to evaluate the prognosis and immune status of LUAD patients. However, the use of signatures associated with TME to assess the immune situation of patients is very limited and not comprehensive enough^9–11^.

This study classified LUAD patients according to TME-related genes, which helped us distinguish between cold and hot tumors. Furthermore, we studied the prognosis, immune status, and mutation of patients with different tumor types. At the same time, we also established a signature related to the TME to help assess the patient’s prognosis and immune status. We found that our signature is superior to other signatures in evaluating the prognosis of patients.

## Materials and methods

Data supporting the results and conclusion of this article can be downloaded from the online version of the dashboard freely. And all methods were performed in accordance with the relevant guidelines and regulations.

### Data collection

Gene expression profiles, somatic mutation profiles, and clinical data of 497 LUAD tissues and 54 adjacent normal tissues were obtained from the cancer genome atlas (TCGA) database (https://portal.gdc.cancer.gov/). Duplicate samples were combined, and the gene expression of the same samples was taken as their average value, so 20 samples were deleted. Furthermore, nine cases were not included due to the lack of survival materials. Finally, 468 patients with LUAD were eventually enrolled in further study. Clinical data of 468 patients, including survival data, are provided in Table S1. Moreover, GSE42127 was downloaded from the gene expression omnibus (GEO) database (https://www.ncbi.nlm.nih.gov/geo). We removed samples without clinical information and only retained LUAD samples. After this process, 133 LUAD samples from the GEO cohort remained.

A total of 4061 TME-related genes were obtained from published research after sorting^9,11–13^ (Table S2).

### NMF analysis based on TME-related genes

Non-negative matrix factorization (NMF) is an algorithm based on partial factorization that reduces the expression data of thousands of genes to a few meta-genes. NMF is an effective method to identify different molecular patterns and provides a powerful class discovery method. Research shows that a classification based on NMF is more accurate and robust for clustering genome data compared with other methods^14^. Before clustering, we analyzed the differentially expressed genes, and 993 differentially expressed genes (*p* < 0.05) were retained for sample clustering analysis. Then, unsupervised NMF clustering was conducted via the package “NMF” in R language on the TCGA datasets. The k value when the cophenetic correlation coefficient started to decline was chosen as the optimal number of clusters. The number of clusters k ranged from 2 to 10. When k = 2, the cluster demonstrated proper stability and performance, resulting in clusters 1 and 2. The selection was based on cophenetic and residual sum of squares (rss).

### Investigation of immune states

At present, seven commonly used methods for studying tumor immune infiltration include TIMER^15^, CIBERSORT^16^, XCELL^17^, QUANTISEQ^18^, MCPcounter (https://github.com/ebecht/MCPcounter), EPIC^19^, and Cibersort on Timer 2.0 (http://timer.cistrome.org/). Immune cell infiltration analysis was performed on the samples using the seven methods described above. “GSVA” and “GSEABase” are R’s open-source software packages, which are part of the Bioconductor project and can be downloaded at http://www.bioconductor.org^20^. The “GSVA”, “GSEABase”, and “limma” R packages were used to analyze the immune cells and immune function of the samples. We also compared TME scores and immune checkpoint activation between the HRG and LRG through the “estimate”, “ggpubr”, and “limma” R packages.

### Establishment of the TME-related gene signature

Univariate Cox proportional hazards regression analysis was used to screen for genes significantly associated with prognosis (*p* < 0.05)^21^. We further used Lasso Cox regression to reduce the number of genes in the risk model. After performing 1000 tenfold cross-validations, we selected the optimum λ parameter value in which the error was minimized^22^. Multivariate Cox proportional hazards regression analysis was used to identify key genes involved in establishing a predictive model^23,24^ (Supplementary material). Based on the expression of these genes, we established a risk formula for TME-related genes.

According to the risk formula score, the risk score (RS) for each patient was calculated, and patients were categorized into the high-risk group (HRG) or low-risk group (LRG) compared to the median value. Survival curves were plotted to evaluate the prediction effect of the model. The predictive performance of this model at different endpoints (1, 3, or 5 years) was assessed using a time-dependent receiver operating characteristic (ROC) curve^25^.

### Validation of the TME-related gene signature

ANOVA was used for parametric data to compare more than two groups, and Kruskal–Wallis tests were used for nonparametric data. We used Kaplan–Meier method analyzes OS and progression-free survival (PFS). The “p-ROC” package was used to analyze the prognostic classification effect of the RS. We used the concordance index (C-index) to compare the prediction performance between different models. The C-index is the fraction of all pairs of individuals whose predicted survival times are correctly ordered^26^ and is based on Harrell’s C statistics^27^. A C-index score of approximately 0.70 indicates a good model, whereas a score of approximately 0.50 indicates a random background. The calculation method of the C-index can be found in the supplementary material.

### Statistical analysis

R (https://www.rproject.org/) is a free software environment for statistical computing and graphics. Strawberry Perl (https://www.perl.org) is a perl environment for MS Windows containing all you need to run and develop perl applications. We used Strawberry Perl 5.30.0 and R v4.1.1 to conduct data conversion, statistical analysis, and calculations. P values less than 0.05 on both sides were considered statistically significant.

## Results

### NMF clustering analysis based on TME-associated gene expression

**Figure 1:**
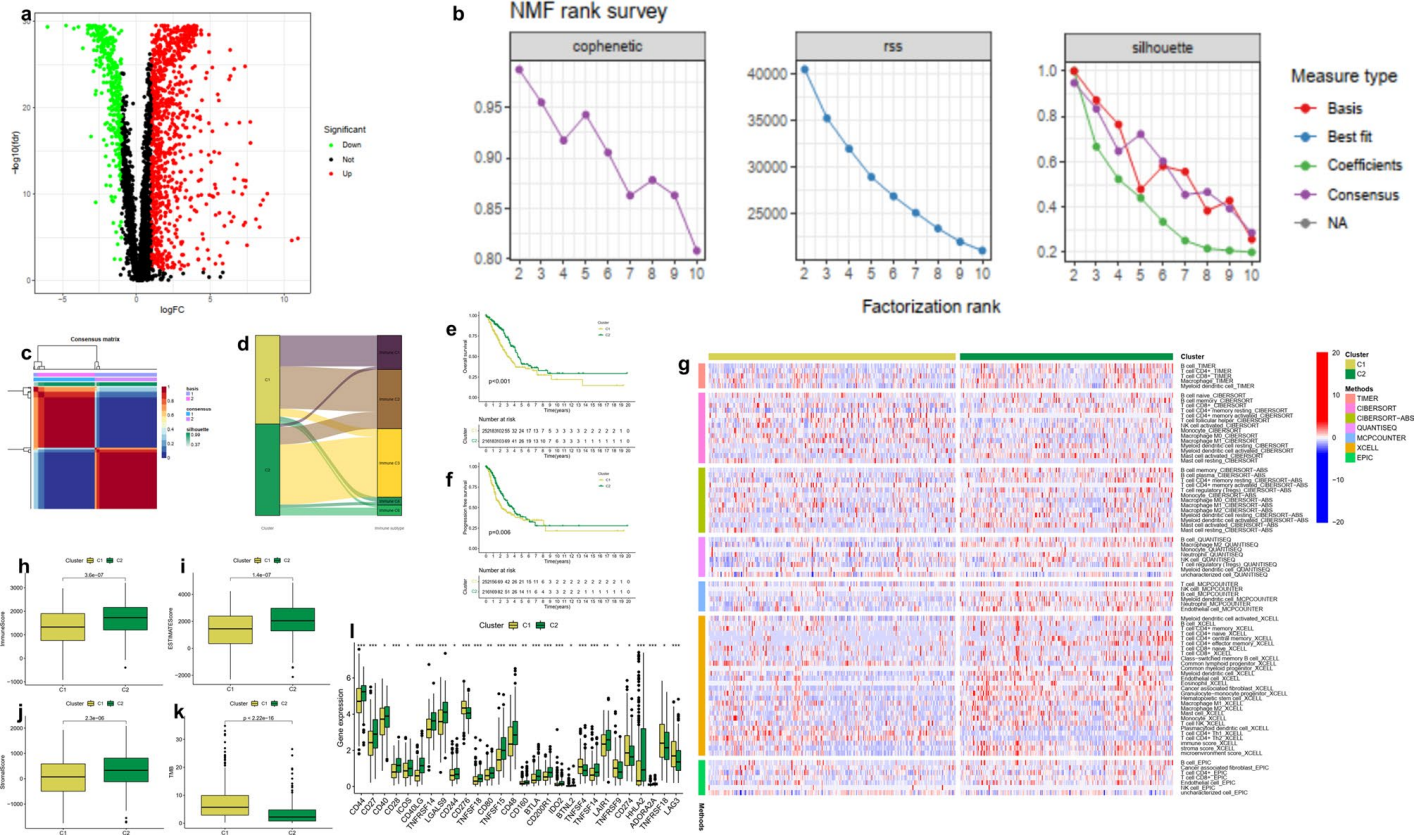
Two subgroups of LUAD were identified based on TME. (**a**) Identification of differentially TME-related expressed genes in the LUAD and normal groups. (**b**) The cophenetic coefficient, residual sum of squares, and silhouette in NMF analysis. (**c**) The consensus map of NMF analysis. (**d**) The Sankey map of molecular subtypes compared with existing molecular immune subtypes. (**e**) Analysis of the OS of C1 and C2. (**f**) PFS of C1 and C2. (**g**) Immune cell infiltration in the two clusters. (**h**) Immune cell scores in the two clusters. (**i**) Stromal cell scores in the two clusters. (**j**) ESTIMATE scores in the two clusters. (**k**) TMB in the two clusters. (**l**) The differences in the expression of 28 immune checkpoint molecules in the two clusters.

The characteristics of the 468 TCGA-LUAD samples and 133 GSE42127 samples selected for analysis are shown in Table 1. In the TCGA database, 993 differentially expressed genes (Table S3) were obtained by differential analysis. A volcano plot was presented to summarize the expression levels of TME-related genes in normal and LUAD patients in the TCGA database (Fig. 1a). According to indicators, the 993 genes were used to determine the optimal number of clusters, such as the cophenetic coefficient, rss, and silhouette. The consensus summary statistics analysis indicated that the optimal number of gene clusters was 2. Then, TCGA-LUAD samples were divided into two clusters: cluster 1 and 2 (Fig. 1b,c). Survival analysis showed that OS and PFS in cluster 2 were better than in cluster 1 (Fig. 1e,f).

**Table 1:** Characteristics of the TCGA-LUAD and GSE42127 cohorts.

Clinical features	TCGA-LUAD (468)	GSE42127 (133)
**Fustat**		
0 (alive)	291	90
1 (dead)	177	43
**T stage**		
T1	159	
T2	248	
T3	39	
T4	19	
TX	3	
**N stage**		
N0	302	
N1	86	
N2	66	
N3	2	
NX and unknown	12	
**M stage**		
M0	315	
M1	24	
MX and unknown	129	
**Stage**		
I	253	89
II	107	22
III	75	20
IV	25	1
Unknown	8	1
**Gender**		
Male	214	68
Female	254	65
**Age**		
≤ 65	224	63
> 65	234	70
Unknown	10

According to the TCGA database, LUAD can be divided into five categories. Each immune subtype represents a specific immune microenvironment, namely, immune C1 (wound healing), immune cluster 2 (IFN-gamma dominant), immune C3 (inflammatory), immune C4 (lymphocyte depleted), and immune C6 (TGF-beta dominant)^28^. A Sankey diagram showed that most of the immune C1 and immune C2 belong to cluster 1 with a poor prognosis, while most of the immune C3 and immune C6 belong to cluster 2 with a good prognosis (Fig. 1d, Table 2).

**Table 2:** The proportion of different immune subtypes of TCGA in clusters 1 and 2.

**Cluster 1 (49.27%)**	
Immune C1 (wound healing)	34.98%
Immune C2 (IFN-gamma dominant)	47.78%
Immune C3 (inflammatory)	8.87%
Immune C4 (lymphocyte depleted)	5.42%
Immune C6 (TGF-beta dominant)	2.96%
**Cluster 2 (50.73%)**	
Immune C1 (wound healing)	3.35%
Immune C2 (IFN-gamma dominant)	18.66%
Immune C3 (inflammatory)	66.03%
Immune C4 (lymphocyte depleted)	3.35%
Immune C6 (TGF-beta dominant)	8.61%

Multiple platforms analyses showed that cluster 1 had a lower degree of immune cell infiltration (Fig. 1g). Cluster 1 had more deficient immune cells and stromal cells, and higher ESTIMATE scores (Fig. 1h–j), suggesting that cluster 2 had a different TME from cluster 1. Almost all immune checkpoints expressed lower activity in cluster 1, such as *CD28*, *LAG3*, and *IDO2* (Fig. 1l). Interestingly, *CD274* (*PD-L1*) expression in cluster 2 was low. TMB analysis showed that TMB was lower in the cluster 2 group with better prognoses (Fig. 1k).

### Establishment of a signature-based on TME-related genes

The training cohort was composed of 328 samples, and the testing cohort comprised 140 samples. We established a prognostic signature in the training cohort and verified this signature in the testing cohort and GEO cohort. The results of the chi-squared test showed no significant differences in age, sex, or stage between them (p > 0.05), which confirmed the success of randomization (Table 3). Uni-Cox regression analysis of the training cohort identified 326 prognostic TME-related genes within the threshold of *p* < 0.05 (Table S4). We used the “glmnet” package to narrow the gene range further while maintaining high accuracy. Finally, an RS model containing 14 genes was screened by the Lasso machine learning method, constructing the formula RS = + (0.121264434 × *SOX9*) + (0.34450123 × *DHFR*) + (0.28495724 × *PLEK2*) + (0.094359082 × *BARX1*) + (0.287363067 × *PAQR5*) + (0.33495707 × *PAQR4*) + (0.301608897 × *SEC61G*) + (0.634478861 × *CHD1 L*) + (0.311633832 × *CDH2*) + (0.727973014 × *NAALADL2*) + (0.226921565 × *MIF*) − (0.247563521 × *CAPN13*) − (0.440345818 × *CTLA4*) − (0.323595106 × *TM6SF1*). In the HPA database (https://www.proteinatlas.org/), we analyzed the expression of these genes in normal tissues and lung cancer tissues, and the results are shown in the supplementary materials. According to the median RS value, the samples were subsequently divided into a high-risk group (HRG) or a low-risk group (LRG). The relationship between risk groups and clusters is shown in Table 4 and Fig. 3h.

**Table 3:** Comparison of TCGA training and testing cohorts.

Covariates	Type	Total	Test	Train	*p*-value
Age	≤ 65	224 (47.86%)	70 (50%)	154 (46.95%)	0.4763
	> 65	234 (50%)	65 (46.43%)	169 (51.52%)	
	Unknown	10 (2.14%)	5 (3.57%)	5 (1.52%)	
Gender	Female	254 (54.27%)	68 (48.57%)	186 (56.71%)	0.1294
	Male	214 (45.73%)	72 (51.43%)	142 (43.29%)	
Stage	Stage I	253 (54.06%)	80 (57.14%)	173 (52.74%)	0.4825
	Stage II	107 (22.86%)	30 (21.43%)	77 (23.48%)	
	Stage III	75 (16.03%)	19 (13.57%)	56 (17.07%)	
	Stage IV	25 (5.34%)	10 (7.14%)	15 (4.57%)	
	Unknown	8 (1.71%)	1 (0.71%)	7 (2.13%)	
T	T1	159 (33.97%)	45 (32.14%)	114 (34.76%)	0.5161
	T2	248 (52.99%)	75 (53.57%)	173 (52.74%)	
	T3	39 (8.33%)	15 (10.71%)	24 (7.32%)	
	T4	19 (4.06%)	4 (2.86%)	15 (4.57%)	
	TX	3 (0.64%)	1 (0.71%)	2 (0.61%)	
M	M0	315 (67.31%)	88 (62.86%)	227 (69.21%)	0.4443
	M1	24 (5.13%)	9 (6.43%)	15 (4.57%)	
	MX and unknown	129 (27.56%)	43 (30.71%)	86 (26.22%)	
N	N0	302 (64.53%)	95 (67.86%)	207 (63.11%)	0.5802
	N1	86 (18.38%)	22 (15.71%)	64 (19.51%)	
	N2	66 (14.1%)	20 (14.29%)	46 (14.02%)	
	N3	2 (0.43%)	0 (0%)	2 (0.61%)	
	NX and unknown	12 (2.56%)	3 (2.14%)	9 (2.74%)

**Table 4:** The relationship between risk groups and clusters.

**HRG (233)**	
Cluster 1 (174)	37.18%
Cluster 2 (59)	12.61%
**LRG (235)**	
Cluster 1 (78)	16.67%
Cluster 2 (157)	33.55%

**Figure 2:**
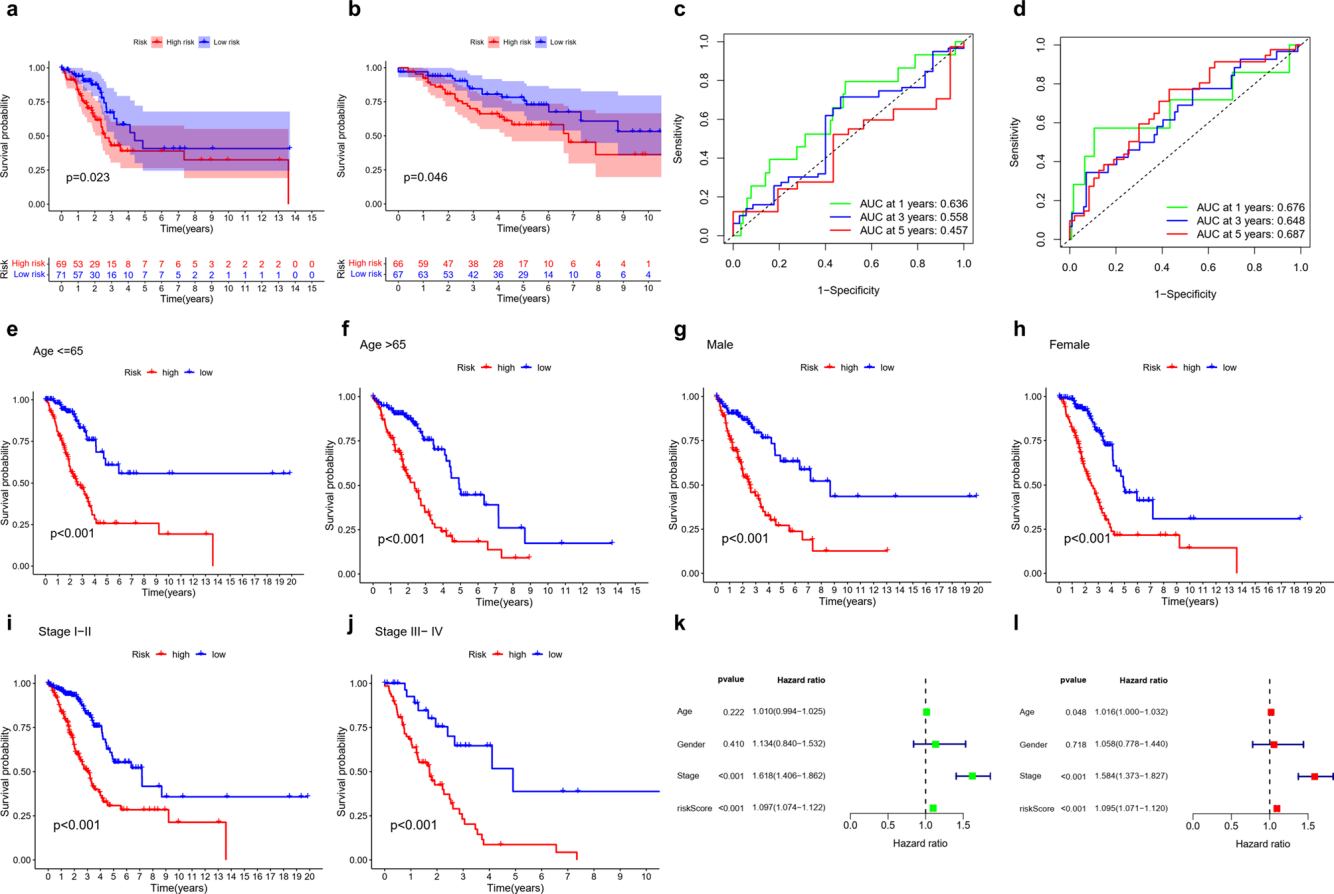
Establishment and verification of the TME-related gene signature. Survival curve of the testing cohort (**a**) and GEO cohort (**b**). ROC curves for forecasting OS in the testing cohort (**c**) and GEO cohort (**d**). Evaluation of the 14-gene risk signature in different ages, sexes, and stages comparing the LRG and the HRG. (**e**) Patients aged ≤ 65 years. (**f**) Patients aged > 65 years. (**g**) Male patients. (**h**) Female patients. (**i**) Patients at stages I–II. (**j**) Patients at stages III–IV. (**k**) Uni-Cox regression analysis of clinical characteristics and RS. (**l**) Multi-Cox regression analysis of clinical characteristics and RS.

Survival analysis showed that the prognosis of the HRG was significantly poorer than that of the LRG in the TCGA testing cohort and GEO cohort (Fig. 2a,b). We also assessed the predictive value of the 14-gene risk model using time-dependent ROC analysis. The 1-year AUCs of the entire TCGA and GEO cohorts were 0.636 and 0.676, respectively (Fig. 2c,d).

Furthermore, we categorized patient subgroups by age (≤ 65 years and > 65 years), sex, and stage (I-II and III-IV). In addition, we divided the samples into the HRG and LRG based on the already mentioned RS. We found a significant difference in prognosis between the HRG and the LRG (Fig. 2e–j). Our data indicate that our prediction model can predict prognosis in patients of different ages, sexes, and stages. To investigate whether the signature was an independent prognostic indicator, uni- and multi-Cox regression analyses were performed. Uni-Cox analysis revealed that the HR and 95% CI for RS were 1.097 and 1.074–1.112, respectively (p < 0.001) (Fig. 2k), while multi-Cox analysis revealed that the HR and 95% CI for RS were 1.095 and 1.071–1.120, respectively (p < 0.001) (Fig. 2l).

### Verification of the signature-based on TME-related genes

**Figure 3:**
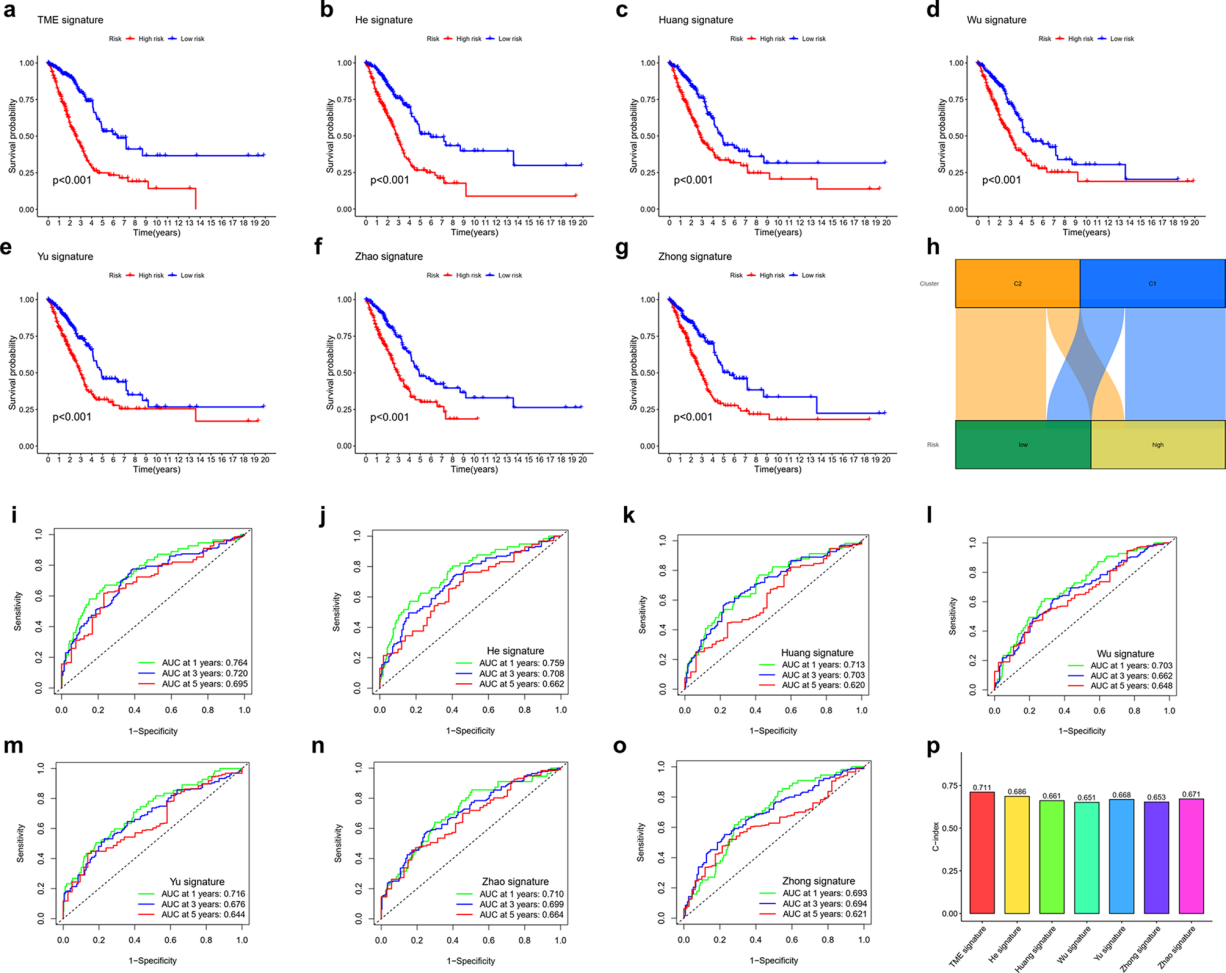
Comparing the TME signature with other models. Survival analysis of seven signatures (**a**–**g**). (**h**) The Sankey map of the two clusters compared with two groups. ROC curves of six signatures (**i**–**o**). (**p**) C-index of the seven prognostic risk signatures.

To compare the prediction performance of our 14-gene signature with other models, we selected six other reported risk models: He’s 37-gene^29^, Huang’s 3-gene^10^, Wu’s 8-gene^9^, Yu’s 12-gene^30^, Zhao’s 19-gene^31^ and Zhong’s 9-gene signatures^32^. To validate these results, we calculated the RS and evaluated the ROC of each dataset using the same method to make them comparable. The prognosis for the LRG and HRG was significant in all seven models (Fig. 3a–g). However, the ROC curves showed a lower AUC in the other six signatures. Therefore, they were poorer in predicting prognoses than our model (Fig. 3i–o). We calculated the C-index of all prognostic signatures, and the results showed that our model had the highest C-index of 0.711 (Fig. 3p).

**Figure 4:**
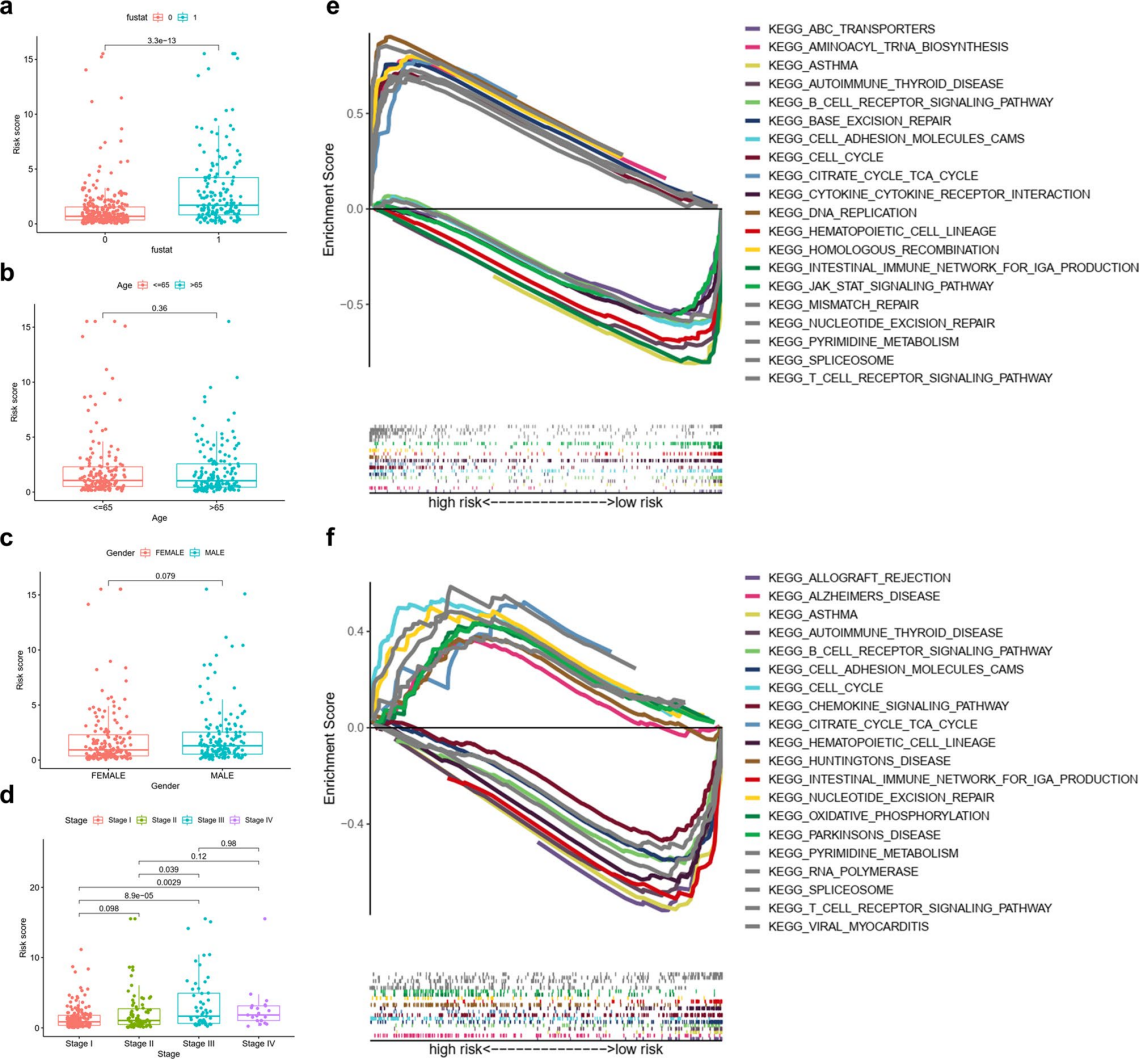
Correlation analysis between RS and survival status (**a**) (“fustat” mean “survival state”, 0 = alive, 1 = dead), age (**b**), gender (**c**), and stage (**d**) in the TCGA cohort. GSEA analysis and immune characteristics in the HRG and LRG. (**e**) The TCGA-LUAD cohort. (**f**) The GEO-GSE42127 cohort.

Furthermore, we categorized patient subgroups by survival status (dead or alive), age (≤ 65 or > 65 years), sex (male or female), and stage (I, II, III, or IV), and then we calculated RS for each sample. Our results showed that alive patients and stage I displayed a low RS (p < 0.05), while there was no difference in RS among different ages, sex, and later stages in LUAD (stage II, III, and IV) (Fig. 4a–d).

### GSEA and immune state analysis

Next, we used GSEA to evaluate the activity of pathways in TCGA-LUAD and GEO-GSE42127 datasets. We found that the action of immune-related pathways in the LRG was significantly higher than that in the HRG (Fig. 4e,f). Both in TCGA-LUAD datasets and GEO-GSE42127, immune-related pathways included several processes: asthma, intestinal immune network for IgA production, B cell receptor signaling pathways, and T cell receptor signaling pathways. Therefore, we attempted to perform an immunoassay in TCGA-LUAD datasets.

**Figure 5:**
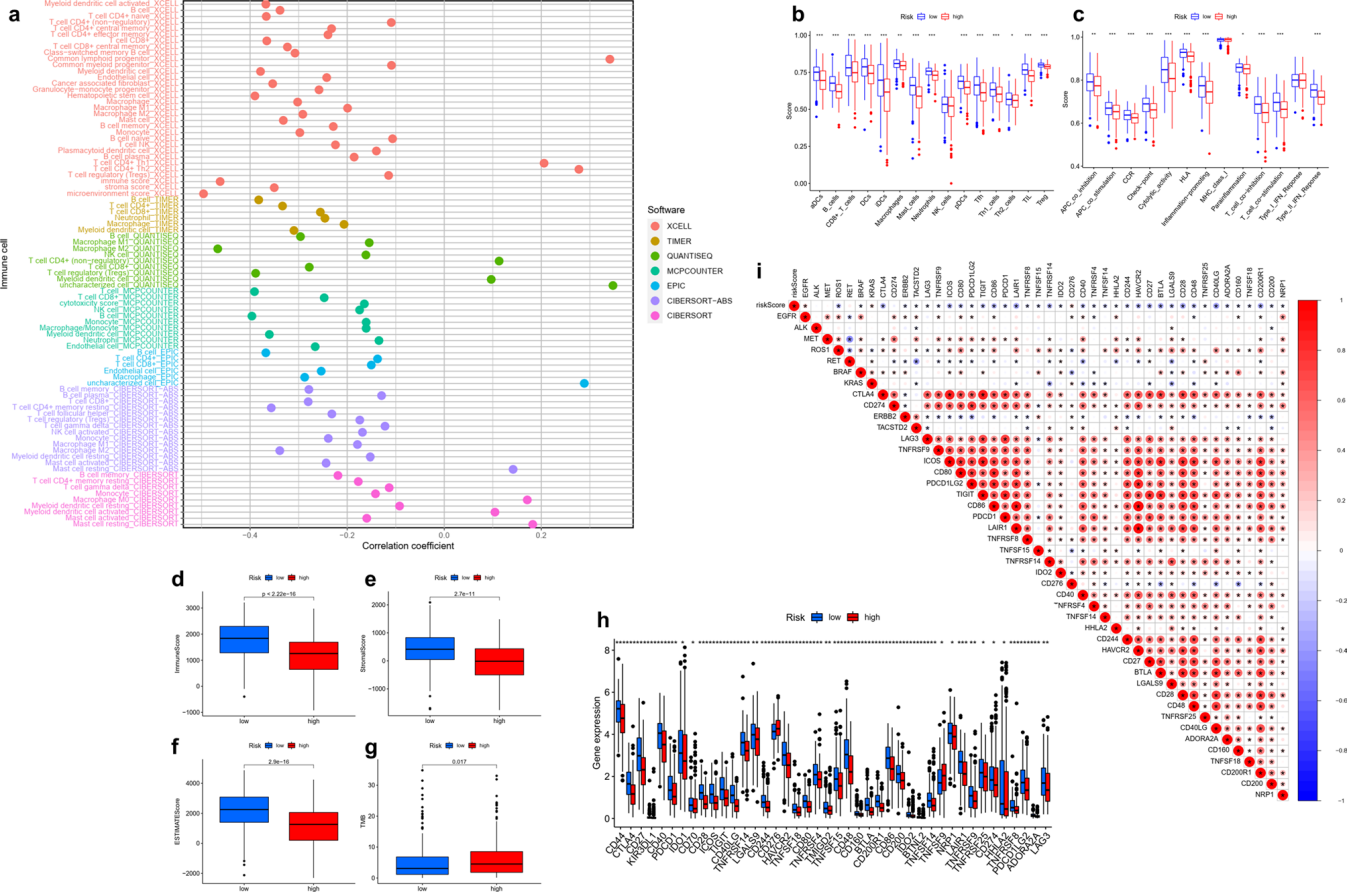
Immune characteristics in the HRG and LRG. (**a**) The bubble chart shows the relationship between RS and immune cells. The ssGSEA analysis for immune cell infiltration (**b**) and immune function (**c**) in the HRG and LRG. (**d**) Immune cell scores in the HRG and LRG. (**e**) Stromal cell scores in the HRG and LRG. (**f**) ESTIMATE scores in the HRG and LRG. (**g**) TMB of the HRG and LRG. (**h**) The difference in the expression of immune checkpoint molecules in the HRG and LRG. (**i**) Correlation between RS and the expression of representative genes for LUAD.

Immune cell bubble plots suggested higher immune cell infiltration in the LRG (Fig. 5a, Table S5). Single-sample gene set enrichment analysis (ssGSEA) was used for immune infiltration analysis of the expression profile. Most immune cells (Fig. 5b) and immune function (Fig. 5c) were increased in the LRG. The ESTIMATE analysis showed that the LRG had higher immune, stromal, and ESTIMATE scores (Fig. 5d–f). TMB analysis showed that TMB was lower in the LRG group with a better prognosis (Fig. 5g). Many immune checkpoints expressed lower activity in the HRG, such as *CD28*, *LAG3*, *CD274* (*PD-L1*), and *IDO2* (Fig. 5h).

Along with the pathway analysis, we extracted the single gene expression of common mutant and checkpoint genes in LUAD. We then analyzed the correlation between the RS and these genes. RS was negatively correlated with *BRAF*, *CTLA4*, *ERBB2, and ROS1*. In contrast, they were positively correlated with *EGFR* and *KRAS* (Fig. 5i). There was no significant correlation between RS and *CD274*.

## Discussion

The disadvantage of immunotherapy is that the treatment may not be practical, depending on the heterogeneity of tumor cells and the tumor microenvironment. At the same time, research shows that TMB is an important marker to predict the therapeutic effect of PD-1/PD-L1 inhibitors. However, the objective remission rate of patients with high TMB is only approximately 30%. TMB combined with PD-L1 indicates that the therapeutic effect of PD-1/PD-L1 inhibitors is not perfect^33^. Therefore, the classification of tumors is of great significance to guide the treatment of cancer patients.

Previous studies have shown that the distinction between cold tumors and hot tumors could guide the immunotherapy of cancer patients^34^. According to the expression of genes related to the tumor microenvironment, we divide LUAD into two molecular subtypes. There are apparent differences between the two molecular subtypes in terms of tumor prognosis, immune cell infiltration, and immune checkpoint gene expression. Therefore, we define cluster 1 as cold tumors and cluster 2 as hot tumors. The study found that immunocyte infiltration, immune score, and most immune checkpoints were higher in cluster 2 than in cluster 1, suggesting that cluster 2 may benefit from immunotherapy. At the same time, the mutation load in cluster 2 was lower than that in cluster 1. All of these may reason for the excellent prognosis of cluster 2.

Interestingly, according to the NMF classification, PD-L1 expression was lower in cluster 2, which had a good prognosis, than in cluster 1. PD-L1 expression was higher in the LRG, which had a better prognosis than the HRG. This shows that PD-L1 alone cannot be used as an indicator to guide immunotherapy. The guiding role of this immune checkpoint molecule is not good, but it may be closely related to the TME, consistent with previous reports. It has been reported in the literature that *PD-L1* predicts a good prognosis in non-small-cell lung cancer^35^, while some reports show that *PD-L1* predicts a poor prognosis^36^. Therefore, *PD-L1* did not predict survival.

Current research indicates that targeted molecular and chemotherapy drugs can affect the tumor microenvironment^37,38^. Therefore, while studying the effects of drugs on tumor cells, it is also essential to study the effects of drugs on the tumor microenvironment. The research and development of drugs to improve the TME has also become a hot topic in the development of tumor drugs. For example, an in situ vaccine could transform an immunosuppressed TME into an immunostimulatory TME that allows effector T cells to enter the tumor bed and kill tumor cells. In addition, cold tumors may be changed into hot tumors by vehicle virus transfection or physical therapy, thereby enhancing the effect of immunotherapy.

We established a signature based on TME-related genes in LUAD that may predict the prognosis of LUAD and applied it to different ages, sexes, and stages. Compared with other signatures reported in the previous literature, the signature we established had the best ROS and C-index values.

We found that immune infiltration and the immune score in the LRG were higher than those in the HRG. This suggests that the infiltration of active immune cells is related to a good prognosis, which is contradictory to some other tumors; for example, gastric cancer with high immune cell infiltration has a poor prognosis^39^. Our unpublished studies found that high immune infiltration of lung squamous cell carcinoma was also associated with poor prognosis. This also suggests the high heterogeneity of tumors and the importance of personalized treatment^4^.

Unlike lung squamous cell carcinoma, many gene mutations can be detected in LUAD, such as *EGFR*, *KRAS*, *BRAF*, *ALK*, and *ROS1*. In our study, *EGFR* and *KRAS* mutations were positively correlated with RS. There are different reports about the influence of *EGFR* on the prognosis of patients. *EGFR* and *KRAS* mutations suggest a poor prognosis in adenocarcinoma patients^40^. It has also been found that there is no difference in the survival rate between patients with an *EGFR* mutation and those without an *EGFR* mutation among patients with LUAD after resection^41^. In the TCGA-LUAC database, 68 patients had *EGFR* mutations (68/497, 13.7%). Of these, 156 patients had treatment data, and only 10 had received targeted therapy (10/156, 6.4%). Therefore, in our study, the positive correlation between RS and *EGFR* may be related to insufficient targeted drug therapy. Studies have shown that *ROS1* rearrangement is a predictive marker of the crizotinib treatment and one of the best prognostic molecular markers in NSCLC^42^. At the same time, the immune checkpoint *CTLA4* was negatively correlated with RS. It has been reported in the literature that the mortality rate is low in patients with *CTLA4*-overexpressing tumors, which is consistent with our research^43^.

## Conclusions

In conclusion, LUAD patients could be divided into two subgroups according to TME-related genes. Their immune status is different and may guide patients to personalized treatment. Our study proposes a TME-related signature that could be implemented in assessing LUAD patients and might improve prognostic accuracy.

## Supplementary Information

Supplementary Information.

Supplementary Tables.

## Data Availability

All data generated or analyzed during this study are included in this published article and its supplementary information files.

## Abbreviations

*ALK*: Anaplastic lymphoma receptor tyrosine kinase
AUC: Area under the ROC curve
*BARX1*: Homeobox protein BarH-like 1
*BRAF*: Serine/threonine-protein kinase B-Raf
*CAPN13*: Calcium-activated neutral proteinase 13
*CD274*: Programmed cell death 1 ligand 1
*CD28*: CD28 antigen
*CDH2*: Cadherin 2
*CHD1 L*: Chromodomain-helicase-DNA-binding protein 1-like
CIBERSORT: Tumor immune estimation resource
C-index: Concordance index
*CTLA4*: Cytotoxic T-lymphocyte protein 4
*DDR2*: Discoidin domain-containing receptor tyrosine kinase 2
*DHFR*: Dihydrofolate reductase
*ECM*: Extracellular matrix
*EGFR*: Epidermal growth factor receptor
*EML4*: Echinoderm microtubule associated protein like 4
*EPIC*: Estimating the proportions of immune and cancer cells
*ERBB2*: Erb-B2 receptor tyrosine kinase 2
*FGFR1*: Fibroblast growth factor receptor 1
GEO: Group on earth observations
GSEA: Gene set enrichment analysis
HRG: High-risk group
ICB: Immune checkpoint blocking
*IDO2*: Indoleamine 2,3-dioxygenase 2
KM: Kaplan–Meier
*KRAS*: Kirsten rat sarcoma viral oncogene homologue
*LAG3*: Lymphocyte activation gene 3 protein
LRG: Low-risk group
LUAD: Lung adenocarcinoma
MCP: Microenvironment cell populations
MIF: Macrophage migration inhibitory factor
*NAALADL2*: *N*-Acetylated alpha-linked acidic dipeptidase like 2
NMF: Non-negative matrix factorization
*NTRK1*: Neurotrophic receptor tyrosine kinase 1
OS: Overall survival
*PAQR4*: Progestin and adipoQ receptor family member IV
*PAQR5*: Progestin and adipoQ receptor family member V
*PLEK2*: Pleckstrin 2
RET: Ret proto-oncogene
RMS: Restricted mean survival
ROC: Receiver operating characteristic
rss: Residual sum of squares
*SEC61G*: Protein transport protein Sec61 subunit gamma
*SOX9*: SRY-box transcription factor 9
*TACSTD2*: Tumor-associated calcium signal transducer 2
TCGA: The Cancer Genome Atlas
TIL: Tumor-infiltrating lymphocyte
TIMER: Tumor immune estimation resource
*TM6SF1*: Transmembrane 6 superfamily member 1
TMB: Tumor mutation burden
TME: Tumor microenvironment
