# Supplementary material for: Clustering analysis and prognostic signature of lung adenocarcinoma based on the tumor microenvironment
Source: Sci Rep. 2022 Jul 14;12:12059. doi: 10.1038/s41598-022-15971-4 (PMC9283441; doi:10.1038/s41598-022-15971-4)
Supplement: Supplementary file 1 — Supplementary Information. [file 41598_2022_15971_MOESM1_ESM.pdf]

Supplementary material

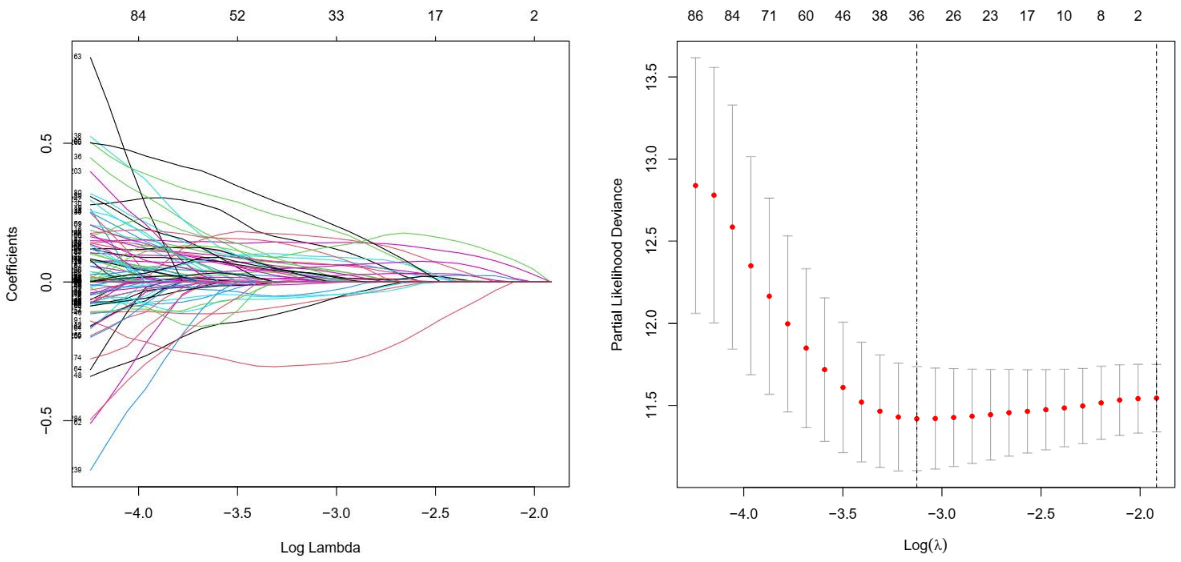

Figure S1. Establishment of a TME-related gene signature. (a) The coefficients of variables were identified based on the Lasso Cox regression model. (b) 10-fold Cross-validation of Lasso regression.

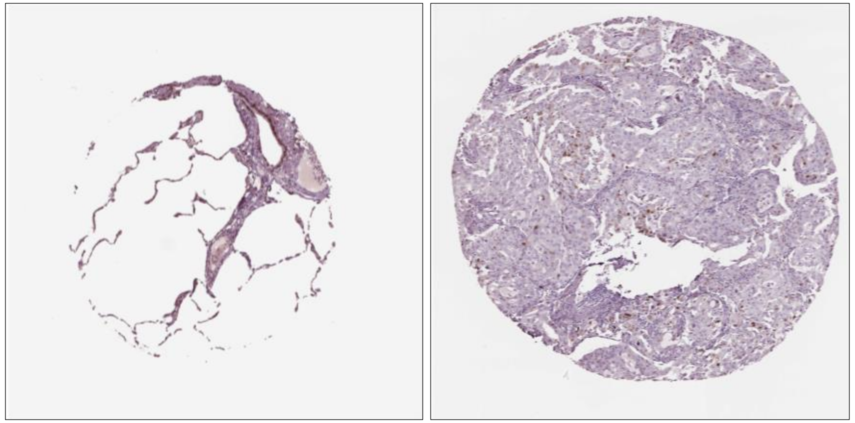

Normal LUAD

Figure S2. Immunohistochemistry analysis of *SOX9* in the HPA database.

SOX9		
Tissue	Normal	LUAD
Antibody	CAB068240	
Patient ID	1678	2003
Age	57	61
Gender	Female	Female
N	Alveolar cells type I	
	Staining	Medium
	Intensity	Strong
	Quantity	<25%
	Alveolar cells type II	
	Staining	Medium
	Intensity	Strong
	Quantity	<25%
	Endothelial cells	
	Staining	Not detected
	Intensity	Negative
	Quantity	None
	Macrophages	
	Staining	Low
	Intensity	Weak
	Quantity	75%-25%
LUAD	Tumor cells	
	Staining	Medium
	Intensity	Strong
	Quantity	<25%
	Location	Nuclear

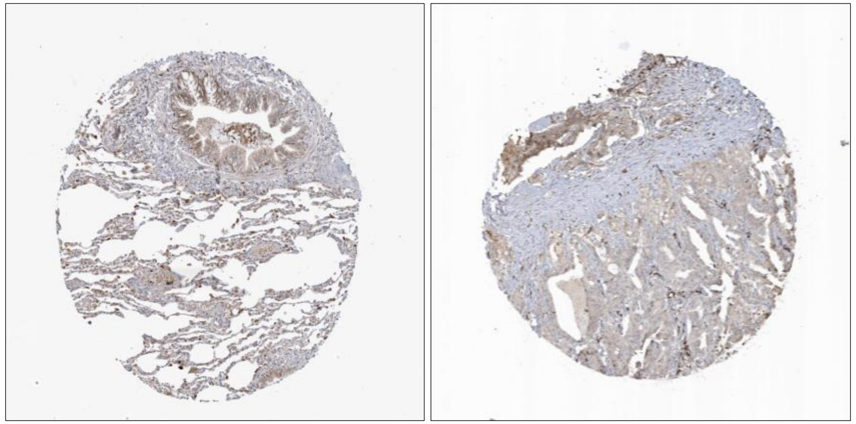

Normal LUAD

Figure S3. Immunohistochemistry analysis of *DHFR* in the HPA database.

DHFR		
Tissue	Normal	LUAD
Antibody	CAB037129	
Patient ID	2268	3052
Age	49	51
Gender	Female	Female
N	Alveolar cells	
	Staining	Low
	Intensity	Moderate
	Quantity	<25%
	Location	Cytoplasmic/membranous
	Macrophages	
	Staining	High
	Intensity	Strong
	Quantity	75%-25%
	Location	Cytoplasmic/membranous
LUAD	Tumor cells	
	Staining	Not detected
	Intensity	Weak
	Quantity	<25%
	Location	Cytoplasmic/membranous

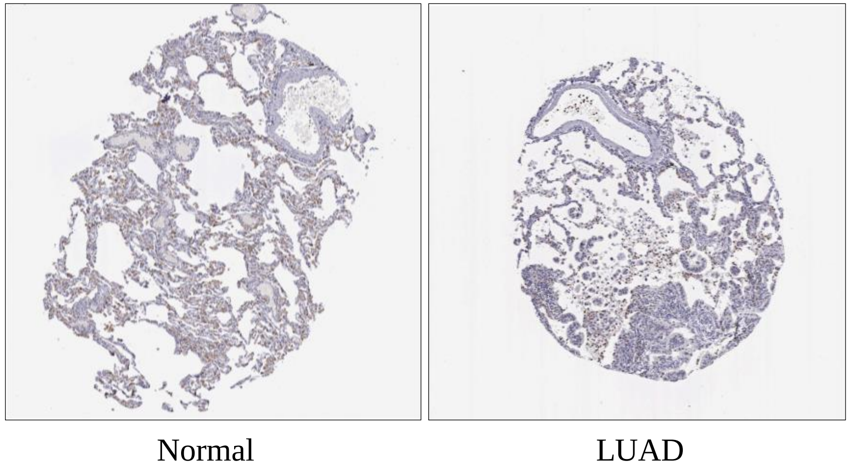

Figure S4. Immunohistochemistry analysis of *PLEK2* in the HPA database.

PLEK2		
Tissue	Normal	LUAD
Antibody	HPA001208	
Patient ID	2268	537
Age	49	50
Gender	Female	Female
N	Alveolar cells	
	Staining	Not detected
	Intensity	Negative
	Quantity	None
	Macrophages	
	Staining	High
	Intensity	Strong
	Quantity	>75%
	Location	Cytoplasmic/membranous
LUAD	Tumor cells	
	Staining	Not detected
	Intensity	Negative
	Quantity	None

	BARX1	
Tissue	Normal	LUAD
	The protein is mainly expressed in gastrointestinal tract	No data

	PAQR5	
Tissue	Normal	LUAD
	Protein expression could not be detected in lung tissue.	No data

	PAQR4	
Tissue	Normal	LUAD
	Protein expression could not be detected in lung tissue.	No data

Figure S5. Immunohistochemistry analysis of *BARX1*, *PAQR5*, and *PAQR4* in the HPA database.

Supplementary material

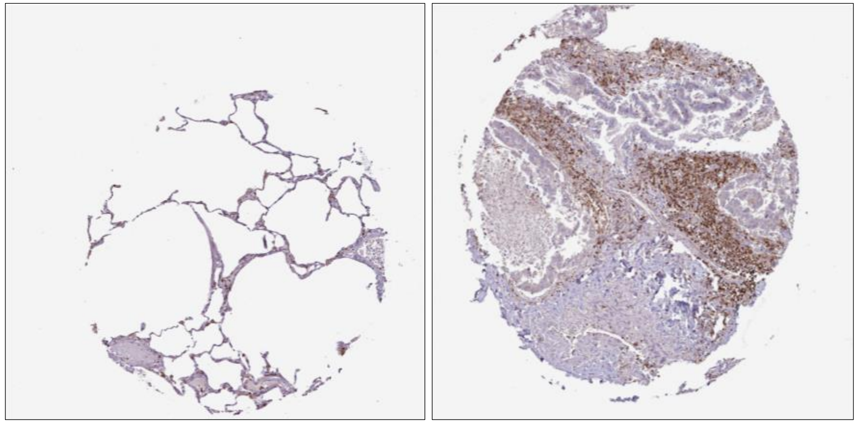

Normal LUAD

Figure S6. Immunohistochemistry analysis of *SEC61G* in the HPA database.

SEC61G		
Tissue	Normal	LUAD
Antibody	HPA053196	
Patient ID	1678	1847
Age	57	64
Gender	Female	Female
N	Alveolar cells	
	Staining	Not detected
	Intensity	Negative
	Quantity	None
	Macrophages	
	Staining	Medium
	Intensity	Moderate
	Quantity	75%-25%
	Location	Cytoplasmic/membranous
LUAD	Tumor cells	
	Staining	Not detected
	Intensity	Negative
	Quantity	None

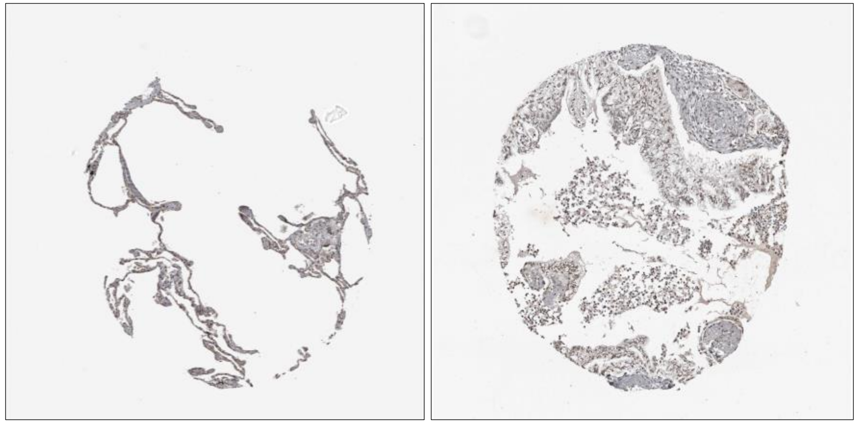

Normal LUAD

Figure S7. Immunohistochemistry analysis of *CHD1L* in the HPA database.

CHD1L		
Tissue	Normal	LUAD
Antibody	HPA027789	
Patient ID	2208	3391
Age	67	70
Gender	Female	Female
N	Alveolar cells	
	Staining	Low
	Intensity	Moderate
	Quantity	<25%
	Location	Cytoplasmic/membranous/nuclear
	Macrophages	
	Staining	Low
	Intensity	Moderate
	Quantity	<25%
	Location	Nuclear
LUAD	Tumor cells	
	Staining	Medium
	Intensity	Moderate
	Quantity	>75%
	Location	Nuclear

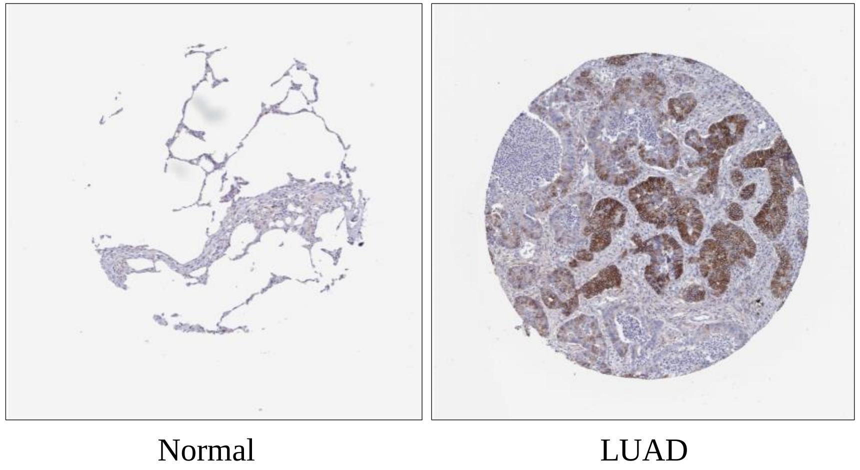

Figure S8. Immunohistochemistry analysis of *CDH2* in the HPA database.

CDH2		
Tissue	Normal	LUAD
Antibody	HPA058574	
Patient ID	1678	1932
Age	57	57
Gender	Female	Female
N	Alveolar cells	
	Staining	Low
	Intensity	Moderate
	Quantity	<25%
	Location	Cytoplasmic/membranous
	Macrophages	
	Staining	Not detected
	Intensity	Negative
	Quantity	None
LUAD	Tumor cells	
	Staining	High
	Intensity	Strong
	Quantity	75%-25%
	Location	Cytoplasmic/membranous

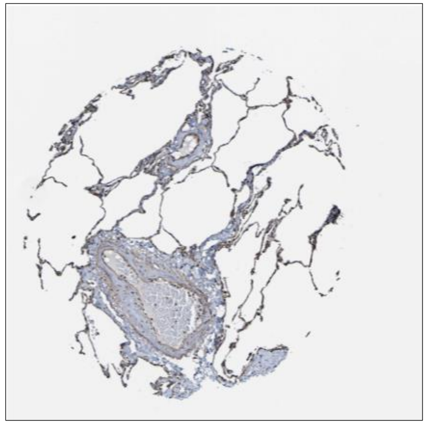

Normal

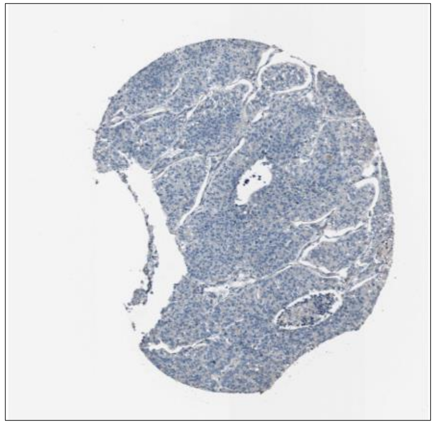

LUAD

Figure S9. Immunohistochemistry analysis of *NAALADL2* in the HPA database.

	NAALADL2	
Tissue	Normal	LUAD
Antibody	HPA012413	
Patient ID	2222	3003
Age	59	49
Gender	Male	Male
N	Alveolar cells	
	Staining	Medium
	Intensity	Moderate
	Quantity	75%-25%
	Location	Cytoplasmic/membranous/nuclear
	Macrophages	
	Staining	High
	Intensity	Strong
	Quantity	>75%
	Location	Cytoplasmic/membranous
LUAD	Tumor cells	
	Staining	Not detected
	Intensity	Negative
	Quantity	None

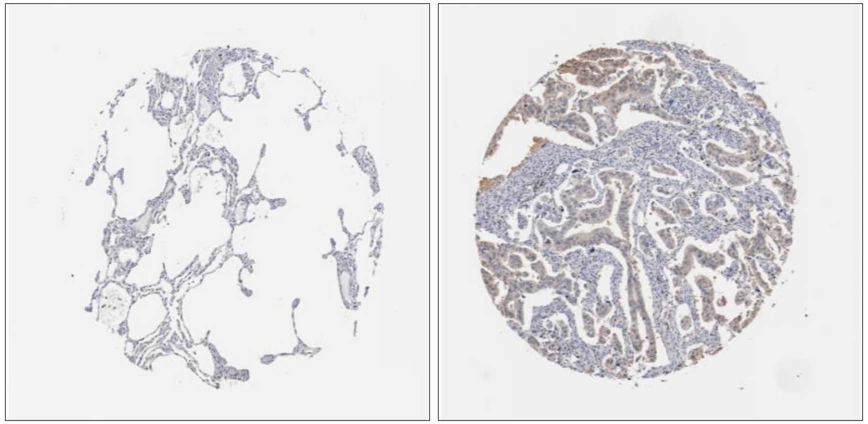

Normal LUAD

Figure S10. Immunohistochemistry analysis of *MIF* in the HPA database.

MIF		
Tissue	Normal	LUAD
Antibody	HPA003868	
Patient ID	2268	426
Age	49	48
Gender	Female	Female
N	Alveolar cells	
	Staining	Not detected
	Intensity	Weak
	Quantity	<25%
	Location	Cytoplasmic/membranous
	Macrophages	
	Staining	Not detected
	Intensity	Negative
	Quantity	None
LUAD	Tumor cells	
	Staining	Low
	Intensity	Weak
	Quantity	75%-25%
	Location	Cytoplasmic/membranous

Supplementary material

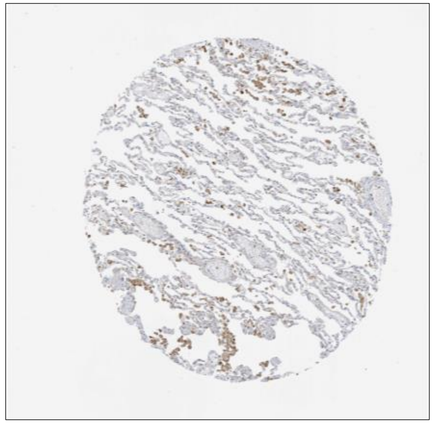

NO data

Normal LUAD

Figure S11. Immunohistochemistry analysis of *CAPN13* in the HPA database.

CAPN13		
Tissue	Normal	LUAD
Antibody	HPA029497	
Patient ID	2268	NO data
Age	49	
Gender	Female	
N	Alveolar cells	
	Staining	Low
	Intensity	Moderate
	Quantity	<25%
	Location	Cytoplasmic/membranous
	Macrophages	
	Staining	Medium
	Intensity	Moderate
	Quantity	>75%
	Location	Cytoplasmic/membranous
LUAD	NO data	

CTLA4		
Tissue	Normal	LUAD
	NO data	NO data

Figure S12. Immunohistochemistry analysis of *CTLA4* in the HPA database.

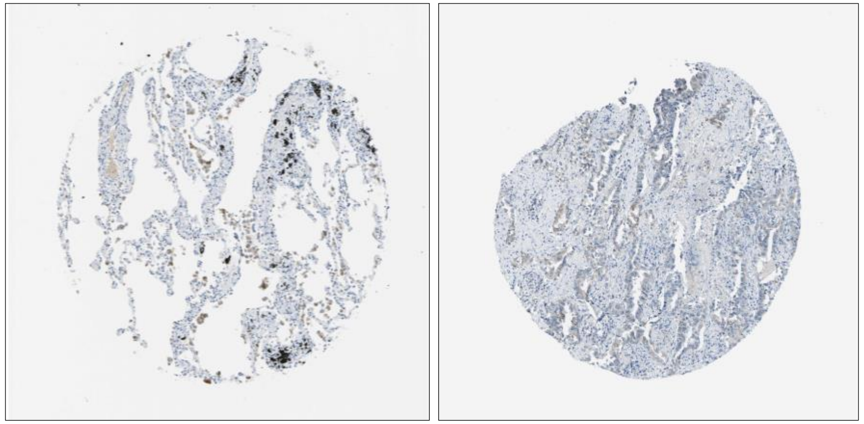

Normal LUAD

Figure S13. Immunohistochemistry analysis of *TM6SF1* in the HPA database.

TM6SF1		
Tissue	Normal	LUAD
Antibody	HPA016051	
Patient ID	2268	3052
Age	49	51
Gender	Female	Female
N	Alveolar cells	
	Staining	Not detected
	Intensity	Weak
	Quantity	<25%
	Location	Cytoplasmic/membranous
	Macrophages	
	Staining	Medium
	Intensity	Moderate
	Quantity	75%-25%
	Location	Cytoplasmic/membranous
LUAD	Tumor cells	
	Staining	Low
	Intensity	Weak
	Quantity	75%-25%
	Location	Cytoplasmic/membranous

Calculation method of C-index

For these reasons, the *concordance index* (CI) or c-index is one of the most commonly used performance measures of survival models, e.g., [6]. It can be interpreted as the fraction of all pairs of subjects whose predicted survival times are correctly ordered among all subjects that can actually be ordered. In other words, it is the probability of concordance between the predicted and the observed survival. It can be written as

$$c(\mathcal{D}, \mathcal{G}, f) = \frac{1}{|\mathcal{E}|} \sum_{\mathcal{E}_{ij}} \mathbf{1}_{f(x_i)<f(x_j)} \tag{4}$$

with the indicator function $\mathbf{1}_{a<b} = 1$ if $a < b$, and 0 otherwise; $|\mathcal{E}|$ denotes the number of edges in the order graph. $f(x_i)$ is the predicted survival time for subject i by the model f. Equivalently, the concordance index can also be written explicitly as

$$c = \frac{1}{|\mathcal{E}|} \sum_{T_i \text{ uncensored}} \sum_{T_j > T_i} \mathbf{1}_{f(x_i)<f(x_j)}. \tag{5}$$

This index is a generalization of the Wilcoxon-Mann-Whitney statistics [13, 10] and thus of the area under the ROC curve (AUC) to regression problems in that it can (1) be applied to continuous

output variables and (2) account for censoring of the data. Like for the AUC, $c = 1$ indicates perfect prediction accuracy and $c = 0.5$ is as good as a random predictor.

Raykar, V. C. , Steck, H. , Krishnapuram, B. , Dehing-Oberije, C. , & Lambin, P. . (2007). On Ranking in Survival Analysis: Bounds on the Concordance Index. Conference on Neural Information Processing Systems. DBLP.
