# Supplementary material for: Clustering analysis and prognostic signature of lung adenocarcinoma based on the tumor microenvironment
Source: Sci Rep. 2022 Jul 14;12:12059. doi: 10.1038/s41598-022-15971-4 (PMC9283441; doi:10.1038/s41598-022-15971-4)
Supplement: Supplementary file 2 — Supplementary Tables. [file 41598_2022_15971_MOESM2_ESM.docx]

**Table S1.** Clinical data and grouping information of 468 cases.

| Id | Futime | Fustat | Age | Gender | Stage | T | M | N | Cluster | Group | Risk |
| --- | --- | --- | --- | --- | --- | --- | --- | --- | --- | --- | --- |
| TCGA-05-4244 | 0 | 0 | 70 | M | IV | T2 | M1 | N2 | C1 | testing | low |
| TCGA-05-4249 | 1523 | 0 | 67 | M | I | T2 | M0 | N0 | C2 | training | high |
| TCGA-05-4250 | 121 | 1 | 79 | F | III | T3 | M0 | N1 | C1 | training | high |
| TCGA-05-4382 | 607 | 0 | 68 | M | I | T2 | M0 | N0 | C2 | training | high |
| TCGA-05-4384 | 426 | 0 | 66 | M | III | T2 | M0 | N2 | C2 | training | low |
| TCGA-05-4389 | 1369 | 0 | 70 | M | I | T1 | M0 | N0 | C1 | testing | low |
| TCGA-05-4390 | 1126 | 0 | 58 | F | I | T2 | M0 | N0 | C1 | training | high |
| TCGA-05-4395 | 0 | 1 | 76 | M | III | T4 | M0 | N2 | C1 | testing | high |
| TCGA-05-4396 | 303 | 1 | 76 | M | III | T4 | M0 | N1 | C2 | training | high |
| TCGA-05-4397 | 731 | 1 | 65 | M | II | T2 | M0 | N1 | C1 | training | high |
| TCGA-05-4398 | 1431 | 0 | 47 | F | III | T4 | M0 | N3 | C1 | training | low |
| TCGA-05-4402 | 244 | 1 | 57 | F | IV | T2 | M1 | NX | C2 | training | high |
| TCGA-05-4403 | 578 | 0 | 76 | M | I | T2 | M0 | N0 | C2 | training | high |
| TCGA-05-4405 | 610 | 0 | 74 | F | I | T2 | M0 | N0 | C2 | testing | low |
| TCGA-05-4410 | 0 | 0 | 62 | M | I | T2 | M0 | N0 | C2 | training | low |
| TCGA-05-4415 | 91 | 1 | 57 | M | III | T4 | M0 | N2 | C1 | testing | high |
| TCGA-05-4417 | 455 | 0 | 51 | F | I | T2 | M0 | N0 | C2 | training | low |
| TCGA-05-4418 | 274 | 1 | 69 | M | III | T3 | M0 | N2 | C1 | training | high |
| TCGA-05-4420 | 912 | 0 | 41 | M | I | T2 | M0 | N0 | C1 | testing | high |
| TCGA-05-4422 | 365 | 0 | 68 | M | I | T2 | M0 | N0 | C2 | training | high |
| TCGA-05-4424 | 913 | 0 | 70 | M | II | T3 | M0 | N0 | C1 | training | low |
| TCGA-05-4425 | 669 | 0 | 70 | F | IV | T2 | M1 | N0 | C1 | training | high |
| TCGA-05-4426 | 791 | 0 | 71 | M | I | T2 | M0 | N0 | C2 | training | low |
| TCGA-05-4427 | 791 | 0 | 65 | F | II | T2 | M0 | N1 | C1 | training | low |
| TCGA-05-4430 | 761 | 0 | 59 | F | I | T2 | M0 | N0 | C2 | training | high |
| TCGA-05-4432 | 761 | 0 | 66 | M | II | T2 | M0 | N1 | C1 | training | high |
| TCGA-05-4433 | 730 | 0 | 82 | M | I | T2 | M0 | N0 | C2 | testing | high |
| TCGA-05-4434 | 457 | 1 | 67 | F | IV | T4 | M1 | N1 | C1 | testing | high |
| TCGA-05-5420 | 457 | 0 | 67 | M | III | T2 | M0 | N2 | C2 | testing | low |
| TCGA-05-5423 | 151 | 0 | 65 | M | II | T2 | M0 | N1 | C2 | testing | low |
| TCGA-05-5425 | 882 | 0 | 68 | M | II | T2 | M0 | N1 | C1 | testing | low |
| TCGA-05-5428 | 670 | 0 | 57 | M | II | T1 | M0 | N1 | C1 | testing | high |
| TCGA-05-5429 | 275 | 1 | 60 | M | III | T3 | M0 | N2 | C1 | training | high |
| TCGA-05-5715 | 62 | 0 | 69 | F | I | T2 | M0 | N0 | C2 | training | high |
| TCGA-35-3615 | 14 | 0 | 57 | M | I | T2 | M0 | N0 | C2 | testing | low |
| TCGA-35-4122 | 225 | 0 | 69 | M | I | T1 | M0 | N0 | C1 | training | low |
| TCGA-35-4123 | 182 | 0 | 38 | M | I | T1 | M0 | N0 | C1 | training | high |
| TCGA-35-5375 | 264 | 0 | 61 | M | III | T2 | M0 | N2 | C1 | testing | high |
| TCGA-38-4625 | 2973 | 0 | 66 | F | I | T2 | M0 | N0 | C1 | training | high |
| TCGA-38-4626 | 3674 | 0 | 57 | F | unknow | T2 | M0 | N0 | C2 | training | low |
| TCGA-38-4627 | 1147 | 1 | 64 | F | II | T1 | M0 | N1 | C2 | testing | low |
| TCGA-38-4628 | 1492 | 1 | 65 | F | II | T2 | M0 | N1 | C2 | training | low |
| TCGA-38-4629 | 864 | 1 | 68 | M | II | T3 | M0 | N0 | C1 | testing | high |
| TCGA-38-4630 | 1073 | 1 | 75 | F | I | T2 | M0 | N0 | C1 | testing | high |
| TCGA-38-4631 | 354 | 1 | 72 | F | I | T2 | M0 | N0 | C1 | testing | high |
| TCGA-38-4632 | 1357 | 1 | 42 | M | IV | T2 | M1 | N1 | C1 | training | high |
| TCGA-38-6178 | 448 | 0 | 70 | F | III | T2 | unknow | N2 | C2 | training | high |
| TCGA-38-7271 | 800 | 1 | 72 | F | I | T1 | M0 | N0 | C2 | testing | low |
| TCGA-44-2655 | 1324 | 0 | 65 | F | I | T1 | M0 | N0 | C2 | testing | high |
| TCGA-44-2656 | 1429 | 0 | 59 | M | I | T2 | M0 | N0 | C2 | training | low |
| TCGA-44-2657 | 1351 | 0 | 74 | F | I | T2 | M0 | NX | C2 | testing | low |
| TCGA-44-2659 | 1367 | 0 | 65 | F | II | T1 | M0 | N1 | C2 | training | low |
| TCGA-44-2661 | 1159 | 0 | 69 | F | I | T1 | M0 | N0 | C2 | training | low |
| TCGA-44-2662 | 1280 | 0 | 65 | M | I | T2 | M0 | N0 | C1 | training | low |
| TCGA-44-2665 | 1301 | 0 | 55 | F | II | T2 | M0 | N1 | C2 | training | low |
| TCGA-44-2666 | 97 | 1 | 43 | M | I | T2 | M0 | N0 | C2 | testing | high |
| TCGA-44-2668 | 761 | 1 | 51 | M | I | T2 | M0 | N0 | C1 | training | high |
| TCGA-44-3396 | 1130 | 0 | 74 | F | III | T2 | M0 | N2 | C1 | training | low |
| TCGA-44-3398 | 1163 | 0 | 77 | F | I | T1 | M0 | N0 | C2 | training | low |
| TCGA-44-3917 | 1183 | 0 | 33 | F | I | T2 | M0 | N0 | C1 | training | low |
| TCGA-44-3918 | 1036 | 0 | 60 | F | I | T1 | M0 | N0 | C2 | training | low |
| TCGA-44-3919 | 1026 | 1 | 71 | F | I | T1 | M0 | N0 | C2 | training | low |
| TCGA-44-4112 | 808 | 1 | 60 | F | I | T2 | M0 | N0 | C2 | training | low |
| TCGA-44-5643 | 1013 | 0 | 53 | M | III | T2 | M0 | N2 | C1 | training | low |
| TCGA-44-5644 | 863 | 0 | 51 | F | I | T2 | unknow | N0 | C1 | training | low |
| TCGA-44-5645 | 852 | 0 | 61 | F | I | T1 | unknow | NX | C2 | training | low |
| TCGA-44-6145 | 595 | 0 | 62 | F | I | T1 | M0 | N0 | C1 | testing | high |
| TCGA-44-6147 | 845 | 0 | 67 | F | I | T1 | M0 | NX | C2 | training | low |
| TCGA-44-6774 | 658 | 0 | 56 | F | III | T1 | M0 | N2 | C1 | training | low |
| TCGA-44-6775 | 705 | 0 | 72 | F | I | T2 | MX | N0 | C2 | training | low |
| TCGA-44-6776 | 2616 | 0 | 60 | F | I | T1 | MX | N0 | C2 | training | low |
| TCGA-44-6777 | 987 | 1 | 85 | F | I | T2 | MX | NX | C2 | testing | low |
| TCGA-44-6778 | 1864 | 0 | 59 | M | I | T1 | MX | N0 | C1 | training | low |
| TCGA-44-6779 | 500 | 1 | 50 | F | II | T2 | MX | N1 | C1 | training | high |
| TCGA-44-7659 | 691 | 0 | 70 | M | I | T1 | MX | N0 | C2 | testing | high |
| TCGA-44-7660 | 592 | 0 | 72 | M | I | T2 | MX | N0 | C1 | testing | high |
| TCGA-44-7661 | 557 | 1 | 69 | F | I | T2 | M0 | N0 | C1 | testing | high |
| TCGA-44-7662 | 218 | 0 | 61 | M | I | T2 | MX | N0 | C1 | training | high |
| TCGA-44-7667 | 1097 | 0 | 49 | F | II | T3 | MX | N0 | C1 | testing | high |
| TCGA-44-7669 | 574 | 1 | 59 | M | II | T1 | MX | N1 | C1 | training | high |
| TCGA-44-7670 | 882 | 0 | 47 | F | II | T1 | M0 | N1 | C1 | training | low |
| TCGA-44-7671 | 889 | 0 | 64 | M | I | T2 | M0 | N0 | C2 | training | high |
| TCGA-44-7672 | 719 | 0 | 52 | F | I | T1 | M0 | N0 | C1 | training | low |
| TCGA-44-8117 | 385 | 0 | 54 | F | I | T2 | M0 | N0 | C1 | training | low |
| TCGA-44-8119 | 285 | 0 | 73 | M | II | T3 | M0 | N0 | C1 | training | low |
| TCGA-44-8120 | 260 | 0 | 58 | M | I | T2 | M0 | N0 | C2 | training | low |
| TCGA-44-A479 | 486 | 0 | 73 | F | I | T2 | MX | N0 | C1 | testing | low |
| TCGA-44-A47A | 466 | 0 | 78 | F | I | T2 | MX | N0 | C2 | training | high |
| TCGA-44-A47B | 287 | 0 | 79 | M | I | T2 | M0 | N0 | C2 | testing | low |
| TCGA-44-A47G | 351 | 0 | 73 | F | I | T1 | M0 | N0 | C2 | training | low |
| TCGA-44-A4SS | 415 | 0 | 73 | M | I | T1 | M0 | N0 | C1 | training | high |
| TCGA-44-A4SU | 409 | 1 | 67 | F | I | T1 | MX | N0 | C2 | training | low |
| TCGA-49-4486 | 2318 | 1 | 72 | M | I | T1 | M0 | N0 | C2 | training | low |
| TCGA-49-4487 | 855 | 1 | 72 | F | I | T1 | M0 | N0 | C1 | testing | low |
| TCGA-49-4488 | 869 | 1 | 74 | F | I | T1 | MX | N0 | C1 | testing | high |
| TCGA-49-4490 | 385 | 1 | 45 | F | III | T3 | M0 | N2 | C2 | testing | high |
| TCGA-49-4494 | 1081 | 1 | 77 | M | III | T3 | M0 | N2 | C1 | training | high |
| TCGA-49-4501 | 1421 | 1 | 67 | F | I | T2 | M0 | N0 | C2 | training | high |
| TCGA-49-4505 | 428 | 1 | 61 | F | II | T2 | M0 | N1 | C2 | training | low |
| TCGA-49-4506 | 999 | 1 | 68 | F | II | T2 | M0 | N1 | C1 | training | high |
| TCGA-49-4507 | 268 | 1 | 73 | F | III | T3 | M0 | N1 | C1 | training | high |
| TCGA-49-4510 | 896 | 1 | 51 | F | II | T2 | M0 | N1 | C2 | training | low |
| TCGA-49-4512 | 905 | 1 | 69 | F | III | T2 | MX | N2 | C2 | testing | high |
| TCGA-49-4514 | 1700 | 0 | 79 | F | I | T1 | M0 | N0 | C1 | training | high |
| TCGA-49-6743 | 1621 | 0 | 81 | F | III | T1 | MX | N2 | C1 | training | high |
| TCGA-49-6744 | 1683 | 0 | 64 | F | II | T2 | MX | N1 | C2 | training | low |
| TCGA-49-6745 | 522 | 0 | 82 | M | III | T2 | M0 | N2 | C1 | training | high |
| TCGA-49-6761 | 354 | 0 | 68 | F | III | T1 | MX | N2 | C1 | testing | high |
| TCGA-49-6767 | 677 | 0 | 46 | F | II | T3 | MX | N0 | C1 | testing | high |
| TCGA-49-AAQV | 677 | 1 | 63 | F | II | T1 | MX | N1 | C1 | testing | high |
| TCGA-49-AAR0 | 4765 | 0 | 57 | M | I | T1 | MX | N0 | C2 | testing | high |
| TCGA-49-AAR3 | 1893 | 0 | 69 | M | II | T2 | MX | N1 | C1 | training | low |
| TCGA-49-AAR4 | 879 | 1 | 51 | M | III | T2 | MX | N2 | C1 | testing | low |
| TCGA-49-AAR9 | 260 | 1 | 61 | M | II | T3 | MX | N0 | C1 | training | high |
| TCGA-49-AARE | 1229 | 1 | 51 | F | I | T1 | MX | N0 | C1 | training | low |
| TCGA-49-AARN | 1135 | 1 | 56 | F | I | T1 | MX | N0 | C2 | training | high |
| TCGA-49-AARO | 3759 | 0 | 39 | F | I | T1 | MX | N0 | C2 | training | low |
| TCGA-49-AARQ | 6732 | 0 | 41 | F | I | T2 | MX | N0 | C1 | training | low |
| TCGA-49-AARR | 4992 | 0 | 68 | M | I | T1 | MX | N0 | C2 | testing | low |
| TCGA-4B-A93V | 300 | 1 | 52 | F | I | T1 | M0 | N0 | C1 | training | high |
| TCGA-50-5044 | 624 | 1 | 72 | F | III | T4 | M0 | N1 | C1 | training | high |
| TCGA-50-5045 | 2174 | 1 | 57 | F | unknow | T2 | M0 | N1 | C2 | training | low |
| TCGA-50-5049 | 3094 | 0 | 70 | M | I | T2 | M0 | N0 | C2 | training | low |
| TCGA-50-5051 | 478 | 1 | 42 | F | III | T2 | M0 | N2 | C1 | training | high |
| TCGA-50-5055 | 1830 | 1 | 79 | F | II | T1 | M0 | N1 | C2 | training | low |
| TCGA-50-5066 | 1442 | 0 | 72 | M | I | T2 | M0 | N0 | C1 | testing | high |
| TCGA-50-5068 | 1499 | 1 | 59 | F | II | T2 | MX | N1 | C2 | testing | low |
| TCGA-50-5072 | 250 | 1 | 74 | M | III | T2 | M0 | N2 | C1 | training | high |
| TCGA-50-5930 | 282 | 1 | 47 | M | III | T2 | M0 | N2 | C1 | training | low |
| TCGA-50-5931 | 434 | 1 | 75 | F | I | T2 | M0 | N0 | C1 | training | high |
| TCGA-50-5932 | 1235 | 1 | 75 | M | II | T2 | M0 | N1 | C2 | training | high |
| TCGA-50-5933 | 2393 | 1 | 72 | M | III | T4 | M0 | N2 | C1 | training | high |
| TCGA-50-5935 | 653 | 1 | 86 | F | I | T1 | M0 | N0 | C2 | testing | low |
| TCGA-50-5939 | 460 | 1 | 85 | M | I | T2 | M0 | N0 | C1 | training | high |
| TCGA-50-5941 | 1474 | 0 | 55 | F | III | T2 | M0 | N2 | C2 | training | low |
| TCGA-50-5942 | 1847 | 0 | 67 | F | I | T1 | M0 | N0 | C2 | training | low |
| TCGA-50-5946 | 1617 | 0 | 62 | M | I | T1 | MX | N0 | C1 | training | high |
| TCGA-50-6590 | 1288 | 1 | 72 | F | I | T2 | M0 | N0 | C1 | testing | high |
| TCGA-50-6591 | 119 | 1 | 63 | F | IV | T2 | M1 | N0 | C1 | training | high |
| TCGA-50-6592 | 777 | 1 | 71 | F | I | T2 | M0 | N0 | C1 | training | high |
| TCGA-50-6593 | 336 | 1 | 49 | F | III | T1 | M0 | N2 | C1 | testing | high |
| TCGA-50-6594 | 370 | 1 | 79 | F | III | T3 | M0 | N2 | C1 | training | high |
| TCGA-50-6595 | 189 | 1 | 74 | F | III | T2 | M0 | N2 | C1 | training | high |
| TCGA-50-6597 | 1268 | 1 | 79 | F | I | T2 | M0 | N0 | C2 | training | low |
| TCGA-50-6673 | 22 | 1 | 84 | F | I | T1 | M0 | N0 | C2 | training | high |
| TCGA-50-7109 | 308 | 1 | 60 | M | I | T1 | M0 | N0 | C1 | training | high |
| TCGA-50-8457 | 1125 | 0 | 63 | F | I | T1 | M0 | N0 | C2 | testing | low |
| TCGA-50-8460 | 829 | 0 | 74 | M | I | T1 | M0 | N0 | C2 | testing | low |
| TCGA-53-7624 | 1043 | 1 | 40 | F | IV | T2 | M1 | N0 | C1 | testing | high |
| TCGA-53-7626 | 929 | 1 | 76 | F | II | T1 | M0 | N1 | C2 | testing | low |
| TCGA-53-7813 | 424 | 0 | 51 | F | III | T4 | M0 | N0 | C1 | training | low |
| TCGA-55-1592 | 701 | 1 | 65 | M | I | T2 | M0 | N0 | C2 | training | high |
| TCGA-55-1594 | 1178 | 0 | 68 | M | III | T2 | M0 | N2 | C1 | training | low |
| TCGA-55-1596 | 2065 | 0 | 55 | M | II | T2 | M0 | N1 | C1 | training | high |
| TCGA-55-5899 | 930 | 0 | 58 | M | unknow | T1 | M0 | N1 | C1 | testing | high |
| TCGA-55-6543 | 435 | 0 | 60 | F | I | T1 | MX | N0 | C2 | training | high |
| TCGA-55-6642 | 2449 | 0 | 63 | M | I | T2 | MX | N0 | C1 | testing | low |
| TCGA-55-6712 | 171 | 1 | 71 | M | II | T2 | MX | N1 | C1 | training | high |
| TCGA-55-6968 | 1293 | 1 | 61 | M | IV | T1 | M1 | N0 | C1 | training | high |
| TCGA-55-6970 | 464 | 1 | 67 | F | III | T2 | MX | N2 | C1 | training | high |
| TCGA-55-6971 | 1400 | 0 | 59 | F | I | T2 | MX | N0 | C2 | training | low |
| TCGA-55-6972 | 1632 | 1 | 72 | M | I | T2 | M0 | N0 | C2 | training | low |
| TCGA-55-6975 | 118 | 1 | 61 | M | II | T2 | M0 | N1 | C1 | testing | high |
| TCGA-55-6978 | 176 | 1 | 81 | M | II | T2 | MX | N0 | C1 | training | low |
| TCGA-55-6979 | 237 | 1 | 59 | F | II | T2 | M0 | N1 | C1 | testing | low |
| TCGA-55-6980 | 2109 | 0 | 56 | M | I | T1 | M0 | N0 | C2 | training | high |
| TCGA-55-6981 | 1379 | 1 | 53 | F | III | T1 | M0 | N2 | C1 | training | high |
| TCGA-55-6982 | 995 | 1 | 79 | F | II | T2 | M0 | N1 | C1 | training | high |
| TCGA-55-6984 | 760 | 1 | 71 | F | II | T2 | M0 | N1 | C1 | training | high |
| TCGA-55-6985 | 1233 | 0 | 58 | F | I | T2 | MX | N0 | C2 | training | low |
| TCGA-55-6986 | 3261 | 0 | 74 | F | I | T2 | M0 | N0 | C2 | testing | high |
| TCGA-55-6987 | 2137 | 0 | 77 | M | I | T1 | M0 | N0 | C1 | training | low |
| TCGA-55-7227 | 952 | 1 | 77 | M | III | T3 | MX | N1 | C2 | testing | high |
| TCGA-55-7281 | 872 | 0 | 70 | F | I | T1 | M0 | N0 | C2 | testing | low |
| TCGA-55-7283 | 609 | 0 | 76 | F | III | T3 | MX | N2 | C2 | training | high |
| TCGA-55-7284 | 243 | 1 | 74 | M | II | T3 | MX | N0 | C2 | training | high |
| TCGA-55-7570 | 824 | 0 | 60 | M | I | T1 | MX | N0 | C1 | training | high |
| TCGA-55-7573 | 487 | 0 | 72 | F | I | T1 | MX | N0 | C2 | training | low |
| TCGA-55-7574 | 995 | 1 | 64 | F | I | T2 | M0 | N0 | C2 | training | low |
| TCGA-55-7576 | 670 | 0 | 54 | M | I | T2 | M0 | N0 | C1 | testing | low |
| TCGA-55-7724 | 705 | 0 | 76 | F | I | T2 | MX | N0 | C1 | training | low |
| TCGA-55-7725 | 442 | 0 | 68 | F | I | T1 | MX | N0 | C2 | training | low |
| TCGA-55-7726 | 652 | 0 | 72 | F | I | T1 | MX | N0 | C1 | training | high |
| TCGA-55-7727 | 119 | 0 | 70 | M | III | T1 | MX | N2 | C1 | testing | low |
| TCGA-55-7815 | 773 | 0 | 76 | M | I | T2 | MX | N0 | C1 | testing | low |
| TCGA-55-7816 | 468 | 1 | 49 | F | IV | TX | MX | NX | C2 | testing | low |
| TCGA-55-7903 | 567 | 0 | 64 | M | I | T1 | MX | N0 | C1 | testing | high |
| TCGA-55-7907 | 343 | 1 | 77 | M | II | T2 | MX | N1 | C1 | training | high |
| TCGA-55-7910 | 1040 | 0 | 50 | F | II | T2 | M0 | N0 | C1 | training | high |
| TCGA-55-7911 | 537 | 0 | 70 | F | I | T1 | MX | N0 | C1 | training | low |
| TCGA-55-7913 | 561 | 1 | 61 | F | I | T1 | MX | N0 | C1 | testing | high |
| TCGA-55-7914 | 187 | 1 | 71 | F | II | T1 | MX | N1 | C2 | training | high |
| TCGA-55-7994 | 603 | 0 | 81 | M | II | T3 | MX | N0 | C1 | training | high |
| TCGA-55-7995 | 889 | 0 | 73 | F | I | T1 | M0 | N0 | C1 | training | low |
| TCGA-55-8085 | 904 | 0 | 64 | M | I | T1 | M0 | N0 | C1 | training | low |
| TCGA-55-8087 | 462 | 0 | 59 | F | I | T2 | MX | N0 | C2 | testing | low |
| TCGA-55-8089 | 702 | 1 | 56 | M | I | T1 | M0 | N0 | C1 | training | high |
| TCGA-55-8090 | 598 | 1 | 80 | M | I | T1 | M0 | N0 | C2 | training | high |
| TCGA-55-8091 | 600 | 0 | 74 | M | I | T2 | MX | N0 | C2 | training | low |
| TCGA-55-8092 | 154 | 1 | 75 | M | II | T3 | MX | N0 | C1 | training | high |
| TCGA-55-8094 | 541 | 0 | 51 | M | IV | T2 | M1 | N0 | C1 | testing | high |
| TCGA-55-8096 | 719 | 1 | 67 | F | I | T2 | MX | N0 | C2 | training | high |
| TCGA-55-8097 | 476 | 0 | 60 | F | I | T1 | MX | N0 | C2 | training | low |
| TCGA-55-8203 | 547 | 0 | 69 | F | I | T1 | M0 | N0 | C1 | training | high |
| TCGA-55-8204 | 515 | 0 | 87 | F | I | T2 | MX | N0 | C1 | testing | low |
| TCGA-55-8205 | 599 | 0 | 76 | F | II | T2 | M0 | N0 | C1 | training | high |
| TCGA-55-8206 | 888 | 0 | 56 | M | I | T1 | M0 | N0 | C2 | training | low |
| TCGA-55-8208 | 674 | 0 | 73 | F | I | T1 | M0 | N0 | C2 | training | low |
| TCGA-55-8299 | 469 | 1 | 61 | F | I | T1 | MX | N0 | C1 | testing | low |
| TCGA-55-8301 | 534 | 0 | 58 | M | I | T2 | MX | N0 | C1 | testing | low |
| TCGA-55-8302 | 478 | 0 | 54 | M | I | T2 | MX | N0 | C1 | training | high |
| TCGA-55-8505 | 440 | 0 | 62 | M | III | T1 | MX | N2 | C1 | testing | high |
| TCGA-55-8506 | 11 | 0 | 62 | F | II | T3 | MX | N0 | C1 | training | low |
| TCGA-55-8507 | 418 | 0 | 53 | M | I | T1 | MX | N0 | C1 | training | high |
| TCGA-55-8510 | 539 | 0 | 55 | F | I | T2 | MX | N0 | C2 | training | low |
| TCGA-55-8511 | 552 | 0 | 73 | F | I | T2 | MX | N0 | C1 | training | high |
| TCGA-55-8512 | 607 | 1 | 41 | M | IV | T1 | M1 | N1 | C2 | training | low |
| TCGA-55-8514 | 520 | 0 | 70 | F | I | T2 | MX | N0 | C2 | training | low |
| TCGA-55-8614 | 536 | 0 | 76 | M | I | T2 | MX | N0 | C1 | testing | low |
| TCGA-55-8615 | 446 | 0 | 67 | M | III | T3 | MX | N2 | C1 | training | high |
| TCGA-55-8616 | 48 | 0 | 58 | F | I | T2 | M0 | N0 | C2 | testing | low |
| TCGA-55-8620 | 375 | 1 | 60 | M | IV | T1 | M1 | N1 | C1 | testing | high |
| TCGA-55-8621 | 515 | 0 | 75 | F | I | T1 | MX | N0 | C2 | testing | low |
| TCGA-55-A48X | 689 | 0 | 63 | F | II | T1 | M0 | N1 | C2 | training | low |
| TCGA-55-A48Y | 630 | 0 | 69 | M | II | T2 | M0 | N0 | C1 | training | high |
| TCGA-55-A48Z | 651 | 0 | 60 | F | III | T1 | MX | N3 | C1 | training | high |
| TCGA-55-A490 | 99 | 1 | 78 | M | II | T2 | MX | N0 | C1 | training | low |
| TCGA-55-A491 | 626 | 0 | 81 | F | I | T1 | MX | N0 | C2 | training | low |
| TCGA-55-A492 | 596 | 0 | 70 | F | I | T1 | MX | N0 | C2 | training | low |
| TCGA-55-A493 | 28 | 0 | 54 | F | I | T2 | M0 | N0 | C1 | training | low |
| TCGA-55-A494 | 481 | 0 | 61 | F | I | T2 | MX | N0 | C1 | training | high |
| TCGA-55-A4DF | 440 | 1 | 88 | M | I | T1 | MX | N0 | C1 | training | high |
| TCGA-55-A4DG | 608 | 0 | 71 | M | I | T1 | MX | N0 | C2 | training | low |
| TCGA-55-A57B | 546 | 0 | 80 | F | I | T1 | M0 | N0 | C2 | training | high |
| TCGA-62-8394 | 139 | 1 | 65 | F | III | T4 | M0 | N2 | C1 | training | high |
| TCGA-62-8395 | 1216 | 0 | 80 | F | II | T3 | M0 | N0 | C2 | training | low |
| TCGA-62-8397 | 1289 | 0 | 70 | F | II | T3 | M0 | N0 | C2 | training | low |
| TCGA-62-8398 | 444 | 1 | 55 | M | III | T2 | M0 | N2 | C1 | training | high |
| TCGA-62-8399 | 2696 | 0 | 62 | M | III | T2 | M0 | N2 | C1 | testing | low |
| TCGA-62-8402 | 1498 | 1 | 73 | F | III | T2 | M0 | N2 | C1 | training | low |
| TCGA-62-A46O | 1454 | 1 | 65 | F | I | T2 | M0 | N0 | C1 | training | high |
| TCGA-62-A46P | 594 | 1 | 65 | M | I | T2 | M0 | N0 | C2 | training | high |
| TCGA-62-A46R | 1725 | 1 | 54 | F | I | T2 | M0 | N0 | C2 | training | low |
| TCGA-62-A46S | 1653 | 1 | 73 | M | I | T2 | M0 | N0 | C2 | training | high |
| TCGA-62-A46V | 2199 | 0 | 78 | F | I | T2 | M0 | N0 | C1 | testing | high |
| TCGA-62-A46Y | 414 | 1 | 70 | F | III | T2 | M0 | N2 | C2 | training | low |
| TCGA-62-A471 | 1246 | 0 | 64 | M | II | T2 | M0 | N1 | C1 | testing | high |
| TCGA-62-A472 | 910 | 0 | 70 | M | II | T3 | M0 | N0 | C1 | training | high |
| TCGA-64-1676 | 1728 | 0 | 58 | M | I | T1 | M0 | N0 | C1 | training | high |
| TCGA-64-1677 | 628 | 1 | 77 | F | III | T2 | M0 | N2 | C1 | training | high |
| TCGA-64-1678 | 1189 | 0 | 70 | F | unknow | T2 | M0 | N0 | C1 | training | high |
| TCGA-64-1679 | 2488 | 0 | 58 | F | III | T1 | M0 | N2 | C1 | training | low |
| TCGA-64-1680 | 1126 | 0 | 63 | M | IV | T2 | M1 | N2 | C2 | training | low |
| TCGA-64-1681 | 1167 | 1 | 61 | F | I | T1 | M0 | N0 | C2 | training | high |
| TCGA-64-5774 | 2676 | 0 | 60 | M | I | T2 | M0 | N0 | C1 | training | high |
| TCGA-64-5775 | 62 | 1 | 71 | M | III | T4 | M0 | N0 | C1 | training | high |
| TCGA-64-5778 | 1305 | 0 | 60 | M | I | T2 | M0 | N0 | C2 | training | low |
| TCGA-64-5781 | 1559 | 0 | 55 | F | I | T2 | M0 | N0 | C1 | testing | high |
| TCGA-64-5815 | 866 | 0 | 74 | M | II | T2 | M0 | N1 | C2 | training | low |
| TCGA-67-3770 | 610 | 0 | 70 | F | I | T1 | M0 | N0 | C2 | training | low |
| TCGA-67-3771 | 610 | 0 | 77 | F | I | T1 | M0 | N0 | C1 | training | low |
| TCGA-67-3772 | 573 | 0 | 82 | F | I | T2 | M0 | N0 | C2 | training | low |
| TCGA-67-3773 | 427 | 0 | 84 | F | I | T2 | M0 | N0 | C2 | training | low |
| TCGA-67-3774 | 385 | 0 | 73 | F | I | T2 | M0 | N0 | C2 | testing | low |
| TCGA-67-4679 | 448 | 0 | 69 | M | unknow | T3 | M0 | N0 | C2 | training | low |
| TCGA-67-6215 | 174 | 0 | 52 | F | I | T2 | M0 | N0 | C2 | training | high |
| TCGA-67-6216 | 141 | 0 | 57 | F | I | T1 | M0 | N0 | C2 | training | high |
| TCGA-67-6217 | 422 | 0 | 73 | F | II | T2 | M0 | N1 | C2 | training | low |
| TCGA-69-7760 | 202 | 0 | 73 | M | II | T3 | M0 | N0 | C1 | testing | high |
| TCGA-69-7763 | 690 | 0 | 69 | M | I | T1 | M0 | N0 | C2 | training | high |
| TCGA-69-7764 | 414 | 0 | 75 | M | I | T1 | M0 | N0 | C2 | training | low |
| TCGA-69-7765 | 165 | 0 | 56 | M | unknow | T4 | MX | N0 | C2 | training | low |
| TCGA-69-7973 | 230 | 0 | 42 | F | I | T2 | M0 | N0 | C1 | training | low |
| TCGA-69-7974 | 184 | 0 | 54 | F | III | T2 | MX | N2 | C1 | training | low |
| TCGA-69-7978 | 134 | 0 | 59 | M | II | T2 | MX | N1 | C1 | testing | low |
| TCGA-69-7980 | 411 | 0 | 70 | F | I | T1 | M0 | N0 | C1 | training | high |
| TCGA-69-8253 | 426 | 0 | 59 | F | II | T1 | MX | N1 | C2 | training | low |
| TCGA-69-8254 | 409 | 0 | 85 | M | unknow | T2 | unknow | unknow | C2 | training | high |
| TCGA-69-8255 | 129 | 0 | 71 | M | I | T1 | M0 | N0 | C1 | training | high |
| TCGA-69-A59K | 591 | 0 | 60 | F | II | T3 | M0 | N0 | C1 | testing | low |
| TCGA-71-6725 | 256 | 0 | 48 | F | I | T2 | M0 | N0 | C1 | training | high |
| TCGA-71-8520 | 210 | 1 | 60 | F | I | T2 | M0 | N0 | C1 | training | high |
| TCGA-73-4658 | 1600 | 1 | 80 | F | I | T2 | M0 | N0 | C2 | testing | low |
| TCGA-73-4659 | 711 | 1 | 66 | M | III | T2 | M0 | N2 | C1 | training | low |
| TCGA-73-4662 | 2515 | 0 | 65 | F | I | T1 | M0 | N0 | C2 | training | low |
| TCGA-73-4666 | 800 | 0 | 52 | F | IV | T1 | M1 | N0 | C1 | testing | high |
| TCGA-73-4668 | 467 | 0 | 66 | F | II | T2 | M0 | N1 | C1 | training | high |
| TCGA-73-4670 | 131 | 0 | 69 | F | IV | T2 | M1 | N0 | C1 | training | high |
| TCGA-73-4675 | 922 | 1 | 59 | M | III | T3 | M0 | N1 | C2 | training | high |
| TCGA-73-4676 | 281 | 1 | 45 | M | II | T2 | M0 | N1 | C1 | training | high |
| TCGA-73-4677 | 38 | 1 | 74 | M | unknow | T2 | M0 | N0 | C2 | training | low |
| TCGA-73-7498 | 1189 | 0 | 58 | F | I | T1 | M0 | N0 | C2 | training | low |
| TCGA-73-7499 | 1531 | 1 | 81 | F | I | T2 | M0 | N0 | C1 | training | high |
| TCGA-73-A9RS | 340 | 1 | 41 | M | II | T3 | M0 | N0 | C1 | testing | high |
| TCGA-75-5125 | 2027 | 1 | unknow | M | II | T2 | M0 | N1 | C1 | training | high |
| TCGA-75-5146 | 2368 | 0 | unknow | M | I | T2 | M0 | N0 | C2 | training | low |
| TCGA-75-5147 | 1333 | 0 | unknow | F | I | T2 | M0 | N0 | C1 | testing | low |
| TCGA-75-6206 | 2590 | 0 | unknow | M | I | T2 | M0 | N0 | C2 | testing | low |
| TCGA-75-6212 | 1516 | 1 | unknow | F | II | T2 | M0 | N1 | C2 | training | low |
| TCGA-75-6214 | 1115 | 1 | unknow | F | III | T2 | M0 | N2 | C1 | training | high |
| TCGA-75-7025 | 3305 | 0 | unknow | M | I | T2 | M0 | N0 | C2 | testing | low |
| TCGA-75-7027 | 3059 | 0 | unknow | M | I | T2 | M0 | N0 | C1 | testing | high |
| TCGA-78-7143 | 4961 | 1 | 62 | F | I | T2 | M0 | N0 | C1 | testing | high |
| TCGA-78-7145 | 826 | 1 | 52 | F | IV | T4 | M1 | N1 | C1 | training | high |
| TCGA-78-7146 | 173 | 1 | 71 | F | III | T2 | M0 | N2 | C1 | training | high |
| TCGA-78-7147 | 586 | 1 | 67 | F | II | T2 | M0 | N1 | C1 | training | high |
| TCGA-78-7148 | 626 | 1 | 71 | M | II | T2 | M0 | N1 | C1 | training | high |
| TCGA-78-7149 | 3940 | 0 | 71 | M | III | T4 | M0 | N0 | C2 | training | low |
| TCGA-78-7150 | 666 | 1 | 59 | M | II | T2 | M0 | N1 | C1 | testing | high |
| TCGA-78-7152 | 1215 | 1 | 65 | M | I | T2 | M0 | N0 | C2 | training | high |
| TCGA-78-7153 | 3635 | 0 | 65 | F | I | T2 | M0 | N0 | C1 | testing | high |
| TCGA-78-7154 | 593 | 1 | 72 | M | III | T3 | M0 | N2 | C1 | training | high |
| TCGA-78-7155 | 1171 | 1 | 68 | M | I | T2 | M0 | N0 | C1 | training | high |
| TCGA-78-7156 | 976 | 1 | 62 | M | IV | T4 | M1 | N1 | C2 | testing | low |
| TCGA-78-7158 | 179 | 1 | 59 | F | III | T4 | M0 | N2 | C2 | training | high |
| TCGA-78-7159 | 1974 | 0 | 60 | F | I | T1 | M0 | NX | C1 | training | low |
| TCGA-78-7160 | 697 | 1 | 61 | M | IV | T4 | M1 | N2 | C1 | training | high |
| TCGA-78-7161 | 291 | 1 | 69 | F | II | T3 | M0 | N0 | C2 | testing | high |
| TCGA-78-7162 | 3169 | 1 | 75 | M | I | T1 | M0 | N0 | C2 | training | low |
| TCGA-78-7163 | 7248 | 0 | 60 | M | I | T2 | M0 | N0 | C2 | training | low |
| TCGA-78-7166 | 258 | 1 | 84 | M | II | T2 | M0 | N1 | C1 | training | high |
| TCGA-78-7167 | 2681 | 1 | 77 | M | IV | T2 | M1 | N0 | C2 | testing | high |
| TCGA-78-7220 | 807 | 1 | 53 | F | III | T2 | M0 | N2 | C1 | training | high |
| TCGA-78-7535 | 949 | 1 | 45 | M | I | T2 | M0 | N0 | C1 | training | high |
| TCGA-78-7536 | 244 | 1 | 69 | M | III | T2 | M0 | N2 | C1 | training | high |
| TCGA-78-7537 | 1622 | 1 | 72 | M | I | T2 | M0 | N0 | C2 | training | low |
| TCGA-78-7539 | 791 | 0 | 75 | F | II | T2 | M0 | N0 | C2 | training | low |
| TCGA-78-7540 | 1197 | 1 | 66 | F | I | T2 | M0 | N0 | C2 | training | high |
| TCGA-78-7542 | 321 | 1 | 56 | M | I | T2 | M0 | N0 | C1 | testing | high |
| TCGA-78-7633 | 1528 | 1 | 67 | M | I | T2 | M0 | N0 | C2 | training | low |
| TCGA-78-8640 | 7062 | 0 | 59 | M | II | T1 | M0 | N1 | C1 | training | low |
| TCGA-78-8648 | 1209 | 1 | 58 | F | II | T3 | M0 | N0 | C2 | testing | low |
| TCGA-78-8655 | 2360 | 0 | 77 | F | I | T1 | M0 | N0 | C2 | training | low |
| TCGA-78-8660 | 321 | 1 | 69 | M | II | T2 | M0 | N1 | C1 | training | low |
| TCGA-78-8662 | 3361 | 1 | 53 | F | I | T2 | M0 | N0 | C1 | training | high |
| TCGA-80-5608 | 2832 | 0 | unknow | F | I | T1 | M0 | N0 | C1 | training | high |
| TCGA-80-5611 | 2595 | 0 | unknow | M | I | T2 | M0 | N0 | C1 | testing | low |
| TCGA-83-5908 | 824 | 0 | 59 | F | I | T1 | M0 | N0 | C1 | training | high |
| TCGA-86-6562 | 376 | 1 | 52 | M | II | T2 | M0 | N1 | C1 | training | high |
| TCGA-86-6851 | 179 | 0 | 73 | F | II | T1 | M0 | N1 | C2 | training | low |
| TCGA-86-7701 | 947 | 0 | 66 | M | IV | T2 | M1 | N0 | C1 | training | high |
| TCGA-86-7711 | 1046 | 1 | 70 | M | II | T2 | M0 | N1 | C1 | training | low |
| TCGA-86-7713 | 1157 | 0 | 70 | M | II | T2 | M0 | N0 | C1 | testing | high |
| TCGA-86-7714 | 625 | 1 | 61 | F | III | T1 | M0 | N2 | C2 | training | high |
| TCGA-86-7953 | 997 | 0 | 69 | F | I | T1 | M0 | N0 | C1 | training | low |
| TCGA-86-7954 | 605 | 0 | 68 | F | I | T2 | M0 | N0 | C1 | training | low |
| TCGA-86-7955 | 1072 | 0 | 62 | M | I | T2 | M0 | N0 | C1 | training | high |
| TCGA-86-8055 | 124 | 1 | 79 | M | II | T2 | M0 | N1 | C1 | training | high |
| TCGA-86-8056 | 139 | 0 | 63 | F | III | T4 | M0 | N0 | C2 | training | low |
| TCGA-86-8073 | 740 | 0 | 58 | M | I | T2 | M0 | N0 | C1 | testing | low |
| TCGA-86-8074 | 24 | 0 | 62 | F | II | T1 | M0 | N1 | C1 | training | high |
| TCGA-86-8075 | 694 | 1 | 66 | F | I | T2 | M0 | N0 | C1 | testing | high |
| TCGA-86-8076 | 993 | 0 | 42 | M | I | T1 | M0 | N0 | C2 | testing | low |
| TCGA-86-8279 | 949 | 0 | 46 | M | II | T2 | M0 | N1 | C1 | training | high |
| TCGA-86-8280 | 701 | 0 | 54 | F | II | T2 | M0 | N0 | C2 | training | low |
| TCGA-86-8281 | 0 | 0 | 75 | M | I | T1 | M0 | NX | C2 | training | high |
| TCGA-86-8358 | 653 | 0 | 44 | M | I | T2 | M0 | N0 | C1 | testing | high |
| TCGA-86-8359 | 444 | 1 | 52 | M | III | T3 | M0 | N2 | C2 | testing | high |
| TCGA-86-8585 | 353 | 0 | 57 | M | I | T2 | M0 | N0 | C1 | training | low |
| TCGA-86-8668 | 423 | 0 | 61 | F | I | T1 | M0 | N0 | C2 | training | high |
| TCGA-86-8669 | 938 | 0 | 64 | M | I | T1 | M0 | N0 | C2 | training | low |
| TCGA-86-8671 | 839 | 0 | 72 | F | II | T2 | M0 | N1 | C2 | training | low |
| TCGA-86-8672 | 19 | 1 | 59 | M | II | T3 | M0 | N0 | C1 | training | high |
| TCGA-86-8673 | 862 | 0 | 61 | M | I | T2 | M0 | N0 | C1 | training | high |
| TCGA-86-8674 | 806 | 0 | 50 | M | II | T2 | M0 | N1 | C1 | training | high |
| TCGA-86-A456 | 896 | 0 | 78 | F | I | T1 | M0 | N0 | C2 | training | low |
| TCGA-86-A4D0 | 116 | 1 | 48 | M | II | T2 | M0 | N0 | C1 | training | high |
| TCGA-86-A4JF | 737 | 1 | 56 | M | II | T3 | M0 | N0 | C1 | testing | high |
| TCGA-86-A4P7 | 415 | 0 | 63 | F | I | T2 | M0 | N0 | C2 | training | low |
| TCGA-86-A4P8 | 805 | 0 | 59 | F | III | T1 | MX | N2 | C2 | training | low |
| TCGA-91-6828 | 323 | 0 | 70 | M | I | T1 | M0 | N0 | C2 | testing | low |
| TCGA-91-6829 | 1258 | 1 | 78 | M | I | T2 | MX | N0 | C1 | training | low |
| TCGA-91-6830 | 60 | 0 | 65 | F | II | T1 | MX | N1 | C2 | training | low |
| TCGA-91-6831 | 310 | 0 | 66 | M | I | T2 | MX | N0 | C1 | testing | high |
| TCGA-91-6835 | 79 | 0 | 81 | F | I | T1 | M0 | N0 | C2 | training | low |
| TCGA-91-6836 | 417 | 0 | 52 | F | I | T2 | MX | N0 | C1 | testing | high |
| TCGA-91-6840 | 372 | 0 | 59 | F | I | T1 | M0 | N0 | C1 | testing | low |
| TCGA-91-6847 | 842 | 0 | 62 | F | I | T2 | MX | N0 | C1 | testing | high |
| TCGA-91-6848 | 224 | 0 | 59 | M | III | T2 | MX | N2 | C1 | testing | high |
| TCGA-91-6849 | 35 | 0 | 75 | F | III | T2 | MX | N2 | C2 | training | low |
| TCGA-91-7771 | 492 | 0 | 62 | M | II | T3 | MX | N0 | C2 | training | low |
| TCGA-91-8496 | 505 | 0 | 63 | F | I | T2 | MX | NX | C2 | training | low |
| TCGA-91-8497 | 434 | 1 | 75 | F | I | T1 | MX | N0 | C2 | training | low |
| TCGA-91-8499 | 36 | 0 | 76 | F | I | T1 | MX | N0 | C1 | training | high |
| TCGA-91-A4BC | 44 | 0 | 59 | M | II | T2 | MX | N0 | C1 | training | low |
| TCGA-91-A4BD | 603 | 0 | 78 | M | II | T1 | MX | N1 | C2 | testing | low |
| TCGA-93-7347 | 683 | 0 | 76 | F | I | T1 | MX | N0 | C2 | testing | low |
| TCGA-93-7348 | 531 | 0 | 75 | F | I | T1 | MX | N0 | C2 | training | low |
| TCGA-93-8067 | 186 | 0 | 77 | M | I | T2 | MX | N0 | C1 | training | high |
| TCGA-93-A4JO | 33 | 1 | 70 | M | I | T1 | MX | N0 | C2 | training | low |
| TCGA-93-A4JP | 578 | 0 | 64 | M | IV | TX | M1 | NX | C2 | training | low |
| TCGA-95-7039 | 1272 | 0 | 54 | F | II | T3 | MX | N0 | C1 | testing | high |
| TCGA-95-7043 | 503 | 1 | 63 | F | I | T1 | MX | N0 | C1 | testing | high |
| TCGA-95-7562 | 87 | 1 | 71 | M | II | T2 | M0 | N1 | C1 | training | high |
| TCGA-95-7567 | 568 | 0 | 61 | M | II | T2 | M0 | N1 | C1 | training | low |
| TCGA-95-7944 | 377 | 0 | 71 | M | I | T1 | M0 | N0 | C1 | training | high |
| TCGA-95-7947 | 477 | 0 | 67 | M | I | T1 | M0 | N0 | C1 | testing | low |
| TCGA-95-7948 | 476 | 0 | 42 | F | I | T2 | M0 | N0 | C2 | testing | high |
| TCGA-95-8494 | 84 | 0 | 67 | M | II | T2 | M0 | N1 | C1 | training | high |
| TCGA-95-A4VK | 651 | 0 | 74 | F | III | T2 | M0 | N2 | C2 | training | high |
| TCGA-95-A4VN | 553 | 0 | 62 | F | II | T2 | M0 | N1 | C1 | training | high |
| TCGA-97-7547 | 1965 | 0 | 67 | F | I | T2 | MX | N0 | C2 | training | low |
| TCGA-97-7552 | 1932 | 0 | 70 | M | I | T2 | MX | N0 | C2 | training | low |
| TCGA-97-7553 | 1870 | 0 | 58 | F | I | T1 | MX | N0 | C2 | training | low |
| TCGA-97-7554 | 775 | 0 | 83 | F | III | T2 | M0 | N2 | C2 | testing | high |
| TCGA-97-7937 | 564 | 0 | 65 | M | I | T2 | MX | N0 | C1 | training | low |
| TCGA-97-7938 | 18 | 1 | 76 | F | I | T1 | MX | N0 | C2 | testing | low |
| TCGA-97-8171 | 568 | 0 | 81 | M | IV | T2 | M1 | N2 | C1 | training | high |
| TCGA-97-8174 | 164 | 1 | 67 | M | II | T2 | M0 | N0 | C2 | testing | low |
| TCGA-97-8175 | 551 | 0 | 55 | F | I | T2 | M0 | N0 | C1 | testing | high |
| TCGA-97-8177 | 499 | 0 | 59 | F | I | T2 | M0 | N0 | C2 | testing | low |
| TCGA-97-8552 | 626 | 0 | 55 | F | I | T1 | MX | N0 | C2 | testing | low |
| TCGA-97-A4LX | 614 | 0 | 81 | M | I | T2 | M0 | N0 | C2 | testing | low |
| TCGA-97-A4M0 | 652 | 0 | 60 | F | I | T2 | M0 | N0 | C2 | testing | low |
| TCGA-97-A4M1 | 601 | 0 | 52 | F | I | T1 | M0 | N0 | C2 | training | low |
| TCGA-97-A4M2 | 624 | 0 | 66 | M | I | T1 | M0 | N0 | C2 | testing | low |
| TCGA-97-A4M3 | 540 | 0 | 69 | F | I | T1 | M0 | N0 | C2 | training | low |
| TCGA-97-A4M5 | 634 | 0 | 83 | M | I | T1 | M0 | N0 | C2 | testing | low |
| TCGA-97-A4M6 | 568 | 0 | 45 | F | I | T1 | M0 | N0 | C2 | training | low |
| TCGA-97-A4M7 | 629 | 0 | 74 | M | I | T1 | M0 | N0 | C2 | training | low |
| TCGA-99-7458 | 747 | 0 | 74 | F | III | T4 | M0 | N0 | C2 | training | low |
| TCGA-99-8025 | 1060 | 0 | 72 | F | III | T3 | M0 | N2 | C1 | testing | low |
| TCGA-99-8028 | 1118 | 0 | 50 | F | I | T1 | M0 | N0 | C2 | training | low |
| TCGA-99-8032 | 44 | 0 | 61 | M | I | T1 | M0 | N0 | C1 | testing | low |
| TCGA-99-8033 | 656 | 1 | 74 | F | IV | TX | M1 | NX | C1 | training | high |
| TCGA-99-AA5R | 658 | 0 | 70 | F | I | T1 | M0 | N0 | C2 | training | low |
| TCGA-J2-8192 | 739 | 0 | 65 | F | II | T2 | MX | N1 | C2 | training | low |
| TCGA-J2-8194 | 724 | 0 | 69 | F | II | T3 | MX | N0 | C2 | training | low |
| TCGA-J2-A4AD | 550 | 1 | 61 | F | I | T1 | MX | N0 | C1 | training | high |
| TCGA-J2-A4AE | 1079 | 0 | 77 | F | I | T1 | MX | N0 | C2 | testing | low |
| TCGA-J2-A4AG | 988 | 0 | 66 | F | I | T1 | MX | N0 | C2 | training | low |
| TCGA-L4-A4E5 | 578 | 0 | 48 | F | I | T1 | M0 | N0 | C1 | training | high |
| TCGA-L4-A4E6 | 435 | 0 | 67 | M | I | T1 | M0 | N0 | C2 | training | low |
| TCGA-L9-A443 | 193 | 1 | 63 | F | I | T1 | MX | N0 | C2 | training | high |
| TCGA-L9-A444 | 307 | 0 | 60 | F | I | T1 | MX | N0 | C2 | testing | low |
| TCGA-L9-A50W | 442 | 1 | 75 | M | II | T1 | MX | N1 | C2 | training | high |
| TCGA-L9-A5IP | 58 | 1 | 40 | F | IV | T3 | M1 | N2 | C1 | testing | high |
| TCGA-L9-A743 | 664 | 0 | 56 | M | II | T2 | M0 | N1 | C2 | testing | low |
| TCGA-L9-A7SV | 565 | 0 | 69 | M | II | T2 | M0 | N1 | C1 | testing | low |
| TCGA-L9-A8F4 | 476 | 0 | 64 | F | I | T2 | MX | N0 | C1 | training | low |
| TCGA-MN-A4N1 | 827 | 0 | 60 | M | II | T2 | M0 | N1 | C1 | training | high |
| TCGA-MN-A4N4 | 1175 | 0 | 57 | M | I | T1 | M0 | N0 | C2 | training | high |
| TCGA-MN-A4N5 | 84 | 0 | 63 | M | I | T1 | M0 | N0 | C1 | training | low |
| TCGA-MP-A4SV | 2620 | 1 | 67 | M | I | T2 | M0 | N0 | C1 | training | low |
| TCGA-MP-A4SW | 1778 | 1 | 53 | M | II | T2 | M0 | N1 | C2 | testing | low |
| TCGA-MP-A4SY | 1501 | 1 | 61 | M | II | T2 | M0 | N1 | C1 | training | high |
| TCGA-MP-A4T4 | 2617 | 1 | 68 | F | II | T2 | M0 | N1 | C1 | training | low |
| TCGA-MP-A4T6 | 1790 | 1 | 76 | F | III | T1 | MX | N2 | C2 | training | low |
| TCGA-MP-A4T7 | 167 | 1 | 75 | F | IV | T2 | M1 | N0 | C1 | training | high |
| TCGA-MP-A4T8 | 161 | 1 | 68 | M | III | T2 | M0 | N2 | C1 | training | high |
| TCGA-MP-A4T9 | 1265 | 1 | 54 | F | III | T2 | MX | N2 | C2 | training | high |
| TCGA-MP-A4TA | 950 | 1 | 75 | F | I | T1 | M0 | N0 | C1 | training | high |
| TCGA-MP-A4TC | 74 | 1 | 77 | M | III | T1 | M0 | N2 | C1 | testing | high |
| TCGA-MP-A4TD | 307 | 1 | 71 | M | III | T2 | M0 | N2 | C2 | testing | low |
| TCGA-MP-A4TE | 896 | 1 | 56 | M | II | T2 | MX | N0 | C1 | testing | high |
| TCGA-MP-A4TF | 336 | 1 | 58 | F | II | T2 | M0 | N0 | C1 | training | high |
| TCGA-MP-A4TH | 741 | 0 | 70 | F | I | T1 | M0 | N0 | C2 | training | low |
| TCGA-MP-A4TI | 429 | 1 | 72 | M | II | T2 | M0 | N1 | C1 | testing | high |
| TCGA-MP-A4TK | 582 | 1 | 56 | F | II | T2 | MX | N1 | C1 | training | high |
| TCGA-MP-A5C7 | 2248 | 0 | 76 | F | I | T2 | M0 | N0 | C2 | testing | low |
| TCGA-NJ-A4YF | 2161 | 0 | 50 | F | I | T1 | M0 | N0 | C1 | training | low |
| TCGA-NJ-A4YG | 2261 | 0 | 65 | M | I | T2 | M0 | N0 | C2 | training | low |
| TCGA-NJ-A4YP | 50 | 0 | 52 | M | I | T2 | M0 | N0 | C1 | training | high |
| TCGA-NJ-A4YQ | 1432 | 0 | 69 | F | I | T1 | M0 | N0 | C2 | training | low |
| TCGA-NJ-A55R | 603 | 0 | 67 | M | I | T1 | MX | N0 | C2 | testing | high |
| TCGA-NJ-A7XG | 617 | 0 | 49 | M | III | T4 | M0 | N1 | C2 | training | high |
| TCGA-O1-A52J | 1798 | 1 | 74 | F | I | T1 | MX | N0 | C2 | training | low |
| TCGA-S2-AA1A | 513 | 0 | 68 | F | I | T1 | M0 | N0 | C2 | testing | low |

**Table S2.** 4061 TME-related genes.

| *A4GNT* | *AARD* | *ABCA13* | *ABCB4* | *ABCB5* | *ABCB9* | *ABCC1* | *ABCC11* |
| --- | --- | --- | --- | --- | --- | --- | --- |
| *ABCC4* | *ABCC5* | *ABCD1* | *ABCG2* | *ABHD11* | *ABHD16B* | *ABHD17A* | *ABHD2* |
| *ABHD3* | *ABHD5* | *ABI3BP* | *ABT1* | *ABTB1* | *ACACA* | *ACACB* | *ACAD8* |
| *ACADL* | *ACADM* | *ACAN* | *ACAP1* | *ACBD7* | *ACHE* | *ACKR2* | *ACOT9* |
| *ACP5* | *ACP6* | *ACRV1* | *ACSL1* | *ACSL3* | *ACSM1* | *ACSS3* | *ACTA2* |
| *ACTG1* | *ACTL10* | *ACTL6A* | *ACTL8* | *ACTN4* | *ACTR3* | *ACTR3B* | *ACVR1C* |
| *ACVRL1* | *ADAM12* | *ADAM15* | *ADAM21* | *ADAM23* | *ADAM28* | *ADAM33* | *ADAM7* |
| *ADAM8* | *ADAMDEC1* | *ADAMTS17* | *ADAMTS18* | *ADAMTS2* | *ADAMTS20* | *ADAMTS3* | *ADAMTSL1* |
| *ADAP2* | *ADAR* | *ADARB1* | *ADAT2* | *ADAT3* | *ADCK1* | *ADCK5* | *ADCY1* |
| *ADCY4* | *ADCY9* | *ADCYAP1* | *ADD2* | *ADGRB1* | *ADGRB2* | *ADGRE1* | *ADGRE2* |
| *ADGRE3* | *ADGRE5* | *ADGRF5* | *ADGRG1* | *ADGRG3* | *ADGRL4* | *ADIRF* | *ADORA1* |
| *ADRB2* | *ADRM1* | *AEN* | *AFAP1L1* | *AFAP1L2* | *AFF3* | *AGAP4* | *AGAP5* |
| *AGAP6* | *AGO2* | *AGPAT5* | *AHCYL1* | *AHCYL2* | *AHI1* | *AHNAK* | *AHR* |
| *AHSA1* | *AICDA* | *AIF1* | *AIFM2* | *AIM2* | *AK5* | *AKAP4* | *AKNA* |
| *AKR1B10* | *AKR1C1* | *AKR1C3* | *AKR1E2* | *AKT3* | *ALCAM* | *ALDH1A2* | *ALDH1B1* |
| *ALDH3A2* | *ALDH9A1* | *ALDOA* | *ALG11* | *ALG1L* | *ALG8* | *ALKBH3* | *ALKBH6* |
| *ALOX15* | *ALOX5* | *ALOX5AP* | *ALOXE3* | *ALPK3* | *ALPL* | *AMACR* | *AMDHD2* |
| *AMH* | *AMIGO3* | *AMOTL2* | *AMPD1* | *AMPD2* | *AMT* | *AMZ1* | *ANAPC11* |
| *ANAPC13* | *ANGEL1* | *ANGPT2* | *ANGPT4* | *ANGPTL2* | *ANK1* | *ANKFN1* | *ANKLE1* |
| *ANKRD10* | *ANKRD13D* | *ANKRD34B* | *ANKRD39* | *ANKRD55* | *ANKRD9* | *ANKS3* | *ANLN* |
| *ANO5* | *ANO7* | *ANO9* | *ANOS1* | *ANP32E* | *ANTXR1* | *ANXA4* | *ANXA5* |
| *AOC1* | *AOX1* | *AP1M1* | *AP1S1* | *AP2M1* | *AP2S1* | *AP3M2* | *AP3S1* |
| *APBA2* | *APBB2* | *APCDD1* | *APLN* | *APLP1* | *APLP2* | *APOBEC3A* | *APOBEC3G* |
| *APOC1* | *APOD* | *APOE* | *APOL3* | *APOL6* | *APOLD1* | *APOO* | *APP* |
| *AQP12A* | *AQP3* | *AQP9* | *ARFGEF3* | *ARHGAP10* | *ARHGAP11A* | *ARHGAP15* | *ARHGAP19* |
| *ARHGAP22* | *ARHGAP25* | *ARHGAP26* | *ARHGAP29* | *ARHGAP8* | *ARHGEF1* | *ARHGEF15* | *ARHGEF38* |
| *ARHGEF39* | *ARID3A* | *ARID4B* | *ARID5A* | *ARL1* | *ARL17A* | *ARL6* | *ARL9* |
| *ARMC12* | *ARMCX3* | *ARMT1* | *ARNT* | *ARNTL* | *ARRB1* | *ARSG* | *ARSH* |
| *ART5* | *ARX* | *ASAH2* | *ASB16* | *ASB2* | *ASB8* | *ASCL1* | *ASCL2* |
| *ASCL4* | *ASF1B* | *ASGR1* | *ASGR2* | *ASH2L* | *ASIC1* | *ASNS* | *ASPH* |
| *ASPHD1* | *ASPM* | *ASPRV1* | *ASPSCR1* | *ASRGL1* | *ATAD2* | *ATAD3B* | *ATAD5* |
| *ATF4* | *ATF7IP* | *ATG4D* | *ATG7* | *ATIC* | *ATL2* | *ATM* | *ATP10D* |
| *ATP13A1* | *ATP1A1* | *ATP1A3* | *ATP1B3* | *ATP5F1A* | *ATP5F1B* | *ATP5ME* | *ATP5MG* |
| *ATP6AP1* | *ATP6AP2* | *ATP6V0B* | *ATP6V0D2* | *ATP6V1A* | *ATP6V1B1* | *ATP6V1B2* | *ATP6V1C2* |
| *ATP6V1D* | *ATP6V1F* | *ATP6V1H* | *ATP7A* | *ATP8A2* | *ATP8B4* | *ATP9A* | *AUNIP* |
| *AURKA* | *AURKB* | *AVPR1B* | *AXIN2* | *AXL* | *AZU1* | *B2M* | *B3GALNT1* |
| *B3GAT1* | *B3GNT4* | *B4GALNT1* | *B4GALT3* | *B4GALT6* | *B4GALT7* | *BAALC* | *BACE2* |
| *BACH2* | *BAMBI* | *BANF1* | *BANK1* | *BARX1* | *BARX2* | *BASP1* | *BBC3* |
| *BBS12* | *BBS4* | *BCAP31* | *BCAT1* | *BCAT2* | *BCL11A* | *BCL11B* | *BCL2* |
| *BCL2A1* | *BCL2L1* | *BCL2L10* | *BCL2L12* | *BCL6* | *BCL6B* | *BCL7A* | *BEND4* |
| *BEND5* | *BEST1* | *BEX5* | *BFSP1* | *BHLHE40* | *BHLHE41* | *BIK* | *BIN2* |
| *BIRC2* | *BIRC3* | *BIRC5* | *BIRC7* | *BIVM* | *BLK* | *BLM* | *BLNK* |
| *BLOC1S3* | *BLVRB* | *BMI1* | *BMP2K* | *BMP6* | *BMPR1B* | *BMX* | *BOLA2* |
| *BOP1* | *BORA* | *BPGM* | *BPI* | *BPIFA2* | *BRAF* | *BRAP* | *BRCA1* |
| *BRCA2* | *BRDT* | *BRF2* | *BRINP1* | *BRINP2* | *BRIP1* | *BRIX1* | *BRMS1* |
| *BRSK2* | *BSN* | *BSND* | *BST1* | *BST2* | *BTG3* | *BTLA* | *BTN2A2* |
| *BTN3A2* | *BTNL8* | *BUB1* | *BUB1B* | *BUB3* | *BZW2* | *C10orf62* | *C10orf82* |
| *C11orf45* | *C11orf53* | *C11orf80* | *C12orf45* | *C12orf75* | *C17orf53* | *C17orf64* | *C17orf99* |
| *C19orf25* | *C19orf48* | *C19orf53* | *C19orf57* | *C1GALT1C1* | *C1orf112* | *C1orf159* | *C1orf162* |
| *C1orf54* | *C1orf61* | *C1QA* | *C1QB* | *C1QC* | *C1QL1* | *C1QL4* | *C1QTNF12* |
| *C1QTNF3* | *C1QTNF6* | *C1R* | *C1S* | *C20orf204* | *C21orf58* | *C2CD4D* | *C2CD6* |
| *C3* | *C3AR1* | *C3orf18* | *C5AR1* | *C5AR2* | *C5orf34* | *C6orf15* | *C6orf223* |
| *C6orf52* | *C7orf61* | *C8orf76* | *C9orf116* | *C9orf50* | *CA12* | *CA4* | *CA8* |
| *CA9* | *CABLES2* | *CABP4* | *CABP7* | *CABYR* | *CACNA1B* | *CACNA1D* | *CACNG4* |
| *CACNG7* | *CACYBP* | *CADM1* | *CADPS* | *CALB2* | *CALCA* | *CALCB* | *CALD1* |
| *CALHM1* | *CALHM3* | *CALN1* | *CALR* | *CAMK2N2* | *CAMKK2* | *CAMKV* | *CAMLG* |
| *CAMP* | *CAPG* | *CAPN12* | *CAPN13* | *CAPN3* | *CAPZA1* | *CARD11* | *CARD19* |
| *CARD8* | *CARD9* | *CASD1* | *CASKIN1* | *CASP3* | *CASP5* | *CASP8* | *CASQ1* |
| *CAT* | *CATSPERG* | *CAV1* | *CAVIN2* | *CBARP* | *CBLB* | *CBLN1* | *CBWD3* |
| *CBWD6* | *CBX2* | *CBX3* | *CBX6* | *CBX8* | *CC2D2B* | *CCDC102B* | *CCDC117* |
| *CCDC134* | *CCDC146* | *CCDC151* | *CCDC154* | *CCDC167* | *CCDC177* | *CCDC18* | *CCDC183* |
| *CCDC28B* | *CCDC34* | *CCDC57* | *CCDC74A* | *CCDC77* | *CCDC80* | *CCDC88A* | *CCDC91* |
| *CCKBR* | *CCL1* | *CCL13* | *CCL14* | *CCL17* | *CCL18* | *CCL19* | *CCL2* |
| *CCL20* | *CCL21* | *CCL22* | *CCL23* | *CCL26* | *CCL3L1* | *CCL4* | *CCL4L2* |
| *CCL5* | *CCL7* | *CCL8* | *CCN2* | *CCN4* | *CCNA1* | *CCNA2* | *CCNB1* |
| *CCNB2* | *CCNB3* | *CCND1* | *CCND2* | *CCNE1* | *CCNE2* | *CCNF* | *CCNI2* |
| *CCNO* | *CCR1* | *CCR10* | *CCR2* | *CCR3* | *CCR5* | *CCR6* | *CCR7* |
| *CCR9* | *CCT2* | *CCT3* | *CCT5* | *CCT6A* | *CCT6B* | *CCZ1B* | *CD101* |
| *CD109* | *CD14* | *CD151* | *CD160* | *CD163* | *CD180* | *CD19* | *CD1A* |
| *CD1B* | *CD1C* | *CD1D* | *CD1E* | *CD2* | *CD200* | *CD207* | *CD209* |
| *CD22* | *CD226* | *CD244* | *CD247* | *CD248* | *CD27* | *CD274* | *CD28* |
| *CD2BP2* | *CD300A* | *CD300E* | *CD300LB* | *CD300LF* | *CD302* | *CD320* | *CD33* |
| *CD34* | *CD36* | *CD37* | *CD38* | *CD3D* | *CD3E* | *CD3G* | *CD4* |
| *CD40* | *CD40LG* | *CD47* | *CD48* | *CD5* | *CD52* | *CD53* | *CD55* |
| *CD58* | *CD59* | *CD6* | *CD63* | *CD68* | *CD69* | *CD7* | *CD70* |
| *CD72* | *CD74* | *CD79A* | *CD79B* | *CD80* | *CD84* | *CD86* | *CD8A* |
| *CD8B* | *CD9* | *CD93* | *CD96* | *CDA* | *CDC14A* | *CDC20* | *CDC20B* |
| *CDC25A* | *CDC25B* | *CDC25C* | *CDC45* | *CDC5L* | *CDC6* | *CDC7* | *CDCA2* |
| *CDCA3* | *CDCA4* | *CDCA5* | *CDCA7* | *CDCA8* | *CDH1* | *CDH12* | *CDH19* |
| *CDH2* | *CDH24* | *CDH4* | *CDH5* | *CDH7* | *CDH8* | *CDHR1* | *CDIP1* |
| *CDK1* | *CDK10* | *CDK12* | *CDK16* | *CDK18* | *CDK2* | *CDK5* | *CDK5R1* |
| *CDK5RAP2* | *CDK5RAP3* | *CDK6* | *CDKAL1* | *CDKN2A* | *CDKN2AIP* | *CDKN3* | *CDT1* |
| *CEACAM19* | *CEACAM3* | *CEACAM6* | *CEACAM8* | *CEL* | *CELF3* | *CELF4* | *CELF5* |
| *CELSR3* | *CEMIP* | *CEMP1* | *CENPA* | *CENPE* | *CENPF* | *CENPH* | *CENPI* |
| *CENPK* | *CENPL* | *CENPM* | *CENPN* | *CENPO* | *CENPW* | *CEP131* | *CEP152* |
| *CEP55* | *CEP57* | *CEP68* | *CEP72* | *CEP78* | *CERS1* | *CERS4* | *CERS6* |
| *CETN3* | *CFHR3* | *CFL1* | *CFLAR* | *CFP* | *CFTR* | *CGB5* | *CH25H* |
| *CHAD* | *CHAF1A* | *CHAF1B* | *CHD1L* | *CHD5* | *CHEK1* | *CHEK2* | *CHI3L1* |
| *CHI3L2* | *CHIT1* | *CHL1* | *CHML* | *CHRM3* | *CHRM5* | *CHRNA5* | *CHRNB2* |
| *CHRNB4* | *CHST15* | *CHST2* | *CHST7* | *CHTF18* | *CIAO3* | *CIP2A* | *CIPC* |
| *CIT* | *CITED4* | *CKAP2* | *CKAP2L* | *CKAP4* | *CKAP5* | *CKS1B* | *CKS2* |
| *CLC* | *CLCN5* | *CLCN7* | *CLCNKB* | *CLDN11* | *CLDN20* | *CLDN25* | *CLDN5* |
| *CLDN9* | *CLEC10A* | *CLEC14A* | *CLEC16A* | *CLEC17A* | *CLEC1A* | *CLEC2B* | *CLEC2D* |
| *CLEC2L* | *CLEC4A* | *CLEC4C* | *CLEC5A* | *CLEC7A* | *CLEC9A* | *CLECL1* | *CLIC2* |
| *CLIC6* | *CLK4* | *CLN6* | *CLN8* | *CLNK* | *CLNS1A* | *CLPSL1* | *CLPSL2* |
| *CLPTM1L* | *CLSPN* | *CLTB* | *CLTC* | *CLUAP1* | *CMA1* | *CMKLR1* | *CMTM1* |
| *CMTM2* | *CNGB1* | *CNGB3* | *CNIH2* | *CNN1* | *CNPY3* | *CNPY4* | *CNR1* |
| *CNR2* | *CNTN3* | *CNTNAP2* | *CNTNAP4* | *CNTNAP5* | *COA6* | *COCH* | *COL11A2* |
| *COL12A1* | *COL14A1* | *COL1A1* | *COL1A2* | *COL23A1* | *COL26A1* | *COL2A1* | *COL3A1* |
| *COL4A1* | *COL4A4* | *COL5A2* | *COL5A3* | *COL6A1* | *COL6A2* | *COL6A3* | *COL8A2* |
| *COL9A1* | *COL9A2* | *COLCA2* | *COLEC12* | *COLQ* | *COMMD4* | *COMMD5* | *COPE* |
| *COPZ2* | *COQ9* | *CORO1A* | *CORO7* | *CORT* | *COTL1* | *COX6A1* | *COX6B1* |
| *COX7B* | *COX7B2* | *COX7C* | *CPA3* | *CPD* | *CPE* | *CPEB2* | *CPLX1* |
| *CPLX2* | *CPM* | *CPNE1* | *CPNE2* | *CPNE5* | *CPNE7* | *CPNE9* | *CPSF1* |
| *CPSF4L* | *CPT1B* | *CPVL* | *CPXM1* | *CPXM2* | *CR2* | *CRACR2B* | *CREB1* |
| *CREB3L1* | *CREB3L4* | *CREB5* | *CREBZF* | *CREG2* | *CREM* | *CRH* | *CRHR2* |
| *CRIP2* | *CRIP3* | *CRISP3* | *CRISPLD1* | *CRISPLD2* | *CRPPA* | *CRTAM* | *CRTC2* |
| *CRTC3* | *CRX* | *CRY2* | *CRYAB* | *CRYBA4* | *CRYBB1* | *CRYGN* | *CSAG1* |
| *CSAG2* | *CSAG3* | *CSE1L* | *CSF1* | *CSF1R* | *CSF2* | *CSF2RA* | *CSF3R* |
| *CSMD2* | *CSMD3* | *CSNK2A1* | *CSPG4* | *CSRP1* | *CSRP2* | *CST1* | *CST7* |
| *CSTA* | *CSTL1* | *CTAG1B* | *CTAG2* | *CTBS* | *CTCFL* | *CTLA4* | *CTNNA2* |
| *CTNND2* | *CTNS* | *CTPS2* | *CTRL* | *CTSE* | *CTSG* | *CTSK* | *CTSV* |
| *CTSW* | *CTSZ* | *CTTN* | *CTTNBP2* | *CTU1* | *CUZD1* | *CWF19L1* | *CXCL1* |
| *CXCL10* | *CXCL11* | *CXCL12* | *CXCL13* | *CXCL14* | *CXCL16* | *CXCL3* | *CXCL5* |
| *CXCL9* | *CXCR1* | *CXCR2* | *CXCR3* | *CXCR5* | *CXCR6* | *CXorf40A* | *CYB5B* |
| *CYBB* | *CYBRD1* | *CYLD* | *CYP11A1* | *CYP1B1* | *CYP26C1* | *CYP27A1* | *CYP27B1* |
| *CYP27C1* | *CYP2S1* | *CYP2W1* | *CYP4F2* | *CYP4F3* | *CYP4Z1* | *CYREN* | *CYSLTR2* |
| *CYTH1* | *DAB1* | *DAB2* | *DAB2IP* | *DACH1* | *DAGLB* | *DAP3* | *DAPK1* |
| *DAPK2* | *DAPP1* | *DARS* | *DAXX* | *DAZAP2* | *DBF4* | *DBNDD1* | *DBX1* |
| *DCAF10* | *DCAF13* | *DCAF15* | *DCAF4L1* | *DCAF4L2* | *DCAF8L2* | *DCLK2* | *DCLK3* |
| *DCN* | *DCST1* | *DCST2* | *DCSTAMP* | *DCUN1D5* | *DDIAS* | *DDIT3* | *DDR1* |
| *DDX11* | *DDX17* | *DDX39A* | *DDX3X* | *DDX3Y* | *DDX4* | *DDX41* | *DDX49* |
| *DDX53* | *DDX58* | *DDX60* | *DEDD* | *DEFA4* | *DEGS2* | *DENND1B* | *DENND1C* |
| *DENND5B* | *DEPDC1* | *DEPDC1B* | *DEPDC5* | *DEPP1* | *DGAT2* | *DGCR6* | *DGKA* |
| *DGKH* | *DGKI* | *DGKZ* | *DGLUCY* | *DHFR* | *DHRS11* | *DHRS7* | *DHX34* |
| *DHX58* | *DIAPH3* | *DIPK1B* | *DIPK2B* | *DIRAS1* | *DIRAS2* | *DISC1* | *DISP3* |
| *DLC1* | *DLEU7* | *DLGAP3* | *DLGAP5* | *DLK1* | *DLL4* | *DLX2* | *DLX4* |
| *DLX5* | *DLX6* | *DMBX1* | *DMC1* | *DMRT1* | *DMRT2* | *DMRTA2* | *DMRTC2* |
| *DMTN* | *DNA2* | *DNAAF3* | *DNAH11* | *DNAH14* | *DNAH17* | *DNAH2* | *DNAJB1* |
| *DNAJB13* | *DNAJC10* | *DNAJC12* | *DNAJC4* | *DNAJC9* | *DNASE1* | *DNASE1L2* | *DNASE2B* |
| *DND1* | *DNM1* | *DNMT1* | *DNMT3A* | *DNMT3B* | *DNTTIP1* | *DOC2A* | *DOC2B* |
| *DOCK10* | *DOCK2* | *DOCK5* | *DOCK6* | *DOCK9* | *DOK5* | *DONSON* | *DPCD* |
| *DPEP2* | *DPF1* | *DPM1* | *DPM3* | *DPP10* | *DPP4* | *DPP7* | *DPT* |
| *DPY19L1* | *DPYD* | *DPYSL5* | *DRAXIN* | *DRC7* | *DRICH1* | *DSC1* | *DSCC1* |
| *DSN1* | *DTD1* | *DTL* | *DTNB* | *DTX1* | *DTX4* | *DTYMK* | *DUS1L* |
| *DUS4L* | *DUSP12* | *DUSP14* | *DUSP15* | *DUSP2* | *DUSP23* | *DUSP5* | *DUSP6* |
| *DUT* | *DVL3* | *DYNC2LI1* | *DYNLT1* | *DYSF* | *DYTN* | *E2F1* | *E2F3* |
| *E2F5* | *E2F7* | *EAF2* | *EBF1* | *EBF2* | *EBI3* | *ECE1* | *ECE2* |
| *ECSCR* | *ECT2* | *EDA2R* | *EDAR* | *EDARADD* | *EDN1* | *EDRF1* | *EFCAB13* |
| *EFEMP1* | *EFEMP2* | *EFNA1* | *EFNA4* | *EFNA5* | *EFNB1* | *EGFL6* | *EGFL7* |
| *EGFR* | *EGLN3* | *EGR2* | *EGR3* | *EID1* | *EIF1* | *EIF1B* | *EIF2AK2* |
| *EIF2S2* | *EIF3H* | *EIF3L* | *EIF4A1* | *ELANE* | *ELAVL2* | *ELF1* | *ELFN2* |
| *ELK3* | *ELMO1* | *ELMO2* | *ELN* | *ELOA2* | *ELOA3* | *ELOB* | *ELOC* |
| *ELOVL7* | *ELP2* | *ELP3* | *EMCN* | *EME1* | *EME2* | *EMID1* | *EMP1* |
| *EMP3* | *EN1* | *EN2* | *ENC1* | *ENG* | *ENGASE* | *ENKD1* | *ENO2* |
| *ENPP1* | *ENPP3* | *ENTPD1* | *ENTPD6* | *ENY2* | *EOMES* | *EPB41* | *EPDR1* |
| *EPHA1* | *EPHA8* | *EPHB2* | *EPHB3* | *EPHB4* | *EPHB6* | *EPHX4* | *EPN2* |
| *EPOP* | *EPOR* | *EPS8* | *EPYC* | *EQTN* | *ERBB2* | *ERBB3* | *ERC2* |
| *ERCC6L* | *ERG* | *ERI1* | *ERICH5* | *ERP27* | *ESAM* | *ESCO2* | *ESM1* |
| *ESRRA* | *ETS1* | *ETV1* | *ETV3* | *ETV4* | *EVI2A* | *EVI2B* | *EVI5* |
| *EWSR1* | *EXO1* | *EXO5* | *EXOC2* | *EXOC6* | *EXOSC5* | *EXOSC6* | *EXOSC7* |
| *EXOSC8* | *EXOSC9* | *EXT2* | *EYA2* | *EYS* | *EZH2* | *EZR* | *F11R* |
| *F12* | *F13A1* | *F2RL2* | *F2RL3* | *F3* | *F8* | *F8A1* | *FAAP24* |
| *FABP1* | *FABP3* | *FABP4* | *FABP5* | *FABP6* | *FADD* | *FADS1* | *FAM102A* |
| *FAM110B* | *FAM111B* | *FAM117B* | *FAM124B* | *FAM133A* | *FAM135B* | *FAM136A* | *FAM153B* |
| *FAM166A* | *FAM170A* | *FAM171A2* | *FAM174B* | *FAM178B* | *FAM184B* | *FAM186B* | *FAM189A1* |
| *FAM189B* | *FAM222A* | *FAM234B* | *FAM24B* | *FAM3B* | *FAM3C* | *FAM43A* | *FAM47C* |
| *FAM49A* | *FAM50A* | *FAM71D* | *FAM71E1* | *FAM71E2* | *FAM71F2* | *FAM72A* | *FAM72B* |
| *FAM72D* | *FAM78B* | *FAM90A1* | *FAM9C* | *FANCA* | *FANCB* | *FANCD2* | *FANCE* |
| *FANCG* | *FANCI* | *FAP* | *FARP1* | *FASLG* | *FASN* | *FATE1* | *FBLL1* |
| *FBLN1* | *FBLN5* | *FBN1* | *FBN2* | *FBN3* | *FBXL16* | *FBXL17* | *FBXL20* |
| *FBXL6* | *FBXL8* | *FBXO17* | *FBXO27* | *FBXO30* | *FBXO41* | *FBXO43* | *FBXO47* |
| *FBXO5* | *FBXW9* | *FCAR* | *FCER1A* | *FCER1G* | *FCER2* | *FCGR1A* | *FCGR1B* |
| *FCGR2A* | *FCGR2B* | *FCGR3A* | *FCGR3B* | *FCMR* | *FCN1* | *FCRL1* | *FCRL2* |
| *FCRL3* | *FCRL4* | *FCRL5* | *FCRL6* | *FCRLA* | *FCRLB* | *FCSK* | *FDFT1* |
| *FDPS* | *FDX1* | *FEN1* | *FERMT1* | *FES* | *FEV* | *FEZ1* | *FFAR2* |
| *FGD4* | *FGD5* | *FGF12* | *FGF17* | *FGF18* | *FGF3* | *FGF7* | *FGF9* |
| *FGFBP2* | *FGFR4* | *FGGY* | *FGL2* | *FGR* | *FHL2* | *FIBIN* | *FIGN* |
| *FITM2* | *FKBP1B* | *FKBP4* | *FKBP6* | *FLAD1* | *FLI1* | *FLT3LG* | *FLT4* |
| *FLVCR2* | *FN1* | *FNIP2* | *FOLH1* | *FOSB* | *FOSL1* | *FOXA1* | *FOXD1* |
| *FOXD4* | *FOXD4L1* | *FOXD4L4* | *FOXD4L5* | *FOXD4L6* | *FOXE3* | *FOXG1* | *FOXH1* |
| *FOXI1* | *FOXI2* | *FOXI3* | *FOXJ1* | *FOXM1* | *FOXN4* | *FOXP1* | *FOXP3* |
| *FOXRED2* | *FPGS* | *FPR1* | *FPR2* | *FPR3* | *FRAT2* | *FRK* | *FRMD3* |
| *FRMD4A* | *FRMD8* | *FRMPD2* | *FRMPD4* | *FRY* | *FSCN1* | *FSD1* | *FSIP1* |
| *FSTL1* | *FSTL5* | *FTL* | *FTSJ3* | *FUCA1* | *FURIN* | *FUT1* | *FUT4* |
| *FUT5* | *FUT8* | *FUT9* | *FXYD3* | *FXYD6* | *FYB1* | *FYN* | *FZD2* |
| *FZD3* | *FZD4* | *FZD6* | *FZD8* | *FZR1* | *G0S2* | *G6PD* | *GAB3* |
| *GABARAP* | *GABPB1* | *GABRA3* | *GABRB2* | *GABRD* | *GABRG3* | *GABRQ* | *GABRR1* |
| *GAD1* | *GADD45A* | *GADD45GIP1* | *GAGE12J* | *GAGE2A* | *GAL3ST1* | *GAL3ST4* | *GALC* |
| *GALNT11* | *GALNT14* | *GALNT16* | *GALNT17* | *GALNTL6* | *GALR1* | *GALR3* | *GAPDH* |
| *GAS2L3* | *GASK1B* | *GATA2* | *GATA3* | *GATD1* | *GATM* | *GBA* | *GBP2* |
| *GBP3* | *GBX2* | *GCK* | *GCNT1* | *GCSH* | *GDA* | *GDAP1* | *GDE1* |
| *GDF1* | *GDF15* | *GDF6* | *GDF9* | *GDPD1* | *GDPD4* | *GDPD5* | *GEMIN2* |
| *GEMIN6* | *GFI1* | *GFM1* | *GFOD1* | *GFOD2* | *GFPT2* | *GFRA1* | *GFRA3* |
| *GGCT* | *GGH* | *GGT1* | *GGT5* | *GIMAP1-* | *GIMAP5* | *GINS1* | *GINS2* |
| *GINS4* | *GIPR* | *GJA3* | *GJB1* | *GJB4* | *GJB7* | *GJD2* | *GK* |
| *GLB1L3* | *GLDC* | *GLDN* | *GLIPR1* | *GLOD4* | *GLP1R* | *GLRB* | *GLS2* |
| *GLT8D2* | *GLUD1* | *GLUL* | *GLYATL2* | *GM2A* | *GMDS* | *GMNN* | *GMPS* |
| *GNAI1* | *GNAS* | *GNAT1* | *GNG11* | *GNG7* | *GNLY* | *GOLGA6L1* | *GOLGA7B* |
| *GOLGA8A* | *GOLGA8B* | *GOLM1* | *GOLT1B* | *GPAA1* | *GPAT4* | *GPATCH2* | *GPBP1* |
| *GPC1* | *GPC2* | *GPC3* | *GPC4* | *GPI* | *GPM6B* | *GPNMB* | *GPR1* |
| *GPR137C* | *GPR139* | *GPR143* | *GPR146* | *GPR15* | *GPR152* | *GPR153* | *GPR171* |
| *GPR174* | *GPR179* | *GPR18* | *GPR183* | *GPR19* | *GPR25* | *GPR26* | *GPR27* |
| *GPR4* | *GPR45* | *GPR61* | *GPR65* | *GPR89B* | *GPRC5B* | *GPRIN1* | *GPSM1* |
| *GPT2* | *GPX2* | *GRAP2* | *GRB10* | *GRB2* | *GRB7* | *GREM1* | *GREM2* |
| *GRID2* | *GRIN2B* | *GRIN2D* | *GRIN3A* | *GRINA* | *GRK7* | *GRM1* | *GRM2* |
| *GRM5* | *GRM8* | *GRPR* | *GSG1* | *GSS* | *GSTA4* | *GSTCD* | *GTF2F2* |
| *GTF3A* | *GTF3C5* | *GTSE1* | *GTSF1* | *GUCA1A* | *GUCY2D* | *GUSB* | *GYPE* |
| *GZMA* | *GZMB* | *GZMH* | *GZMK* | *GZMM* | *H2AFB1* | *H2AFX* | *H2AFY* |
| *H2AFY2* | *H2BFM* | *H3F3A* | *HADHA* | *HAGHL* | *HAL* | *HAPLN3* | *HASPIN* |
| *HAUS3* | *HAUS6* | *HAUS8* | *HAVCR1* | *HAVCR2* | *HBEGF* | *HCAR1* | *HCFC1R1* |
| *HCK* | *HCLS1* | *HCN2* | *HCN3* | *HCN4* | *HDC* | *HEATR4* | *HEATR6* |
| *HECW2* | *HELLS* | *HENMT1* | *HEPACAM2* | *HEPHL1* | *HERC5* | *HERC6* | *HES1* |
| *HES4* | *HES6* | *HES7* | *HESX1* | *HGH1* | *HHEX* | *HHIP* | *HHIPL2* |
| *HIBCH* | *HIC1* | *HIC2* | *HIGD1A* | *HIGD2A* | *HIGD2B* | *HILPDA* | *HIST1H1B* |
| *HIST1H1C* | *HIST1H1D* | *HIST1H2AD* | *HIST1H2AE* | *HIST1H2AG* | *HIST1H2AJ* | *HIST1H2AL* | *HIST1H2AM* |
| *HIST1H2BB* | *HIST1H2BC* | *HIST1H2BF* | *HIST1H2BG* | *HIST1H2BH* | *HIST1H2BJ* | *HIST1H2BL* | *HIST1H2BN* |
| *HIST1H2BO* | *HIST1H3A* | *HIST1H3F* | *HIST1H3H* | *HIST1H3J* | *HIST1H4B* | *HIST1H4C* | *HIST1H4D* |
| *HIST1H4E* | *HIST1H4H* | *HIST1H4I* | *HIST1H4J* | *HIST2H2AC* | *HIST2H3C* | *HIST2H3D* | *HIST3H2A* |
| *HIST3H2BB* | *HIST4H4* | *HIVEP2* | *HJURP* | *HK3* | *HLA-A* | *HLA-B* | *HLA-C* |
| *HLA-DMA* | *HLA-DMB* | *HLA-DOB* | *HLA-DPA1* | *HLA-DPB1* | *HLA-DQA1* | *HLA-DQA2* | *HLA-DQB1* |
| *HLA-DRA* | *HLA-DRB1* | *HLA-G* | *HMG20B* | *HMGA1* | *HMGB2* | *HMGB3* | *HMMR* |
| *HMSD* | *HNF1B* | *HNMT* | *HNRNPD* | *HNRNPH1* | *HNRNPLL* | *HNRNPM* | *HOMER2* |
| *HORMAD1* | *HOXA1* | *HOXA2* | *HOXA3* | *HOXA4* | *HOXA9* | *HOXB13* | *HOXB5* |
| *HOXB9* | *HOXC12* | *HOXC13* | *HOXD1* | *HPCA* | *HPDL* | *HPGD* | *HPGDS* |
| *HPS4* | *HPSE* | *HRH1* | *HRH2* | *HRH4* | *HRK* | *HS3ST2* | *HS6ST3* |
| *HSD11B1* | *HSD11B2* | *HSD17B11* | *HSD17B3* | *HSF2BP* | *HSF4* | *HSPA1A* | *HSPA1B* |
| *HSPA4* | *HSPA6* | *HSPB1* | *HSPB6* | *HSPB7* | *HSPBP1* | *HSPD1* | *HSPG2* |
| *HTR2B* | *HTR2C* | *HUNK* | *HUS1B* | *HVCN1* | *HYAL2* | *HYAL3* | *HYDIN* |
| *HYLS1* | *IARS* | *IBSP* | *ICA1* | *ICAM2* | *ICAM3* | *ICOS* | *ICOSLG* |
| *ID1* | *ID3* | *IDH3A* | *IDO1* | *IFI16* | *IFI30* | *IFI35* | *IFI44* |
| *IFI44L* | *IFI6* | *IFIH1* | *IFIT1* | *IFIT2* | *IFIT3* | *IFIT5* | *IFITM2* |
| *IFNA10* | *IFNG* | *IFRD1* | *IGDCC3* | *IGF1* | *IGF1R* | *IGF2* | *IGF2BP1* |
| *IGF2BP2* | *IGF2BP3* | *IGFBP3* | *IGFBP5* | *IGFBP6* | *IGFLR1* | *IGHMBP2* | *IGLL5* |
| *IGLON5* | *IGSF6* | *IGSF8* | *IKBIP* | *IKBKG* | *IKZF1* | *IL10RB* | *IL12A* |
| *IL12B* | *IL12RB2* | *IL16* | *IL17A* | *IL17REL* | *IL18* | *IL18R1* | *IL18RAP* |
| *IL1A* | *IL1B* | *IL1RAPL1* | *IL1RL1* | *IL1RN* | *IL21* | *IL21R* | *IL22* |
| *IL26* | *IL2RA* | *IL2RB* | *IL3* | *IL32* | *IL34* | *IL37* | *IL3RA* |
| *IL4* | *IL4I1* | *IL4R* | *IL5* | *IL5RA* | *IL6ST* | *IL7* | *IL7R* |
| *IL9* | *IL9R* | *ILF2* | *IMP3* | *IMPDH1* | *IMPDH2* | *IMPG1* | *INA* |
| *ING3* | *INKA2* | *INO80C* | *INPP4B* | *INPP5F* | *INPPL1* | *INSM1* | *INSR* |
| *INSYN1* | *INSYN2A* | *INTS4* | *INTS7* | *INTS8* | *IPCEF1* | *IPO11* | *IQCB1* |
| *IQGAP3* | *IRAK3* | *IRF1* | *IRF4* | *IRF7* | *IRF8* | *IRF9* | *IRGC* |
| *IRX3* | *IRX5* | *IRX6* | *ISCU* | *ISG15* | *ISG20* | *ISL2* | *ISLR* |
| *ISM2* | *ITGA11* | *ITGA2B* | *ITGA4* | *ITGA9* | *ITGAL* | *ITGAM* | *ITGAX* |
| *ITGB1* | *ITGB2* | *ITGB4* | *ITGB7* | *ITGB8* | *ITIH2* | *ITIH6* | *ITK* |
| *ITM2A* | *ITPRIPL2* | *IZUMO2* | *JAG1* | *JAG2* | *JCHAIN* | *JMJD4* | *JPH3* |
| *JTB* | *JUP* | *KAAG1* | *KANK2* | *KAT6A* | *KAT6B* | *KATNAL2* | *KAZALD1* |
| *KBTBD11* | *KBTBD12* | *KCNA3* | *KCNB2* | *KCNE3* | *KCNF1* | *KCNG1* | *KCNG2* |
| *KCNG3* | *KCNH1* | *KCNH2* | *KCNH8* | *KCNIP1* | *KCNJ11* | *KCNJ15* | *KCNJ3* |
| *KCNK5* | *KCNK9* | *KCNMB4* | *KCNN1* | *KCNN2* | *KCNQ3* | *KCNT2* | *KCNU1* |
| *KCNV1* | *KCP* | *KCTD19* | *KCTD6* | *KDM1A* | *KDM4C* | *KDM6B* | *KDR* |
| *KEL* | *KHDC1L* | *KHDC4* | *KIAA0319* | *KIAA1257* | *KIAA1324* | *KIAA1324L* | *KIAA1328* |
| *KIAA1549L* | *KIF11* | *KIF13A* | *KIF13B* | *KIF14* | *KIF15* | *KIF18A* | *KIF18B* |
| *KIF1A* | *KIF20A* | *KIF21A* | *KIF23* | *KIF24* | *KIF26B* | *KIF2C* | *KIF3C* |
| *KIF4A* | *KIF4B* | *KIFC1* | *KIFC2* | *KIR2DL1* | *KIR2DL3* | *KIR2DL4* | *KIR3DL1* |
| *KIR3DL2* | *KIRREL1* | *KIRREL3* | *KISS1* | *KISS1R* | *KIT* | *KLF1* | *KLF12* |
| *KLF5* | *KLF9* | *KLHDC2* | *KLHDC4* | *KLHL14* | *KLHL17* | *KLHL21* | *KLHL26* |
| *KLHL3* | *KLHL35* | *KLHL4* | *KLHL7* | *KLK1* | *KLK15* | *KLK2* | *KLK5* |
| *KLRB1* | *KLRC1* | *KLRC3* | *KLRC4* | *KLRC4-* | *KLRD1* | *KLRF1* | *KLRG1* |
| *KLRK1* | *KMT2A* | *KNL1* | *KNOP1* | *KNTC1* | *KPNA2* | *KPNA7* | *KRBA1* |
| *KREMEN2* | *KRT23* | *KRT74* | *KRT80* | *KRTAP1-5* | *KRTCAP2* | *KSR1* | *KYNU* |
| *L1CAM* | *LACTB2* | *LAG3* | *LAGE3* | *LAIR1* | *LAIR2* | *LAMA5* | *LAMB1* |
| *LAMB4* | *LAMC1* | *LAMP2* | *LAMP3* | *LAMP5* | *LAMTOR2* | *LAP3* | *LAPTM4B* |
| *LAPTM5* | *LARS2* | *LAT* | *LBH* | *LBR* | *LCE1B* | *LCE1C* | *LCK* |
| *LCN10* | *LCN12* | *LCORL* | *LCP2* | *LCT* | *LCTL* | *LDB2* | *LDB3* |
| *LDHA* | *LDLRAD3* | *LEF1* | *LEPROTL1* | *LFNG* | *LGALS1* | *LGALS9* | *LGMN* |
| *LGSN* | *LHCGR* | *LHFPL4* | *LHX9* | *LIG1* | *LILRA1* | *LILRA2* | *LILRA4* |
| *LILRA5* | *LILRA6* | *LILRB2* | *LILRB4* | *LILRB5* | *LIMA1* | *LIME1* | *LIMK1* |
| *LIMS1* | *LIMS3* | *LIN28A* | *LIN28B* | *LIN7A* | *LIN9* | *LINGO1* | *LINGO3* |
| *LIPA* | *LITAF* | *LKAAEAR1* | *LMAN2* | *LMAN2L* | *LMF2* | *LMNB1* | *LMNB2* |
| *LNP1* | *LONRF1* | *LOX* | *LOXL4* | *LPAR1* | *LPAR2* | *LPAR5* | *LPGAT1* |
| *LPIN3* | *LRCH4* | *LRFN1* | *LRFN4* | *LRFN5* | *LRG1* | *LRGUK* | *LRIG1* |
| *LRIT3* | *LRMP* | *LRP1* | *LRP4* | *LRP5L* | *LRP8* | *LRRC14B* | *LRRC25* |
| *LRRC26* | *LRRC4* | *LRRC42* | *LRRC45* | *LRRC52* | *LRRC56* | *LRRIQ4* | *LRRN1* |
| *LRRN3* | *LRRN4CL* | *LRRTM1* | *LSG1* | *LSM1* | *LSM2* | *LSM4* | *LSM7* |
| *LSP1* | *LST1* | *LTA* | *LTB* | *LTBP2* | *LTC4S* | *LTK* | *LTO1* |
| *LUC7L3* | *LUM* | *LUZP2* | *LY6G5C* | *LY86* | *LY9* | *LY96* | *LYG1* |
| *LYG2* | *LYPD6B* | *LYST* | *LYVE1* | *LYZ* | *M1AP* | *MAB21L3* | *MACC1* |
| *MAD2L1* | *MAD2L2* | *MAFA* | *MAFB* | *MAGEA10* | *MAGEA11* | *MAGEA12* | *MAGEA2* |
| *MAGEA3* | *MAGEA6* | *MAGEC1* | *MAGEC2* | *MAGEC3* | *MAGED1* | *MAGED2* | *MAGED4* |
| *MAGOHB* | *MAJIN* | *MAK* | *MAL* | *MALL* | *MAN1A1* | *MAN1B1* | *MANEA* |
| *MANEAL* | *MAOB* | *MAP3K1* | *MAP3K13* | *MAP4K1* | *MAP4K2* | *MAP7D2* | *MAP9* |
| *MAPK15* | *MAPK7* | *MAPK8IP1* | *MAPK8IP2* | *MAPRE3* | *MARC1* | *MARCKSL1* | *MARCO* |
| *MASP1* | *MAST1* | *MASTL* | *MATN2* | *MB* | *MBL2* | *MBLAC1* | *MBOAT7* |
| *MBP* | *MC1R* | *MCCC2* | *MCF2L2* | *MCM10* | *MCM2* | *MCM3* | *MCM3AP* |
| *MCM4* | *MCM5* | *MCM6* | *MCM7* | *MCM8* | *MCOLN3* | *MDGA2* | *MDH1* |
| *MDK* | *MDM2* | *ME1* | *ME3* | *MED1* | *MED10* | *MED12L* | *MED13* |
| *MED7* | *MEF2C* | *MEFV* | *MEGF8* | *MEGF9* | *MEIOC* | *MELK* | *MELTF* |
| *MEP1A* | *MESP1* | *MESP2* | *MEST* | *MET* | *METRNL* | *METTL11B* | *METTL21A* |
| *METTL26* | *METTL27* | *METTL9* | *MEX3A* | *MFAP3L* | *MFAP4* | *MFAP5* | *MFGE8* |
| *MFSD10* | *MFSD2B* | *MFSD3* | *MFSD4B* | *MGAM* | *MGAT5B* | *MGST1* | *MIA* |
| *MICAL2* | *MICAL3* | *MICB* | *MICOS13* | *MIEN1* | *MIF* | *MIF4GD* | *MINPP1* |
| *MIPEP* | *MIS18A* | *MITF* | *MKI67* | *MKRN3* | *MLANA* | *MLPH* | *MMD* |
| *MME* | *MMEL1* | *MMP12* | *MMP15* | *MMP16* | *MMP2* | *MMP24* | *MMP25* |
| *MMP9* | *MMRN1* | *MMRN2* | *MMS22L* | *MND1* | *MNDA* | *MOB1B* | *MOCOS* |
| *MOCS3* | *MON1B* | *MPC2* | *MPG* | *MPL* | *MPO* | *MPP1* | *MPP6* |
| *MPV17* | *MPV17L2* | *MPZL1* | *MRC1* | *MRC2* | *MRGBP* | *MRGPRE* | *MROH7* |
| *MRPL24* | *MRPL4* | *MRPL47* | *MRPL52* | *MRPL55* | *MRPS17* | *MRPS21* | *MRPS30* |
| *MS4A1* | *MS4A2* | *MS4A3* | *MS4A4A* | *MS4A6A* | *MS4A7* | *MSANTD3* | *MSC* |
| *MSH4* | *MSH5* | *MSI1* | *MSL1* | *MSLNL* | *MSMP* | *MSR1* | *MSRB1* |
| *MTBP* | *MTCP1* | *MTERF3* | *MTFR2* | *MTHFD1L* | *MTHFD2* | *MTMR7* | *MTNR1B* |
| *MTSS2* | *MTUS1* | *MTX1* | *MUC20* | *MX1* | *MX2* | *MXD1* | *MXD3* |
| *MXRA8* | *MYB* | *MYBL1* | *MYBL2* | *MYBPC3* | *MYBPHL* | *MYC* | *MYCBP* |
| *MYCL* | *MYCN* | *MYCT1* | *MYEOV* | *MYH4* | *MYH7B* | *MYL9* | *MYO10* |
| *MYO15B* | *MYO1F* | *MYO1G* | *MYO1H* | *MYO3A* | *MYO7A* | *MYPOP* | *MYRIP* |
| *MYT1* | *MZB1* | *MZT1* | *MZT2A* | *NAA10* | *NAA20* | *NAA38* | *NAAA* |
| *NAALADL1* | *NAALADL2* | *NAB1* | *NACC1* | *NADSYN1* | *NANOS3* | *NARF* | *NAT1* |
| *NAT10* | *NATD1* | *NAV1* | *NAV3* | *NAXE* | *NBPF15* | *NBPF4* | *NBPF6* |
| *NCALD* | *NCAM2* | *NCAPD2* | *NCAPG* | *NCAPG2* | *NCAPH* | *NCBP2* | *NCF1* |
| *NCF2* | *NCKAP1L* | *NCOA4* | *NCR1* | *NCR3* | *NDC80* | *NDFIP2* | *NDRG1* |
| *NDUFA12* | *NDUFA6* | *NDUFA7* | *NDUFA8* | *NDUFAF6* | *NDUFB9* | *NDUFS6* | *NDUFS8* |
| *NECTIN2* | *NECTIN3* | *NEDD4L* | *NEFL* | *NEIL3* | *NEK2* | *NEK3* | *NEK5* |
| *NELFCD* | *NEMP2* | *NETO2* | *NEU1* | *NEURL2* | *NEUROD1* | *NEUROD2* | *NF2* |
| *NFAT5* | *NFATC1* | *NFATC2IP* | *NFATC3* | *NFATC4* | *NFE2* | *NFE2L2* | *NFE2L3* |
| *NFE4* | *NFKBIA* | *NHLH1* | *NHLRC1* | *NHSL2* | *NIBAN3* | *NID2* | *NINJ1* |
| *NIPSNAP3A* | *NIPSNAP3B* | *NKAIN2* | *NKAIN3* | *NKD1* | *NKG7* | *NLGN1* | *NLRP11* |
| *NLRP12* | *NLRP2* | *NLRP3* | *NLRP8* | *NMB* | *NMBR* | *NME3* | *NME8* |
| *NMRAL1* | *NMUR2* | *NNT* | *NOD2* | *NODAL* | *NOL11* | *NOL3* | *NOL4* |
| *NOL7* | *NOMO2* | *NOMO3* | *NOP10* | *NOP58* | *NOS2* | *NOS3* | *NOTUM* |
| *NOX1* | *NOX3* | *NOX4* | *NOXA1* | *NOXO1* | *NPAS1* | *NPAS3* | *NPB* |
| *NPDC1* | *NPEPL1* | *NPFFR1* | *NPIPB15* | *NPIPB3* | *NPL* | *NPPA* | *NPR1* |
| *NPTX2* | *NQO1* | *NR0B1* | *NR2C2AP* | *NR2E1* | *NR2E3* | *NR2F6* | *NR4A2* |
| *NR4A3* | *NR5A1* | *NRBP2* | *NRG1* | *NRG2* | *NRG4* | *NRIP3* | *NRP2* |
| *NRTN* | *NRXN3* | *NSD3* | *NSMCE1* | *NSMCE2* | *NSMF* | *NSUN5* | *NSUN7* |
| *NT5C1B* | *NT5DC2* | *NT5DC3* | *NT5M* | *NTN1* | *NTN3* | *NTRK1* | *NTS* |
| *NUCB2* | *NUDCD1* | *NUDT1* | *NUDT16* | *NUDT8* | *NUDT9* | *NUF2* | *NUP107* |
| *NUP155* | *NUP210* | *NUP210L* | *NUP37* | *NUP85* | *NUSAP1* | *NUTM1* | *NVL* |
| *NXF2* | *NXF5* | *NXPH4* | *OAS1* | *OAS2* | *OAS3* | *OCIAD2* | *OCSTAMP* |
| *ODC1* | *OFD1* | *OGA* | *OGDHL* | *OGT* | *OIP5* | *OLFML2B* | *OLFML3* |
| *OLIG1* | *OLR1* | *ONECUT2* | *OPA1* | *OPCML* | *OPRD1* | *OR1J2* | *OR1J4* |
| *OR1L8* | *OR2B6* | *OR51E1* | *OR52I1* | *OR5B12* | *OR6B2* | *ORC1* | *ORC6* |
| *ORM1* | *OSBP2* | *OSBPL10* | *OSBPL1A* | *OSBPL3* | *OSER1* | *OSM* | *OTP* |
| *OTUD7A* | *OTX2* | *OXCT1* | *OXGR1* | *P2RX1* | *P2RX5* | *P2RY10* | *P2RY13* |
| *P2RY14* | *P2RY2* | *P4HA1* | *P4HB* | *PABPC1* | *PABPC1L* | *PABPC1L2A* | *PABPC1L2B* |
| *PACRG* | *PACSIN1* | *PADI3* | *PADI4* | *PAFAH1B3* | *PAK5* | *PALLD* | *PALMD* |
| *PAM* | *PAMR1* | *PANX2* | *PAPSS2* | *PAQR4* | *PAQR5* | *PAQR6* | *PARK7* |
| *PARPBP* | *PARVA* | *PARVB* | *PASK* | *PAX3* | *PAX5* | *PAX6* | *PAX7* |
| *PAXX* | *PBK* | *PBRM1* | *PBX3* | *PBX4* | *PBXIP1* | *PCDH18* | *PCDH19* |
| *PCDHA1* | *PCDHA11* | *PCDHA2* | *PCDHA4* | *PCDHA5* | *PCDHA7* | *PCDHA8* | *PCDHB11* |
| *PCDHB16* | *PCDHB2* | *PCDHB3* | *PCDHB5* | *PCDHB8* | *PCDHGA8* | *PCDHGB1* | *PCDHGC4* |
| *PCGF1* | *PCGF2* | *PCLAF* | *PCM1* | *PCNA* | *PCNX1* | *PCNX2* | *PCNX4* |
| *PCOLCE* | *PCOLCE2* | *PCP2* | *PCSK2* | *PCSK5* | *PCSK9* | *PDCD1* | *PDCD1LG2* |
| *PDCD2L* | *PDCD5* | *PDE1C* | *PDE2A* | *PDE4B* | *PDE6C* | *PDE7B* | *PDF* |
| *PDGFA* | *PDGFRA* | *PDGFRB* | *PDGFRL* | *PDIA4* | *PDK1* | *PDK3* | *PDLIM4* |
| *PDP1* | *PDPN* | *PDRG1* | *PDX1* | *PDXK* | *PDZD11* | *PEAR1* | *PECAM1* |
| *PEG10* | *PELO* | *PEX10* | *PEX5L* | *PF4* | *PFDN2* | *PFKFB4* | *PFKM* |
| *PFKP* | *PFN2* | *PGAM4* | *PGC* | *PGF* | *PGGHG* | *PGK2* | *PGLYRP1* |
| *PGP* | *PGPEP1L* | *PHC3* | *PHEX* | *PHF24* | *PHF7* | *PHKA1* | *PHLDA1* |
| *PHOSPHO1* | *PHRF1* | *PHYHIPL* | *PI3* | *PIAS3* | *PICALM* | *PIEZO1* | *PIF1* |
| *PIGL* | *PIGO* | *PIGU* | *PIGX* | *PIK3CD* | *PIK3IP1* | *PIK3R2* | *PIK3R3* |
| *PILRA* | *PILRB* | *PIM2* | *PIM3* | *PIMREG* | *PIP4P1* | *PIP5K1A* | *PIP5KL1* |
| *PIR* | *PISD* | *PIWIL2* | *PIWIL3* | *PIWIL4* | *PJA2* | *PJVK* | *PKD2L2* |
| *PKM* | *PKMYT1* | *PKP4* | *PLA1A* | *PLA2G2D* | *PLA2G4A* | *PLA2G4C* | *PLA2G4F* |
| *PLA2G7* | *PLAC8L1* | *PLAC9* | *PLAG1* | *PLAGL2* | *PLAU* | *PLAUR* | *PLBD1* |
| *PLCB4* | *PLCG2* | *PLCH2* | *PLCL2* | *PLD4* | *PLD5* | *PLEK* | *PLEK2* |
| *PLEKHA4* | *PLEKHB1* | *PLEKHB2* | *PLEKHF1* | *PLEKHF2* | *PLEKHG3* | *PLEKHG4* | *PLEKHH2* |
| *PLEKHO2* | *PLIN2* | *PLIN5* | *PLK1* | *PLK4* | *PLLP* | *PLOD3* | *PLP1* |
| *PLPP2* | *PLPP5* | *PLSCR1* | *PLVAP* | *PLXNA1* | *PLXNA2* | *PLXNA3* | *PLXNC1* |
| *PM20D1* | *PMCH* | *PMEL* | *PMEPA1* | *PMFBP1* | *PMPCB* | *PNCK* | *PNKP* |
| *PNLDC1* | *PNMA2* | *PNMA3* | *PNOC* | *PNPLA6* | *PNPLA7* | *PNPLA8* | *POC1A* |
| *POC1B* | *PODN* | *PODXL* | *PODXL2* | *POFUT1* | *POGK* | *POGLUT2* | *POLA2* |
| *POLD1* | *POLE2* | *POLN* | *POLQ* | *POLR2H* | *POLR2I* | *POLR2J2* | *POLR3K* |
| *POMP* | *POP4* | *POP5* | *POU2AF1* | *POU2F2* | *POU3F2* | *POU4F1* | *POU4F3* |
| *POU5F1B* | *POU6F2* | *PPA1* | *PPARG* | *PPARGC1A* | *PPAT* | *PPBP* | *PPEF1* |
| *PPFIA1* | *PPFIA2* | *PPFIA4* | *PPFIBP1* | *PPFIBP2* | *PPIA* | *PPIAL4C* | *PPIAL4G* |
| *PPIB* | *PPM1B* | *PPM1E* | *PPM1F* | *PPM1H* | *PPM1J* | *PPM1L* | *PPM1N* |
| *PPP1CC* | *PPP1R14B* | *PPP1R16A* | *PPP1R16B* | *PPP1R2* | *PPP1R35* | *PPP1R3C* | *PPP4R3A* |
| *PPP4R3C* | *PQBP1* | *PRAC2* | *PRAME* | *PRAMEF4* | *PRAP1* | *PRC1* | *PRCP* |
| *PRDM13* | *PRDM4* | *PRELID3A* | *PRELP* | *PREP* | *PRF1* | *PRG2* | *PRIM1* |
| *PRKAG2* | *PRKCD* | *PRKCG* | *PRKCH* | *PRKCQ* | *PRKRIP1* | *PRKY* | *PRLR* |
| *PRMT1* | *PROCA1* | *PRODH* | *PROK2* | *PROSER1* | *PRPF4* | *PRR11* | *PRR16* |
| *PRR19* | *PRR5* | *PRR5L* | *PRR7* | *PRRX1* | *PRRX2* | *PRSS21* | *PRSS23* |
| *PRSS41* | *PRSS50* | *PRX* | *PSAP* | *PSAT1* | *PSG2* | *PSKH1* | *PSMA6* |
| *PSMA7* | *PSMB4* | *PSMB8* | *PSMC3IP* | *PSMD2* | *PSMD3* | *PSMD4* | *PSME3* |
| *PSMG1* | *PSMG3* | *PSPC1* | *PSPH* | *PSPN* | *PSRC1* | *PSTPIP1* | *PTAFR* |
| *PTBP1* | *PTCHD4* | *PTCRA* | *PTDSS1* | *PTDSS2* | *PTGDR* | *PTGDR2* | *PTGDS* |
| *PTGER2* | *PTGER4* | *PTGFRN* | *PTGIR* | *PTGIS* | *PTGS1* | *PTGS2* | *PTH2R* |
| *PTPN11* | *PTPN20* | *PTPN6* | *PTPN7* | *PTPRB* | *PTPRC* | *PTPRCAP* | *PTPRG* |
| *PTPRJ* | *PTPRT* | *PTRH2* | *PTX4* | *PUF60* | *PUS1* | *PVRIG* | *PXK* |
| *PYCR1* | *PYCR3* | *PYDC1* | *PYHIN1* | *QDPR* | *QPCT* | *QPCTL* | *QRFPR* |
| *QRSL1* | *QTRT1* | *RAB11FIP1* | *RAB11FIP4* | *RAB15* | *RAB1A* | *RAB20* | *RAB27B* |
| *RAB30* | *RAB38* | *RAB39B* | *RAB3A* | *RAB3B* | *RAB3C* | *RAB40B* | *RAB42* |
| *RAB6B* | *RABEP2* | *RAC3* | *RACGAP1* | *RAD21* | *RAD51AP1* | *RAD54B* | *RAD54L* |
| *RAD9A* | *RADX* | *RAE1* | *RAI14* | *RALB* | *RALGAPA2* | *RALGPS2* | *RAMP2* |
| *RAMP3* | *RANBP1* | *RANBP17* | *RANGRF* | *RAP1GAP* | *RAPGEF6* | *RARA* | *RARRES2* |
| *RASA1* | *RASA3* | *RASA4* | *RASGRF2* | *RASGRP1* | *RASGRP2* | *RASGRP3* | *RASIP1* |
| *RASSF4* | *RASSF5* | *RAX* | *RBBP8NL* | *RBCK1* | *RBIS* | *RBM20* | *RBM3* |
| *RBM5* | *RBMS3* | *RBP1* | *RBP3* | *RBPJ* | *RBPJL* | *RCAN3* | *RCC1* |
| *RCC2* | *RCN3* | *RCOR2* | *RCOR3* | *RCSD1* | *RDM1* | *RDX* | *REC8* |
| *RECQL4* | *REG4* | *RELA* | *RELL2* | *RELN* | *REM2* | *REN* | *RENBP* |
| *REPS1* | *REPS2* | *RET* | *RETNLB* | *RETREG1* | *REXO2* | *RFC3* | *RFC4* |
| *RFC5* | *RFX6* | *RFXANK* | *RGCC* | *RGL2* | *RGL3* | *RGMB* | *RGPD5* |
| *RGS1* | *RGS11* | *RGS13* | *RGS16* | *RGS17* | *RGS2* | *RGS20* | *RGS3* |
| *RGS4* | *RHBDD3* | *RHBG* | *RHEBL1* | *RHOA* | *RHOB* | *RHOJ* | *RHOT2* |
| *RHOU* | *RHPN1* | *RIBC2* | *RILPL2* | *RIMS2* | *RIMS4* | *RIPOR2* | *RIPPLY3* |
| *RLBP1* | *RLN2* | *RMI2* | *RMND1* | *RNASE10* | *RNASE2* | *RNASE3* | *RNASE6* |
| *RNASEH2A* | *RNF113B* | *RNF125* | *RNF128* | *RNF141* | *RNF144B* | *RNF175* | *RNF183* |
| *RNF185* | *RNF19A* | *RNF207* | *RNF208* | *RNF43* | *RNFT2* | *RNPS1* | *ROBO4* |
| *ROMO1* | *ROPN1B* | *RPIA* | *RPL10L* | *RPL21* | *RPL39L* | *RPP21* | *RPP38* |
| *RPRML* | *RPS2* | *RPS21* | *RPS24* | *RPS26* | *RPS6KC1* | *RPS6KL1* | *RPS7* |
| *RPS9* | *RPUSD1* | *RRAGD* | *RRM1* | *RRM2* | *RRP12* | *RRP9* | *RSAD2* |
| *RSPH14* | *RSRC1* | *RTBDN* | *RTEL1* | *RTKN2* | *RTL1* | *RTL9* | *RTP2* |
| *RTTN* | *RUBCNL* | *RUNX2* | *RUNX3* | *RUVBL2* | *RXRG* | *RYR1* | *S100A1* |
| *S100A12* | *S100A4* | *S100A5* | *S100A9* | *S100B* | *S1PR1* | *S1PR5* | *SAC3D1* |
| *SACM1L* | *SAGE1* | *SAMD1* | *SAMD10* | *SAMSN1* | *SAPCD2* | *SARDH* | *SARS2* |
| *SASH3* | *SASS6* | *SAV1* | *SBDS* | *SBK1* | *SCAMP1* | *SCAMP3* | *SCAMP5* |
| *SCARA3* | *SCARB2* | *SCARF2* | *SCG2* | *SCG3* | *SCG5* | *SCGB1D2* | *SCGN* |
| *SCHIP1* | *SCIN* | *SCN3A* | *SCN9A* | *SCNM1* | *SCNN1A* | *SCNN1D* | *SCRT1* |
| *SCUBE2* | *SCX* | *SDCBP* | *SDK1* | *SEC14L1* | *SEC31B* | *SEC61G* | *SELE* |
| *SELL* | *SELP* | *SELPLG* | *SEM1* | *SEMA3A* | *SEMA3B* | *SEMA3F* | *SEMA4A* |
| *SEMA6B* | *SEPTIN5* | *SEPTIN8* | *SERGEF* | *SERHL2* | *SERINC1* | *SERINC3* | *SERINC4* |
| *SERPINA1* | *SERPINA3* | *SERPINE2* | *SERPINF1* | *SERPINI1* | *SERTAD2* | *SESN3* | *SET* |
| *SETD7* | *SETDB1* | *SETMAR* | *SEZ6L* | *SEZ6L2* | *SF1* | *SF3B4* | *SFN* |
| *SFXN1* | *SFXN3* | *SGCB* | *SGK3* | *SGMS1* | *SGO1* | *SGO2* | *SGPL1* |
| *SGSM1* | *SH2B1* | *SH2D1A* | *SH2D1B* | *SH3D19* | *SH3KBP1* | *SH3RF1* | *SH3TC2* |
| *SHANK3* | *SHARPIN* | *SHC1* | *SHCBP1* | *SHE* | *SHISA7* | *SHLD1* | *SHOC1* |
| *SHOX2* | *SHROOM4* | *SIAH1* | *SIGLEC1* | *SIGLEC10* | *SIGLEC14* | *SIGLEC15* | *SIGLEC5* |
| *SIGLEC6* | *SIGLEC8* | *SIGLEC9* | *SIK1* | *SIRPA* | *SIRPB1* | *SIRPG* | *SIT1* |
| *SIX1* | *SIX3* | *SIX4* | *SKA1* | *SKA3* | *SKAP1* | *SKAP2* | *SKP1* |
| *SKP2* | *SLA* | *SLAMF1* | *SLAMF8* | *SLAMF9* | *SLBP* | *SLC11A1* | *SLC12A1* |
| *SLC12A2* | *SLC12A5* | *SLC12A6* | *SLC12A8* | *SLC15A2* | *SLC15A3* | *SLC15A4* | *SLC16A11* |
| *SLC16A14* | *SLC16A3* | *SLC16A6* | *SLC16A7* | *SLC16A8* | *SLC17A5* | *SLC17A9* | *SLC18A2* |
| *SLC1A3* | *SLC1A7* | *SLC22A15* | *SLC22A4* | *SLC24A2* | *SLC24A3* | *SLC24A5* | *SLC25A21* |
| *SLC25A35* | *SLC25A37* | *SLC25A40* | *SLC25A52* | *SLC25A6* | *SLC26A2* | *SLC26A5* | *SLC26A6* |
| *SLC26A8* | *SLC27A6* | *SLC29A1* | *SLC29A2* | *SLC29A3* | *SLC29A4* | *SLC2A1* | *SLC2A6* |
| *SLC30A3* | *SLC30A5* | *SLC30A8* | *SLC31A2* | *SLC35B3* | *SLC35D3* | *SLC35E3* | *SLC35G6* |
| *SLC38A1* | *SLC38A8* | *SLC39A14* | *SLC39A6* | *SLC44A2* | *SLC44A5* | *SLC45A2* | *SLC45A3* |
| *SLC4A8* | *SLC52A2* | *SLC6A3* | *SLC6A4* | *SLC7A10* | *SLC7A11* | *SLC7A5* | *SLC7A6* |
| *SLC7A7* | *SLC7A8* | *SLC9A5* | *SLC9C1* | *SLCO1C1* | *SLCO2A1* | *SLCO2B1* | *SLCO4C1* |
| *SLCO5A1* | *SLF1* | *SLFN13* | *SLIT1* | *SLITRK1* | *SLITRK6* | *SMAD1* | *SMAD2* |
| *SMAD4* | *SMC1B* | *SMC2* | *SMC4* | *SMC5* | *SMCO3* | *SMG1* | *SMG5* |
| *SMIM10L2A* | *SMIM10L2B* | *SMN1* | *SMPD3* | *SMPDL3B* | *SMS* | *SMYD2* | *SNAP47* |
| *SNCA* | *SNCG* | *SNF8* | *SNN* | *SNORC* | *SNRNP25* | *SNRNP40* | *SNRPA1* |
| *SNRPC* | *SNRPD1* | *SNRPE* | *SNRPN* | *SNTG1* | *SNURF* | *SNX13* | *SNX22* |
| *SNX24* | *SNX31* | *SNX9* | *SOCS1* | *SOD1* | *SORBS2* | *SORCS3* | *SORL1* |
| *SOSTDC1* | *SOX10* | *SOX11* | *SOX12* | *SOX17* | *SOX18* | *SOX2* | *SOX30* |
| *SOX5* | *SOX7* | *SOX9* | *SP140* | *SP6* | *SP7* | *SPAG4* | *SPATA12* |
| *SPATA25* | *SPATA9* | *SPC24* | *SPC25* | *SPCS3* | *SPDEF* | *SPDYA* | *SPDYC* |
| *SPDYE6* | *SPDYE8P* | *SPERT* | *SPG21* | *SPIB* | *SPINDOC* | *SPINK1* | *SPIRE1* |
| *SPN* | *SPOCK1* | *SPOCK2* | *SPON1* | *SPON2* | *SPP1* | *SPRY2* | *SPRY3* |
| *SPSB4* | *SPTAN1* | *SPTSSB* | *SPX* | *SQSTM1* | *SRA1* | *SRC* | *SRCIN1* |
| *SRD5A3* | *SRGN* | *SRL* | *SRMS* | *SRPK1* | *SRRM3* | *SRSF1* | *SRSF12* |
| *SRSF4* | *SRSF6* | *SRSF7* | *SRXN1* | *SS18L2* | *SSPO* | *SSU72* | *SSUH2* |
| *SSX1* | *SSX5* | *ST18* | *ST3GAL1* | *ST3GAL4* | *ST3GAL5* | *ST3GAL6* | *ST6GALNAC2* |
| *ST6GALNAC4* | *ST8SIA1* | *ST8SIA2* | *ST8SIA4* | *ST8SIA5* | *STAB1* | *STAC* | *STAG3* |
| *STAP1* | *STARD3* | *STAT1* | *STAT3* | *STAT4* | *STAT5A* | *STAT5B* | *STC2* |
| *STEAP1* | *STEAP4* | *STIL* | *STIM1* | *STK31* | *STK32A* | *STMN1* | *STON2* |
| *STPG2* | *STPG3* | *STPG4* | *STRA6* | *STRA8* | *STX10* | *STX16* | *STX18* |
| *STX1A* | *STX4* | *STXBP6* | *SUB1* | *SULF1* | *SULT1C2* | *SULT1C4* | *SULT4A1* |
| *SUSD3* | *SUV39H1* | *SV2C* | *SVEP1* | *SVOPL* | *SYCE1* | *SYCE1L* | *SYCE2* |
| *SYCE3* | *SYCP2* | *SYCP2L* | *SYN3* | *SYNE4* | *SYNGR3* | *SYNJ1* | *SYT10* |
| *SYT15* | *SYT17* | *SYT2* | *SYT5* | *SYT8* | *SYTL5* | *TAC3* | *TAC4* |
| *TACC3* | *TACSTD2* | *TAF15* | *TAF2* | *TAF4* | *TAF4B* | *TAF7L* | *TAFA2* |
| *TAFA3* | *TAGAP* | *TAGLN* | *TAGLN3* | *TAL1* | *TANGO2* | *TAP1* | *TAPBP* |
| *TARDBP* | *TAS1R1* | *TAS2R38* | *TASOR2* | *TBC1D1* | *TBC1D14* | *TBC1D24* | *TBC1D26* |
| *TBC1D3* | *TBC1D31* | *TBC1D3B* | *TBC1D3C* | *TBC1D3H* | *TBC1D4* | *TBC1D5* | *TBC1D9* |
| *TBCB* | *TBCC* | *TBCD* | *TBRG4* | *TBX2* | *TBX21* | *TBX6* | *TBXA2R* |
| *TBXAS1* | *TBXT* | *TCF15* | *TCF19* | *TCF7* | *TCL1A* | *TCL1B* | *TCP11* |
| *TCTN2* | *TCTN3* | *TDRD1* | *TDRD5* | *TDRD6* | *TEAD2* | *TEC* | *TECPR2* |
| *TEDC1* | *TEDC2* | *TEK* | *TEKT2* | *TEKT5* | *TENM1* | *TEP1* | *TERT* |
| *TESC* | *TESMIN* | *TEX14* | *TEX19* | *TEX264* | *TEX38* | *TEX45* | *TFAP2B* |
| *TFAP2E* | *TFCP2L1* | *TFDP3* | *TFEC* | *TFF1* | *TFF2* | *TFPI* | *TFPT* |
| *TFRC* | *TGFBR2* | *TGIF1* | *TGIF2LX* | *TGM2* | *TGM3* | *TGM5* | *THADA* |
| *THBD* | *THBS1* | *THBS2* | *THBS4* | *THEG* | *THEM6* | *THEMIS* | *THEMIS2* |
| *THOC6* | *THSD7B* | *THUMPD1* | *THUMPD2* | *THY1* | *TICRR* | *TIE1* | *TIGD3* |
| *TIGD5* | *TIGIT* | *TIMD4* | *TIMELESS* | *TIMM13* | *TIMM50* | *TIMM8A* | *TINAGL1* |
| *TIPARP* | *TIPIN* | *TK1* | *TKTL1* | *TLCD1* | *TLE3* | *TLE5* | *TLL2* |
| *TLR1* | *TLR10* | *TLR2* | *TLR7* | *TLR8* | *TLR9* | *TLX3* | *TM4SF18* |
| *TM4SF19* | *TM4SF4* | *TM6SF1* | *TM9SF4* | *TMBIM6* | *TMC6* | *TMED2* | *TMEFF2* |
| *TMEM101* | *TMEM116* | *TMEM119* | *TMEM129* | *TMEM132A* | *TMEM140* | *TMEM145* | *TMEM150C* |
| *TMEM156* | *TMEM160* | *TMEM161B* | *TMEM178A* | *TMEM198* | *TMEM205* | *TMEM211* | *TMEM213* |
| *TMEM222* | *TMEM223* | *TMEM255A* | *TMEM255B* | *TMEM259* | *TMEM270* | *TMEM31* | *TMEM33* |
| *TMEM38A* | *TMEM63C* | *TMEM65* | *TMEM74* | *TMEM81* | *TMEM8B* | *TMEM9* | *TMEM97* |
| *TMIE* | *TMPRSS3* | *TMPRSS9* | *TMSB15A* | *TNFAIP1* | *TNFAIP2* | *TNFAIP3* | *TNFAIP6* |
| *TNFRSF10C* | *TNFRSF11A* | *TNFRSF11B* | *TNFRSF13B* | *TNFRSF17* | *TNFRSF18* | *TNFRSF1A* | *TNFRSF1B* |
| *TNFRSF25* | *TNFRSF4* | *TNFRSF6B* | *TNFRSF8* | *TNFRSF9* | *TNFSF10* | *TNFSF13* | *TNFSF14* |
| *TNFSF15* | *TNFSF18* | *TNFSF4* | *TNFSF8* | *TNFSF9* | *TNIP3* | *TNK2* | *TOMM40L* |
| *TONSL* | *TOP1MT* | *TOP2A* | *TOPBP1* | *TOX* | *TOX4* | *TP53I13* | *TP53INP1* |
| *TP53RK* | *TP53TG3B* | *TP63* | *TPBG* | *TPI1* | *TPK1* | *TPM1* | *TPM2* |
| *TPSAB1* | *TPSB2* | *TPST1* | *TPTE* | *TPX2* | *TRADD* | *TRAF1* | *TRAF2* |
| *TRAF3IP2* | *TRAF3IP3* | *TRAF4* | *TRAF5* | *TRAIP* | *TRANK1* | *TRAPPC1* | *TRAPPC5* |
| *TRAT1* | *TREM1* | *TREM2* | *TREML1* | *TREML2* | *TREML4* | *TRHDE* | *TRIB2* |
| *TRIM11* | *TRIM16L* | *TRIM17* | *TRIM24* | *TRIM36* | *TRIM37* | *TRIM59* | *TRIM67* |
| *TRIM74* | *TRIM9* | *TRIP12* | *TRIP13* | *TRIT1* | *TRMO* | *TRMT12* | *TROAP* |
| *TRPM2* | *TRPM4* | *TRPM6* | *TRPM8* | *TRPS1* | *TRPV2* | *TRPV6* | *TSACC* |
| *TSC22D1* | *TSC22D3* | *TSGA13* | *TSHR* | *TSKS* | *TSLP* | *TSPAN15* | *TSPAN5* |
| *TSPAN7* | *TSSK1B* | *TSSK6* | *TSTA3* | *TTC38* | *TTC39A* | *TTC7B* | *TTK* |
| *TTLL4* | *TTYH3* | *TUBA3D* | *TUBA3E* | *TUBB* | *TUBB2B* | *TUBB3* | *TUBB4A* |
| *TUBB8* | *TUSC1* | *TUT7* | *TXK* | *TXNRD1* | *TXNRD2* | *TYK2* | *TYMP* |
| *TYMS* | *TYR* | *TYROBP* | *UAP1L1* | *UBA1* | *UBA2* | *UBA52* | *UBASH3A* |
| *UBD* | *UBE2C* | *UBE2D2* | *UBE2F* | *UBE2L6* | *UBE2Q1* | *UBE2QL1* | *UBE2S* |
| *UBE2T* | *UBL3* | *UCHL1* | *UCKL1* | *UCN* | *UFSP1* | *UGT1A8* | *UGT2B17* |
| *UHRF1* | *ULBP1* | *ULBP2* | *ULK1* | *UMODL1* | *UNC13A* | *UNC5A* | *UNC5D* |
| *UNC79* | *UPK1B* | *UPK2* | *UPK3A* | *UQCC3* | *UQCRB* | *UQCRC1* | *UQCRFS1* |
| *USB1* | *USF1* | *USO1* | *USP18* | *USP26* | *USP32* | *USP35* | *USP40* |
| *USP9Y* | *UTP20* | *UTS2B* | *VAC14* | *VAMP2* | *VANGL2* | *VAPA* | *VASH1* |
| *VASH2* | *VASN* | *VCAM1* | *VCAN* | *VCX* | *VCX3A* | *VEGFA* | *VEGFB* |
| *VEPH1* | *VGLL1* | *VGLL3* | *VHLL* | *VILL* | *VIM* | *VIRMA* | *VLDLR* |
| *VMAC* | *VN1R1* | *VNN1* | *VNN2* | *VNN3* | *VOPP1* | *VPREB3* | *VPS28* |
| *VSIG4* | *VSIR* | *VSTM2L* | *VTI1B* | *VWA5A* | *VWA5B1* | *VWA5B2* | *VWF* |
| *WARS* | *WASF1* | *WASHC5* | *WDHD1* | *WDR34* | *WDR48* | *WDR53* | *WDR66* |
| *WDR76* | *WDR83* | *WDR86* | *WDR87* | *WDR91* | *WDR97* | *WFDC10A* | *WFDC5* |
| *WFIKKN1* | *WINT* | *WIPF1* | *WIPI2* | *WNT10B* | *WNT5B* | *WNT7A* | *WNT7B* |
| *WRAP73* | *WWP1* | *XBP1* | *XCL1* | *XCL2* | *XKR4* | *XKR7* | *XKRX* |
| *XPO6* | *XPOT* | *XPR1* | *XRCC2* | *XRCC3* | *XRCC6* | *XXYLT1* | *YAE1* |
| *YBX1* | *YBX2* | *YEATS2* | *YEATS4* | *YIF1B* | *YIPF2* | *YJEFN3* | *YME1L1* |
| *ZAP70* | *ZBP1* | *ZBTB10* | *ZBTB18* | *ZBTB32* | *ZBTB41* | *ZBTB8B* | *ZC3H3* |
| *ZC3H7A* | *ZC3HC1* | *ZCCHC12* | *ZCRB1* | *ZDHHC23* | *ZDHHC9* | *ZEB1* | *ZEB2* |
| *ZFP36L1* | *ZFP36L2* | *ZFP69B* | *ZFR2* | *ZFYVE1* | *ZGLP1* | *ZGRF1* | *ZIC1* |
| *ZIC2* | *ZIC3* | *ZIC4* | *ZIC5* | *ZMIZ2* | *ZNF117* | *ZNF135* | *ZNF165* |
| *ZNF205* | *ZNF213* | *ZNF217* | *ZNF22* | *ZNF222* | *ZNF239* | *ZNF280A* | *ZNF286A* |
| *ZNF292* | *ZNF30* | *ZNF320* | *ZNF322* | *ZNF324* | *ZNF395* | *ZNF432* | *ZNF439* |
| *ZNF442* | *ZNF443* | *ZNF467* | *ZNF468* | *ZNF469* | *ZNF48* | *ZNF488* | *ZNF492* |
| *ZNF521* | *ZNF528* | *ZNF541* | *ZNF552* | *ZNF560* | *ZNF563* | *ZNF587* | *ZNF600* |
| *ZNF609* | *ZNF629* | *ZNF668* | *ZNF681* | *ZNF692* | *ZNF695* | *ZNF703* | *ZNF707* |
| *ZNF710* | *ZNF716* | *ZNF747* | *ZNF789* | *ZNF878* | *ZNF90* | *ZNF91* | *ZNF92* |
| *ZNF99* | *ZP3* | *ZPBP2* | *ZSCAN10* | *ZSCAN5B* |  |  |  |

**Table S3.** 993 differentially expressed genes for NMF clustering analysis.

| ID | conMean | treatMean | logFC | pValue | fdr |
| --- | --- | --- | --- | --- | --- |
| *SRPK1* | 4.352989 | 14.72825 | 1.758508 | 8.16E-33 | 2.86E-30 |
| *SRMS* | 0.225247 | 1.311985 | 2.542173 | 2.65E-11 | 5.28E-11 |
| *PPP1R16B* | 5.856515 | 2.919637 | -1.00425 | 8.97E-14 | 2.10E-13 |
| *PPP1R16A* | 2.946815 | 6.164365 | 1.064796 | 9.56E-15 | 2.37E-14 |
| *PPP1R14B* | 13.15027 | 61.00443 | 2.213822 | 1.63E-30 | 3.70E-29 |
| *ZP3* | 0.590835 | 2.662296 | 2.171845 | 1.11E-20 | 4.45E-20 |
| *SRD5A3* | 5.786994 | 19.88452 | 1.78076 | 1.29E-21 | 5.64E-21 |
| *DPEP2* | 6.718151 | 1.575505 | -2.09225 | 3.08E-31 | 1.15E-29 |
| *SRCIN1* | 0.378238 | 1.450282 | 1.938968 | 1.04E-14 | 2.58E-14 |
| *PPM1N* | 0.409649 | 0.926764 | 1.177814 | 1.46E-05 | 2.05E-05 |
| *MIA* | 0.072147 | 0.676101 | 3.228235 | 0.017813 | 0.02041 |
| *ZNF92* | 1.749819 | 4.071725 | 1.218435 | 7.83E-23 | 3.89E-22 |
| *MGST1* | 20.05196 | 43.54529 | 1.118774 | 1.39E-11 | 2.82E-11 |
| *DONSON* | 1.532786 | 4.853088 | 1.662747 | 6.66E-30 | 1.14E-28 |
| *CARD11* | 1.967008 | 7.4519 | 1.921605 | 1.82E-15 | 4.70E-15 |
| *PPM1J* | 0.2302 | 0.659306 | 1.518058 | 2.94E-14 | 7.08E-14 |
| *DOK5* | 0.272451 | 1.514902 | 2.475158 | 4.59E-15 | 1.16E-14 |
| *PPM1H* | 1.975339 | 4.601092 | 1.219876 | 1.00E-08 | 1.67E-08 |
| *PPM1F* | 7.065817 | 3.210309 | -1.13814 | 4.75E-25 | 3.01E-24 |
| *CAPN13* | 3.46192 | 11.85872 | 1.776304 | 0.01632 | 0.018759 |
| *SPTSSB* | 0.07051 | 1.543625 | 4.452357 | 2.64E-06 | 3.85E-06 |
| *MFSD3* | 5.148994 | 11.92749 | 1.211928 | 2.56E-15 | 6.54E-15 |
| *CAPN12* | 0.58385 | 2.178628 | 1.899751 | 1.44E-15 | 3.74E-15 |
| *CAMP* | 4.195653 | 0.758165 | -2.46831 | 5.57E-27 | 4.78E-26 |
| *ZNF710* | 5.30312 | 11.4648 | 1.112297 | 4.45E-10 | 8.11E-10 |
| *ZNF703* | 4.60463 | 9.424478 | 1.033327 | 0.030321 | 0.034189 |
| *SPP1* | 9.711535 | 292.0983 | 4.910611 | 3.12E-28 | 3.33E-27 |
| *MFAP4* | 242.8305 | 41.25859 | -2.55718 | 8.43E-31 | 2.38E-29 |
| *DNTTIP1* | 12.25776 | 39.44451 | 1.686129 | 1.09E-23 | 6.01E-23 |
| *MFAP3L* | 1.834475 | 0.493131 | -1.89533 | 2.96E-26 | 2.20E-25 |
| *DNMT3B* | 0.372217 | 1.737075 | 2.222443 | 1.59E-21 | 6.87E-21 |
| *CAMK2N2* | 0.057016 | 0.644613 | 3.498984 | 7.07E-18 | 2.18E-17 |
| *ZNF692* | 2.496827 | 8.136863 | 1.704377 | 5.16E-22 | 2.38E-21 |
| *MEX3A* | 0.269597 | 5.554331 | 4.364737 | 2.36E-31 | 1.01E-29 |
| *DNMT3A* | 2.297156 | 4.798356 | 1.062691 | 1.10E-18 | 3.67E-18 |
| *ZNF681* | 0.482503 | 1.219958 | 1.338222 | 3.16E-16 | 8.65E-16 |
| *SPOCK2* | 101.0418 | 12.0724 | -3.06517 | 5.64E-32 | 4.97E-30 |
| *PPFIA4* | 0.107195 | 0.64551 | 2.590206 | 1.00E-16 | 2.83E-16 |
| *SPOCK1* | 0.289325 | 2.798869 | 3.274082 | 9.63E-13 | 2.11E-12 |
| *DNM1* | 0.673236 | 1.599856 | 1.248757 | 8.87E-10 | 1.58E-09 |
| *SPN* | 14.89043 | 3.432099 | -2.11722 | 7.30E-30 | 1.23E-28 |
| *DNASE2B* | 3.030185 | 0.404203 | -2.90626 | 8.13E-26 | 5.71E-25 |
| *SPINK1* | 1.065996 | 132.0427 | 6.952659 | 1.41E-19 | 5.09E-19 |
| *PPBP* | 8.489927 | 2.451079 | -1.79234 | 1.43E-20 | 5.61E-20 |
| *HILPDA* | 4.722545 | 15.53461 | 1.71785 | 4.77E-14 | 1.14E-13 |
| *ZNF587* | 1.431903 | 3.100167 | 1.114412 | 1.58E-17 | 4.76E-17 |
| *PPAT* | 0.994408 | 4.311563 | 2.116301 | 1.70E-32 | 3.09E-30 |
| *DNASE1* | 0.723944 | 1.742239 | 1.266992 | 6.63E-17 | 1.90E-16 |
| *MET* | 16.60897 | 48.0633 | 1.532973 | 3.85E-05 | 5.26E-05 |
| *DNAJC9* | 2.291751 | 4.638659 | 1.017257 | 5.16E-26 | 3.70E-25 |
| *PPARG* | 12.57589 | 4.575455 | -1.45867 | 8.47E-24 | 4.71E-23 |
| *MEST* | 6.40837 | 16.91847 | 1.40057 | 6.21E-22 | 2.83E-21 |
| *MESP2* | 0.085953 | 0.852942 | 3.310829 | 5.69E-19 | 1.95E-18 |
| *DNAJC12* | 0.584311 | 12.55792 | 4.425717 | 2.69E-22 | 1.28E-21 |
| *CADM1* | 23.12667 | 9.86404 | -1.22931 | 7.42E-23 | 3.70E-22 |
| *POU6F2* | 0.038049 | 0.654066 | 4.103516 | 0.000149 | 0.000196 |
| *MESP1* | 0.35847 | 1.997026 | 2.47793 | 1.13E-20 | 4.52E-20 |
| *DNAJC10* | 3.896817 | 7.911562 | 1.021666 | 3.07E-22 | 1.44E-21 |
| *DNAJB13* | 0.582825 | 1.231905 | 1.079757 | 1.35E-05 | 1.89E-05 |
| *SPDYC* | 0.035847 | 0.840701 | 4.551682 | 0.042326 | 0.04714 |
| *MELTF* | 0.448349 | 3.413695 | 2.928642 | 6.72E-22 | 3.03E-21 |
| *HHIPL2* | 0.046749 | 3.178809 | 6.087408 | 2.08E-19 | 7.38E-19 |
| *MELK* | 0.402607 | 5.718715 | 3.828245 | 1.92E-30 | 4.27E-29 |
| *HHIP* | 9.819331 | 1.519131 | -2.69238 | 1.95E-28 | 2.20E-27 |
| *SPDEF* | 2.146498 | 22.67237 | 3.400878 | 2.27E-20 | 8.71E-20 |
| *POU3F2* | 0.008497 | 1.206676 | 7.149875 | 4.88E-11 | 9.57E-11 |
| *HGH1* | 5.578797 | 13.45038 | 1.269621 | 6.78E-25 | 4.23E-24 |
| *DNAH14* | 0.139989 | 0.715229 | 2.353096 | 4.06E-30 | 8.02E-29 |
| *CABYR* | 0.150209 | 4.063459 | 4.757668 | 1.19E-25 | 8.16E-25 |
| *ZNF469* | 0.354414 | 0.724037 | 1.030627 | 9.98E-07 | 1.49E-06 |
| *SPC25* | 0.523381 | 2.897109 | 2.46868 | 1.22E-26 | 9.76E-26 |
| *POU2AF1* | 1.200426 | 3.737149 | 1.638392 | 5.36E-11 | 1.05E-10 |
| *SPC24* | 0.473753 | 3.379883 | 2.834767 | 4.59E-30 | 8.61E-29 |
| *DNAAF3* | 2.208662 | 1.036709 | -1.09116 | 0.002939 | 0.003553 |
| *HES6* | 1.61151 | 12.49062 | 2.95436 | 1.28E-20 | 5.04E-20 |
| *DNA2* | 0.385501 | 2.077888 | 2.430312 | 1.92E-29 | 2.73E-28 |
| *CABLES2* | 1.779453 | 4.862405 | 1.450237 | 6.13E-26 | 4.34E-25 |
| *SPATA25* | 0.394364 | 0.827511 | 1.069252 | 5.12E-09 | 8.72E-09 |
| *CA9* | 0.281882 | 12.69819 | 5.493389 | 5.37E-23 | 2.73E-22 |
| *SPAG4* | 0.930701 | 7.662301 | 3.041388 | 2.22E-31 | 9.93E-30 |
| *DMRTA2* | 0.022715 | 0.834393 | 5.19902 | 1.05E-10 | 2.00E-10 |
| *CA4* | 19.03914 | 1.007447 | -4.24019 | 9.60E-32 | 6.35E-30 |
| *POLR2H* | 7.234796 | 17.67116 | 1.288373 | 2.02E-29 | 2.86E-28 |
| *POLQ* | 0.070026 | 0.80238 | 3.518329 | 4.65E-29 | 6.08E-28 |
| *ZNF322* | 0.938743 | 2.349388 | 1.323482 | 6.94E-11 | 1.35E-10 |
| *SOX9* | 3.478521 | 9.711437 | 1.481211 | 0.002667 | 0.003236 |
| *DMBX1* | 0.006703 | 0.650497 | 6.600696 | 4.14E-26 | 3.01E-25 |
| *C8orf76* | 1.859592 | 4.477425 | 1.267683 | 2.94E-25 | 1.92E-24 |
| *SOX7* | 10.65024 | 1.449953 | -2.87681 | 9.06E-30 | 1.46E-28 |
| *POLE2* | 0.424422 | 2.431185 | 2.51809 | 6.13E-30 | 1.07E-28 |
| *HELLS* | 0.2997 | 1.805519 | 2.590822 | 2.17E-29 | 3.04E-28 |
| *MDK* | 17.06061 | 156.1424 | 3.19412 | 1.44E-27 | 1.38E-26 |
| *HECW2* | 4.475699 | 1.965651 | -1.18711 | 2.09E-15 | 5.38E-15 |
| *DLX5* | 0.116256 | 0.885468 | 2.929139 | 1.64E-11 | 3.30E-11 |
| *C6orf52* | 0.692171 | 1.468968 | 1.085603 | 2.18E-07 | 3.38E-07 |
| *DLX4* | 0.41458 | 1.04356 | 1.33179 | 1.24E-07 | 1.95E-07 |
| *SOX2* | 2.754414 | 9.518892 | 1.789048 | 0.000662 | 0.000837 |
| *SOX18* | 11.00969 | 3.603775 | -1.61119 | 1.17E-18 | 3.87E-18 |
| *C5orf34* | 0.328779 | 1.512451 | 2.2017 | 7.48E-27 | 6.28E-26 |
| *ZNF239* | 0.750462 | 2.760981 | 1.879331 | 6.59E-27 | 5.57E-26 |
| *SOX17* | 6.396187 | 1.038249 | -2.62306 | 6.30E-29 | 7.96E-28 |
| *MCM8* | 0.850042 | 2.482988 | 1.546472 | 1.30E-24 | 7.91E-24 |
| *SOX12* | 2.764752 | 7.781966 | 1.492985 | 1.10E-20 | 4.38E-20 |
| *PODXL2* | 2.401586 | 26.07017 | 3.44034 | 1.26E-20 | 4.97E-20 |
| *MCM7* | 11.43002 | 30.77776 | 1.42906 | 6.09E-23 | 3.07E-22 |
| *HCN3* | 0.490683 | 1.850005 | 1.914667 | 1.94E-23 | 1.03E-22 |
| *DLGAP5* | 0.346882 | 5.137896 | 3.88866 | 4.89E-31 | 1.58E-29 |
| *C5AR1* | 32.43466 | 9.236383 | -1.81214 | 8.61E-28 | 8.48E-27 |
| *SOX11* | 0.019175 | 0.663151 | 5.112022 | 8.44E-11 | 1.63E-10 |
| *MCM6* | 4.709643 | 14.72009 | 1.644097 | 2.21E-25 | 1.47E-24 |
| *HCN2* | 0.098642 | 0.56118 | 2.508192 | 1.02E-14 | 2.52E-14 |
| *ZNF217* | 5.847695 | 16.70864 | 1.514655 | 3.04E-27 | 2.73E-26 |
| *SOSTDC1* | 14.36616 | 1.160037 | -3.63043 | 9.96E-29 | 1.20E-27 |
| *MCM4* | 2.747187 | 14.61356 | 2.411281 | 4.31E-31 | 1.44E-29 |
| *HCK* | 27.235 | 12.18452 | -1.16041 | 1.63E-21 | 7.02E-21 |
| *DLC1* | 32.43916 | 7.59919 | -2.09382 | 4.79E-28 | 4.94E-27 |
| *SORL1* | 3.917988 | 7.927698 | 1.016789 | 5.92E-06 | 8.45E-06 |
| *POC1A* | 1.399206 | 4.649928 | 1.7326 | 8.40E-26 | 5.89E-25 |
| *MCM3* | 14.18669 | 29.41833 | 1.052177 | 1.44E-22 | 6.99E-22 |
| *HCAR1* | 0.360457 | 2.382978 | 2.724864 | 3.24E-10 | 5.96E-10 |
| *C2CD4D* | 0.186724 | 1.174861 | 2.653513 | 4.70E-25 | 2.99E-24 |
| *MCM2* | 2.708009 | 12.56983 | 2.214661 | 2.11E-28 | 2.35E-27 |
| *HBEGF* | 49.49853 | 9.092514 | -2.44463 | 3.77E-27 | 3.29E-26 |
| *ZNF117* | 2.126456 | 6.449473 | 1.600731 | 6.41E-10 | 1.16E-09 |
| *PNPLA6* | 26.22616 | 9.791153 | -1.42146 | 9.54E-30 | 1.53E-28 |
| *MCM10* | 0.143942 | 1.928689 | 3.744065 | 6.59E-30 | 1.13E-28 |
| *DIRAS1* | 0.306747 | 2.023946 | 2.722052 | 2.06E-12 | 4.40E-12 |
| *PNOC* | 0.293806 | 0.883825 | 1.588895 | 2.75E-11 | 5.46E-11 |
| *HAVCR1* | 0.035796 | 1.36622 | 5.254251 | 4.54E-16 | 1.23E-15 |
| *DIPK2B* | 6.339549 | 1.984291 | -1.67576 | 1.58E-21 | 6.84E-21 |
| *PNMA3* | 0.108079 | 0.716048 | 2.727976 | 6.55E-06 | 9.35E-06 |
| *MC1R* | 0.459412 | 1.108808 | 1.27115 | 1.01E-13 | 2.35E-13 |
| *C1QTNF6* | 0.912997 | 4.745129 | 2.377766 | 1.32E-27 | 1.26E-26 |
| *C1QTNF3* | 1.37541 | 3.170027 | 1.204633 | 0.00353 | 0.004247 |
| *ZIC2* | 0.01499 | 0.61159 | 5.350516 | 9.53E-12 | 1.95E-11 |
| *SNX22* | 3.788973 | 1.110622 | -1.77044 | 8.96E-28 | 8.74E-27 |
| *HASPIN* | 0.254063 | 1.104893 | 2.120651 | 1.18E-21 | 5.20E-21 |
| *DHX34* | 2.354022 | 4.972987 | 1.078985 | 1.35E-20 | 5.32E-20 |
| *C1QTNF12* | 0.224147 | 0.862328 | 1.943795 | 2.08E-08 | 3.43E-08 |
| *HAPLN3* | 2.312079 | 5.791045 | 1.324633 | 5.19E-13 | 1.15E-12 |
| *ZGRF1* | 0.260521 | 0.640167 | 1.297046 | 7.09E-15 | 1.78E-14 |
| *HAL* | 0.347448 | 2.285429 | 2.717595 | 8.42E-07 | 1.27E-06 |
| *DHRS11* | 1.006726 | 2.321275 | 1.205247 | 7.49E-23 | 3.73E-22 |
| *MB* | 0.450496 | 4.945197 | 3.456442 | 7.47E-21 | 3.02E-20 |
| *HAGHL* | 0.575599 | 1.511342 | 1.392694 | 2.22E-12 | 4.75E-12 |
| *DHFR* | 1.404145 | 3.002358 | 1.096404 | 1.56E-21 | 6.80E-21 |
| *C1QB* | 423.3749 | 158.7529 | -1.41515 | 5.59E-22 | 2.56E-21 |
| *SNRPE* | 15.36675 | 36.66644 | 1.254648 | 3.51E-28 | 3.72E-27 |
| *PMEL* | 0.516205 | 1.117101 | 1.113743 | 2.88E-18 | 9.24E-18 |
| *C1QA* | 437.5651 | 164.1559 | -1.41443 | 7.94E-24 | 4.44E-23 |
| *ZFP69B* | 0.31234 | 1.146911 | 1.876563 | 4.51E-28 | 4.68E-27 |
| *SNRPA1* | 4.166967 | 8.807067 | 1.079664 | 2.09E-27 | 1.95E-26 |
| *MARCO* | 213.6366 | 28.35032 | -2.91372 | 8.89E-31 | 2.44E-29 |
| *DGKA* | 1.578806 | 3.28428 | 1.056743 | 3.18E-15 | 8.10E-15 |
| *C1orf162* | 24.38726 | 7.645354 | -1.67347 | 2.66E-29 | 3.64E-28 |
| *ZEB2* | 5.379776 | 2.452373 | -1.13337 | 1.88E-25 | 1.26E-24 |
| *PLXNA3* | 2.942397 | 6.566949 | 1.158232 | 1.22E-14 | 2.99E-14 |
| *MARCKSL1* | 32.16467 | 128.8418 | 2.002052 | 5.26E-26 | 3.76E-25 |
| *C1orf159* | 1.073031 | 2.201417 | 1.03674 | 2.72E-18 | 8.77E-18 |
| *ZEB1* | 6.84112 | 2.896401 | -1.23997 | 2.37E-23 | 1.25E-22 |
| *SNRNP25* | 4.953916 | 10.25772 | 1.050069 | 1.14E-21 | 5.04E-21 |
| *DGAT2* | 1.021776 | 2.077008 | 1.023428 | 1.82E-07 | 2.84E-07 |
| *C1orf112* | 0.470943 | 1.769429 | 1.909658 | 1.24E-31 | 7.51E-30 |
| *ZDHHC9* | 15.32092 | 41.23605 | 1.428403 | 1.41E-21 | 6.13E-21 |
| *DEPP1* | 130.5576 | 40.16192 | -1.70079 | 9.65E-11 | 1.86E-10 |
| *MAPK8IP2* | 0.604346 | 1.908123 | 1.658708 | 6.45E-05 | 8.70E-05 |
| *DEPDC1B* | 0.166192 | 2.295764 | 3.788056 | 4.31E-31 | 1.44E-29 |
| *PLPP5* | 6.112726 | 14.67547 | 1.263518 | 1.33E-22 | 6.48E-22 |
| *DEPDC1* | 0.129407 | 2.150493 | 4.054682 | 1.23E-30 | 3.22E-29 |
| *C19orf48* | 4.917753 | 14.56664 | 1.566597 | 5.96E-26 | 4.22E-25 |
| *SNCA* | 1.911225 | 0.739917 | -1.36906 | 1.20E-24 | 7.35E-24 |
| *PLPP2* | 2.282388 | 13.31086 | 2.543988 | 2.43E-27 | 2.23E-26 |
| *MAP7D2* | 0.229047 | 2.727416 | 3.573819 | 6.81E-18 | 2.10E-17 |
| *PLLP* | 10.02337 | 3.638223 | -1.46206 | 4.16E-25 | 2.66E-24 |
| *ZBTB41* | 2.619787 | 5.575114 | 1.089552 | 1.60E-22 | 7.73E-22 |
| *SMPDL3B* | 6.562855 | 26.73304 | 2.026229 | 1.72E-21 | 7.38E-21 |
| *PLK4* | 0.478094 | 2.254034 | 2.237144 | 9.63E-27 | 7.89E-26 |
| *PLK1* | 0.583106 | 6.020276 | 3.368 | 1.52E-31 | 8.32E-30 |
| *C12orf45* | 3.484196 | 7.522208 | 1.110331 | 9.24E-26 | 6.43E-25 |
| *GTSE1* | 0.29638 | 2.843547 | 3.26217 | 4.50E-30 | 8.55E-29 |
| *C11orf80* | 2.25032 | 6.78139 | 1.591451 | 8.88E-29 | 1.09E-27 |
| *MAOB* | 18.10087 | 6.806286 | -1.41112 | 1.31E-24 | 7.96E-24 |
| *ZBP1* | 0.478856 | 1.191918 | 1.315622 | 8.21E-09 | 1.38E-08 |
| *PLEKHO2* | 28.3315 | 13.60483 | -1.05829 | 6.74E-24 | 3.80E-23 |
| *MANEAL* | 1.723628 | 7.019767 | 2.025975 | 4.55E-21 | 1.88E-20 |
| *PLEKHH2* | 5.275675 | 2.449204 | -1.10704 | 7.03E-16 | 1.87E-15 |
| *PLEKHG4* | 1.380354 | 3.436724 | 1.315996 | 1.62E-08 | 2.68E-08 |
| *BZW2* | 10.29276 | 28.60763 | 1.47477 | 3.55E-30 | 7.21E-29 |
| *MAL* | 9.438946 | 4.139444 | -1.18919 | 4.68E-19 | 1.61E-18 |
| *BUB1B* | 0.314032 | 3.781494 | 3.589972 | 7.60E-32 | 5.67E-30 |
| *DDX39A* | 7.69665 | 15.98855 | 1.054736 | 7.79E-22 | 3.48E-21 |
| *BUB1* | 0.62409 | 4.526035 | 2.858422 | 9.41E-28 | 9.15E-27 |
| *SMC1B* | 0.048935 | 0.633604 | 3.694639 | 1.38E-10 | 2.60E-10 |
| *YBX2* | 0.047443 | 1.411564 | 4.894943 | 1.64E-19 | 5.89E-19 |
| *DDX11* | 1.143918 | 3.379254 | 1.562721 | 2.66E-20 | 1.02E-19 |
| *PLEK2* | 1.818444 | 12.71499 | 2.805754 | 8.61E-30 | 1.41E-28 |
| *PLD5* | 0.084266 | 0.680373 | 3.013297 | 2.56E-05 | 3.53E-05 |
| *MAGED1* | 21.88399 | 47.13335 | 1.106872 | 1.94E-22 | 9.31E-22 |
| *DCUN1D5* | 7.821629 | 15.78403 | 1.012925 | 4.22E-24 | 2.41E-23 |
| *DCSTAMP* | 3.039118 | 0.700077 | -2.11807 | 2.10E-19 | 7.47E-19 |
| *BST1* | 4.971782 | 2.456348 | -1.01725 | 6.43E-22 | 2.91E-21 |
| *XRCC2* | 0.147288 | 1.607316 | 3.447943 | 1.01E-31 | 6.55E-30 |
| *SLFN13* | 1.648564 | 5.496611 | 1.737332 | 2.43E-20 | 9.28E-20 |
| *PLCH2* | 0.285708 | 0.678919 | 1.248699 | 0.001089 | 0.001352 |
| *MAGEC1* | 0.001815 | 0.683143 | 8.555929 | 2.49E-10 | 4.64E-10 |
| *GRIN2D* | 0.245827 | 1.601763 | 2.703944 | 1.05E-11 | 2.15E-11 |
| *DCST2* | 0.157231 | 0.860516 | 2.452316 | 1.92E-20 | 7.39E-20 |
| *XPR1* | 4.926139 | 20.95233 | 2.088581 | 5.14E-29 | 6.70E-28 |
| *SLF1* | 0.845006 | 1.875008 | 1.149862 | 1.23E-12 | 2.68E-12 |
| *XPOT* | 7.575858 | 16.20679 | 1.097117 | 1.12E-22 | 5.50E-22 |
| *MAGEA3* | 0.018609 | 5.980589 | 8.328138 | 4.24E-07 | 6.50E-07 |
| *DCN* | 81.1062 | 32.12571 | -1.33608 | 2.94E-21 | 1.23E-20 |
| *SLCO4C1* | 7.61264 | 3.753517 | -1.02015 | 2.13E-18 | 6.90E-18 |
| *XKRX* | 0.661761 | 5.174405 | 2.967011 | 3.78E-16 | 1.03E-15 |
| *SLCO2B1* | 23.5775 | 9.651246 | -1.28862 | 2.16E-23 | 1.15E-22 |
| *MAGEA12* | 0.016598 | 2.952377 | 7.474708 | 5.96E-05 | 8.06E-05 |
| *GREM1* | 0.137989 | 4.824581 | 5.127774 | 2.79E-26 | 2.08E-25 |
| *BRIX1* | 3.710012 | 9.727215 | 1.390603 | 6.17E-29 | 7.84E-28 |
| *SLCO2A1* | 43.16359 | 13.51357 | -1.67541 | 4.71E-21 | 1.94E-20 |
| *PLAU* | 10.34143 | 70.0441 | 2.759828 | 6.58E-19 | 2.23E-18 |
| *GRB7* | 6.043449 | 14.34794 | 1.2474 | 4.13E-16 | 1.13E-15 |
| *BRIP1* | 0.150966 | 1.123745 | 2.896021 | 1.31E-29 | 2.00E-28 |
| *MAGEA10* | 0.005698 | 1.539996 | 8.078366 | 4.77E-08 | 7.69E-08 |
| *SLC9A5* | 0.484391 | 1.036249 | 1.097127 | 6.35E-07 | 9.63E-07 |
| *PLAC9* | 19.92259 | 3.218057 | -2.63014 | 3.04E-32 | 4.01E-30 |
| *MAD2L2* | 4.640173 | 9.573382 | 1.04485 | 1.95E-18 | 6.34E-18 |
| *GPX2* | 1.210418 | 74.35104 | 5.940775 | 3.05E-07 | 4.70E-07 |
| *DCAF13* | 2.372023 | 6.733995 | 1.505345 | 1.36E-30 | 3.34E-29 |
| *BRDT* | 0.022642 | 1.767773 | 6.286782 | 8.76E-09 | 1.47E-08 |
| *SLC7A7* | 18.40958 | 7.397012 | -1.31544 | 2.76E-26 | 2.07E-25 |
| *MAD2L1* | 0.579801 | 3.84288 | 2.728559 | 4.42E-29 | 5.86E-28 |
| *GPT2* | 1.069293 | 12.05352 | 3.494725 | 4.27E-32 | 4.19E-30 |
| *BRCA2* | 0.340299 | 0.859717 | 1.337058 | 9.90E-15 | 2.45E-14 |
| *PLA2G4F* | 14.76384 | 2.43654 | -2.59916 | 6.39E-30 | 1.11E-28 |
| *MACC1* | 3.860145 | 8.740011 | 1.17898 | 3.09E-05 | 4.25E-05 |
| *BRCA1* | 0.68536 | 2.173766 | 1.665262 | 1.81E-18 | 5.93E-18 |
| *SLC7A5* | 4.345734 | 31.80058 | 2.871381 | 1.07E-25 | 7.35E-25 |
| *GPRIN1* | 0.371255 | 2.749967 | 2.88893 | 8.53E-29 | 1.05E-27 |
| *DBNDD1* | 1.701339 | 7.345261 | 2.110143 | 1.11E-26 | 8.97E-26 |
| *WNT7A* | 3.141122 | 0.895295 | -1.81084 | 5.91E-26 | 4.21E-25 |
| *SLC7A11* | 0.542026 | 4.924974 | 3.183683 | 5.74E-17 | 1.65E-16 |
| *PLA2G4A* | 3.680481 | 21.7583 | 2.5636 | 1.58E-11 | 3.18E-11 |
| *DBF4* | 1.139521 | 3.45921 | 1.602016 | 5.69E-27 | 4.87E-26 |
| *WNT5B* | 1.193463 | 2.458779 | 1.042788 | 0.00094 | 0.001172 |
| *SLC7A10* | 0.038562 | 1.17668 | 4.93141 | 6.23E-11 | 1.21E-10 |
| *PLA2G2D* | 1.772855 | 4.273007 | 1.269177 | 4.08E-05 | 5.57E-05 |
| *GPR89B* | 0.334212 | 0.731997 | 1.131077 | 6.20E-23 | 3.12E-22 |
| *BPIFA2* | 0.107993 | 4.890765 | 5.501053 | 4.38E-11 | 8.61E-11 |
| *SLC6A4* | 30.43752 | 0.468094 | -6.02291 | 3.97E-32 | 4.12E-30 |
| *LYVE1* | 19.14665 | 2.282895 | -3.06816 | 1.66E-30 | 3.75E-29 |
| *SLC6A3* | 0.134443 | 2.426578 | 4.173859 | 0.00318 | 0.003837 |
| *SLC52A2* | 10.22851 | 30.29232 | 1.566356 | 1.17E-27 | 1.13E-26 |
| *PKMYT1* | 0.289649 | 2.578992 | 3.15443 | 9.34E-29 | 1.14E-27 |
| *LYPD6B* | 0.453017 | 1.869489 | 2.045008 | 0.002425 | 0.00295 |
| *BORA* | 0.774581 | 1.91907 | 1.308918 | 1.91E-21 | 8.15E-21 |
| *PKM* | 78.26624 | 163.1892 | 1.060084 | 1.30E-24 | 7.91E-24 |
| *GPR4* | 6.3075 | 2.267904 | -1.47571 | 1.66E-20 | 6.46E-20 |
| *DAPK2* | 5.495389 | 1.585183 | -1.79357 | 2.22E-27 | 2.06E-26 |
| *BOP1* | 5.027141 | 18.00809 | 1.840835 | 8.87E-28 | 8.68E-27 |
| *LYG1* | 0.360158 | 0.915362 | 1.345713 | 4.39E-11 | 8.62E-11 |
| *DAP3* | 13.37249 | 27.56502 | 1.043571 | 1.38E-30 | 3.34E-29 |
| *BMX* | 1.642254 | 0.795037 | -1.04658 | 8.74E-27 | 7.22E-26 |
| *SLC44A5* | 0.344983 | 2.377036 | 2.784565 | 1.96E-10 | 3.66E-10 |
| *LY86* | 19.64255 | 8.093155 | -1.27921 | 7.46E-22 | 3.34E-21 |
| *DACH1* | 3.919325 | 0.761755 | -2.36321 | 1.23E-28 | 1.45E-27 |
| *WDR86* | 0.44959 | 2.393422 | 2.412394 | 8.79E-13 | 1.93E-12 |
| *GPR18* | 0.445599 | 0.983005 | 1.141455 | 0.001804 | 0.002211 |
| *WDR76* | 1.172473 | 2.920003 | 1.316416 | 6.48E-19 | 2.20E-18 |
| *GPR174* | 0.572581 | 1.147242 | 1.002617 | 0.016401 | 0.018844 |
| *PIP5KL1* | 0.926478 | 3.049653 | 1.718817 | 1.95E-14 | 4.76E-14 |
| *BLM* | 0.316683 | 1.485516 | 2.229852 | 6.56E-29 | 8.22E-28 |
| *PIMREG* | 0.25399 | 2.585192 | 3.347425 | 1.78E-28 | 2.02E-27 |
| *WDHD1* | 0.722926 | 2.874323 | 1.991303 | 8.40E-27 | 6.98E-26 |
| *SLC31A2* | 2.502222 | 1.000644 | -1.32228 | 7.87E-24 | 4.42E-23 |
| *GPR146* | 2.086247 | 0.459549 | -2.18262 | 2.21E-29 | 3.07E-28 |
| *PIM2* | 13.1391 | 28.67397 | 1.125875 | 2.72E-10 | 5.03E-10 |
| *GPR143* | 0.760316 | 1.938129 | 1.349993 | 1.68E-06 | 2.49E-06 |
| *BIRC5* | 0.820054 | 11.80302 | 3.847293 | 6.39E-30 | 1.11E-28 |
| *WASF1* | 1.078817 | 2.964911 | 1.458538 | 2.05E-15 | 5.27E-15 |
| *PILRB* | 0.597908 | 1.399615 | 1.227033 | 4.45E-07 | 6.80E-07 |
| *LST1* | 19.09739 | 8.209161 | -1.21807 | 1.76E-20 | 6.83E-20 |
| *PILRA* | 15.05187 | 5.845312 | -1.36459 | 2.50E-25 | 1.65E-24 |
| *CYP27C1* | 0.099814 | 0.8813 | 3.14232 | 2.29E-15 | 5.87E-15 |
| *VWF* | 94.4423 | 20.41827 | -2.20957 | 5.81E-29 | 7.44E-28 |
| *CYP27B1* | 0.426547 | 2.496409 | 2.549077 | 3.26E-27 | 2.91E-26 |
| *SLC2A1* | 3.179845 | 39.69872 | 3.642064 | 1.64E-29 | 2.41E-28 |
| *CYP27A1* | 69.98145 | 22.7298 | -1.62239 | 1.34E-26 | 1.06E-25 |
| *BIK* | 1.942429 | 8.241626 | 2.085067 | 3.51E-20 | 1.32E-19 |
| *SLC29A4* | 0.557806 | 5.347639 | 3.261067 | 6.73E-20 | 2.48E-19 |
| *GPM6B* | 5.959398 | 1.680879 | -1.82595 | 1.74E-27 | 1.64E-26 |
| *GPI* | 17.91683 | 42.96202 | 1.261746 | 1.06E-26 | 8.57E-26 |
| *VSTM2L* | 9.212068 | 41.83391 | 2.183076 | 9.60E-10 | 1.71E-09 |
| *GPC3* | 49.33581 | 12.33918 | -1.99939 | 1.62E-27 | 1.54E-26 |
| *BEX5* | 7.381243 | 3.306961 | -1.15836 | 1.97E-14 | 4.82E-14 |
| *VSIR* | 28.30477 | 10.04426 | -1.49467 | 1.23E-31 | 7.51E-30 |
| *LRRN4CL* | 1.083209 | 0.491688 | -1.1395 | 3.03E-13 | 6.86E-13 |
| *GPC2* | 0.073945 | 0.849056 | 3.52133 | 1.22E-23 | 6.68E-23 |
| *CYBRD1* | 86.19152 | 27.30487 | -1.65839 | 4.04E-28 | 4.26E-27 |
| *VSIG4* | 114.2998 | 24.24591 | -2.23701 | 2.38E-28 | 2.61E-27 |
| *LRRN3* | 3.839126 | 0.681607 | -2.49376 | 1.75E-27 | 1.66E-26 |
| *CYBB* | 63.76422 | 28.89203 | -1.14208 | 1.04E-15 | 2.71E-15 |
| *SLC26A6* | 1.257216 | 3.47943 | 1.468618 | 2.58E-21 | 1.09E-20 |
| *PIF1* | 0.174935 | 0.967428 | 2.467336 | 1.08E-23 | 5.97E-23 |
| *BCL6B* | 9.795456 | 3.102763 | -1.65856 | 3.19E-26 | 2.36E-25 |
| *PIAS3* | 5.84088 | 13.42836 | 1.201025 | 3.51E-25 | 2.27E-24 |
| *BCL2L12* | 3.751123 | 8.051174 | 1.101877 | 6.26E-22 | 2.85E-21 |
| *GOLM1* | 10.89091 | 74.72642 | 2.778494 | 3.45E-32 | 4.12E-30 |
| *CXCR2* | 3.087709 | 0.555968 | -2.47346 | 2.17E-26 | 1.65E-25 |
| *BCL2L10* | 0.032172 | 0.630065 | 4.291636 | 2.67E-20 | 1.02E-19 |
| *LRRC4* | 5.315897 | 2.46611 | -1.10808 | 5.22E-14 | 1.24E-13 |
| *CXCR1* | 2.614492 | 0.382055 | -2.77468 | 5.32E-23 | 2.72E-22 |
| *LRRC26* | 0.125696 | 0.750124 | 2.577188 | 0.012756 | 0.014767 |
| *CXCL9* | 14.77223 | 41.83907 | 1.501963 | 0.000368 | 0.000473 |
| *BCL2A1* | 21.61206 | 10.0169 | -1.1094 | 1.56E-15 | 4.05E-15 |
| *GOLGA7B* | 0.625429 | 4.460883 | 2.834411 | 3.72E-20 | 1.40E-19 |
| *PHKA1* | 1.228794 | 4.343352 | 1.821566 | 4.99E-30 | 9.18E-29 |
| *CXCL3* | 9.212927 | 3.088262 | -1.57686 | 3.59E-10 | 6.59E-10 |
| *VIM* | 320.4624 | 154.8834 | -1.04897 | 1.40E-25 | 9.54E-25 |
| *LRP8* | 0.762242 | 2.12255 | 1.477477 | 4.86E-15 | 1.22E-14 |
| *CXCL14* | 4.211762 | 88.11883 | 4.386955 | 6.12E-11 | 1.19E-10 |
| *SLC22A4* | 3.182655 | 1.456332 | -1.12789 | 2.97E-07 | 4.59E-07 |
| *PHEX* | 1.087726 | 0.519629 | -1.06576 | 2.01E-13 | 4.59E-13 |
| *GNG11* | 39.59972 | 12.93439 | -1.61428 | 3.99E-23 | 2.06E-22 |
| *CXCL13* | 8.6819 | 25.86476 | 1.574905 | 7.90E-17 | 2.25E-16 |
| *VGLL3* | 4.937211 | 2.411747 | -1.03362 | 2.87E-20 | 1.09E-19 |
| *CXCL12* | 14.63733 | 6.623824 | -1.14392 | 9.86E-20 | 3.61E-19 |
| *VEPH1* | 13.43918 | 2.393849 | -2.48904 | 2.27E-30 | 4.85E-29 |
| *CXCL10* | 17.22468 | 36.94841 | 1.101035 | 0.001239 | 0.001532 |
| *SLC17A9* | 0.903836 | 5.230738 | 2.532881 | 1.20E-20 | 4.77E-20 |
| *GMNN* | 2.868758 | 8.153463 | 1.506987 | 9.15E-26 | 6.38E-25 |
| *BASP1* | 11.93982 | 44.57806 | 1.900552 | 6.37E-12 | 1.32E-11 |
| *PGGHG* | 7.979354 | 31.50464 | 1.981221 | 8.25E-09 | 1.39E-08 |
| *GMDS* | 2.596589 | 7.556456 | 1.541092 | 7.34E-28 | 7.29E-27 |
| *CUZD1* | 0.214271 | 0.734916 | 1.778145 | 7.92E-10 | 1.42E-09 |
| *BARX2* | 0.201597 | 3.449077 | 4.096664 | 3.57E-20 | 1.34E-19 |
| *SLC16A8* | 0.155324 | 0.779151 | 2.326626 | 1.15E-12 | 2.50E-12 |
| *PGF* | 0.823307 | 2.350999 | 1.513772 | 1.26E-13 | 2.92E-13 |
| *BARX1* | 0.045892 | 9.584237 | 7.706276 | 3.83E-17 | 1.13E-16 |
| *VCAN* | 5.032767 | 19.74955 | 1.972396 | 5.04E-14 | 1.20E-13 |
| *SLC16A7* | 0.826673 | 2.081568 | 1.332281 | 7.83E-06 | 1.11E-05 |
| *LRFN4* | 2.513533 | 6.268606 | 1.318428 | 2.44E-09 | 4.23E-09 |
| *GLYATL2* | 0.041851 | 1.236778 | 4.885192 | 0.002455 | 0.002986 |
| *SLC16A6* | 2.455498 | 1.179288 | -1.0581 | 9.57E-13 | 2.10E-12 |
| *SLC16A3* | 8.115203 | 23.07699 | 1.507756 | 2.87E-16 | 7.89E-16 |
| *PFN2* | 10.0982 | 30.60841 | 1.59983 | 1.20E-09 | 2.12E-09 |
| *SLC16A14* | 1.019131 | 7.918393 | 2.957868 | 6.74E-12 | 1.39E-11 |
| *PFKP* | 7.575957 | 36.97509 | 2.287054 | 1.61E-24 | 9.67E-24 |
| *SLC16A11* | 3.697145 | 1.254535 | -1.55926 | 6.92E-24 | 3.89E-23 |
| *LPGAT1* | 5.6419 | 13.58678 | 1.267951 | 1.80E-25 | 1.20E-24 |
| *CTSV* | 0.421326 | 2.202376 | 2.386052 | 8.19E-12 | 1.69E-11 |
| *BACE2* | 4.020775 | 11.78311 | 1.551175 | 1.07E-10 | 2.05E-10 |
| *CTSK* | 20.99102 | 42.64497 | 1.022603 | 6.53E-07 | 9.88E-07 |
| *SLC15A3* | 18.83824 | 7.975572 | -1.24 | 2.24E-23 | 1.19E-22 |
| *PFDN2* | 39.40628 | 81.88787 | 1.055224 | 5.37E-23 | 2.73E-22 |
| *LPAR2* | 3.239565 | 6.859883 | 1.082384 | 4.11E-21 | 1.70E-20 |
| *CTSG* | 3.698904 | 1.021836 | -1.85593 | 1.89E-14 | 4.63E-14 |
| *VAMP2* | 37.358 | 18.18227 | -1.03888 | 2.40E-27 | 2.22E-26 |
| *SLC15A2* | 12.25159 | 4.463674 | -1.45667 | 3.20E-25 | 2.08E-24 |
| *PF4* | 2.277067 | 0.575505 | -1.98428 | 1.46E-19 | 5.27E-19 |
| *SLC12A8* | 1.763956 | 4.852049 | 1.45978 | 9.73E-21 | 3.90E-20 |
| *B4GALT3* | 8.81085 | 19.16524 | 1.121139 | 1.78E-28 | 2.02E-27 |
| *GLDN* | 4.56762 | 0.899951 | -2.34352 | 9.91E-27 | 8.05E-26 |
| *B4GALNT1* | 0.129443 | 0.814877 | 2.654267 | 1.09E-10 | 2.07E-10 |
| *GLDC* | 0.186123 | 1.431987 | 2.94369 | 2.42E-08 | 3.97E-08 |
| *B3GNT4* | 0.129418 | 0.600503 | 2.214131 | 2.54E-16 | 7.00E-16 |
| *GLB1L3* | 0.068913 | 2.813019 | 5.351199 | 1.55E-08 | 2.56E-08 |
| *B3GAT1* | 0.11366 | 0.621935 | 2.452033 | 0.007427 | 0.008737 |
| *PECAM1* | 113.5645 | 24.83482 | -2.19308 | 5.59E-33 | 2.86E-30 |
| *LMNB2* | 6.656206 | 15.33466 | 1.204025 | 1.57E-17 | 4.73E-17 |
| *B3GALNT1* | 8.489887 | 3.791737 | -1.16289 | 2.64E-20 | 1.01E-19 |
| *SLC11A1* | 13.1425 | 3.962841 | -1.72963 | 2.38E-24 | 1.39E-23 |
| *PEAR1* | 4.917019 | 1.294037 | -1.92591 | 5.81E-29 | 7.44E-28 |
| *LMNB1* | 3.386752 | 14.01504 | 2.049002 | 4.41E-27 | 3.82E-26 |
| *CTLA4* | 0.952502 | 2.220242 | 1.220923 | 2.81E-09 | 4.86E-09 |
| *PDZD11* | 14.9467 | 30.74272 | 1.040418 | 4.98E-24 | 2.83E-23 |
| *SLAMF9* | 0.252892 | 1.05179 | 2.056252 | 6.94E-11 | 1.35E-10 |
| *AURKB* | 0.576621 | 6.779661 | 3.555518 | 3.66E-30 | 7.34E-29 |
| *SKP2* | 2.227744 | 6.054302 | 1.442377 | 2.36E-18 | 7.62E-18 |
| *PDP1* | 4.753439 | 9.784443 | 1.041518 | 1.79E-12 | 3.83E-12 |
| *LIPA* | 73.00013 | 33.53952 | -1.12204 | 3.07E-13 | 6.95E-13 |
| *GINS4* | 0.242055 | 1.484124 | 2.616206 | 1.73E-29 | 2.51E-28 |
| *AURKA* | 1.253187 | 8.944746 | 2.835439 | 2.37E-29 | 3.29E-28 |
| *PDLIM4* | 2.810596 | 7.206246 | 1.358372 | 1.16E-06 | 1.73E-06 |
| *GINS2* | 0.496589 | 4.097023 | 3.044453 | 4.54E-30 | 8.58E-29 |
| *AUNIP* | 0.223007 | 1.281319 | 2.522471 | 8.64E-26 | 6.04E-25 |
| *LINGO1* | 0.206719 | 1.264049 | 2.612308 | 7.80E-16 | 2.07E-15 |
| *GINS1* | 0.454325 | 4.27598 | 3.234459 | 3.92E-32 | 4.12E-30 |
| *CST1* | 0.311099 | 25.45048 | 6.354175 | 3.04E-25 | 1.98E-24 |
| *PDK1* | 0.668499 | 2.519559 | 1.914174 | 1.50E-28 | 1.76E-27 |
| *GIMAP5* | 1.328682 | 0.48114 | -1.46547 | 3.80E-22 | 1.76E-21 |
| *UQCC3* | 4.334049 | 9.772981 | 1.173083 | 7.16E-21 | 2.90E-20 |
| *SKA3* | 0.245803 | 2.559057 | 3.380036 | 1.18E-30 | 3.11E-29 |
| *PDIA4* | 41.64848 | 158.9367 | 1.932117 | 3.01E-31 | 1.14E-29 |
| *LIN7A* | 3.599559 | 0.713834 | -2.33416 | 1.38E-30 | 3.34E-29 |
| *UPK3A* | 0.170779 | 1.670507 | 3.290084 | 1.25E-10 | 2.36E-10 |
| *SKA1* | 0.239519 | 2.22256 | 3.214013 | 1.71E-28 | 1.96E-27 |
| *PDGFRL* | 2.418613 | 7.418172 | 1.616884 | 2.36E-16 | 6.52E-16 |
| *UPK2* | 0.288245 | 1.011619 | 1.811297 | 0.008025 | 0.009422 |
| *SIX4* | 0.705821 | 3.096906 | 2.133453 | 7.94E-24 | 4.44E-23 |
| *GGH* | 3.333126 | 10.11721 | 1.601863 | 3.93E-08 | 6.36E-08 |
| *SIX1* | 0.981005 | 5.692156 | 2.536643 | 6.45E-18 | 2.00E-17 |
| *GGCT* | 10.07999 | 36.31863 | 1.849216 | 4.76E-32 | 4.46E-30 |
| *PDF* | 0.693571 | 1.612843 | 1.217491 | 3.29E-21 | 1.37E-20 |
| *LIMK1* | 6.235939 | 15.18947 | 1.284393 | 9.49E-21 | 3.81E-20 |
| *GFRA3* | 1.464363 | 12.29097 | 3.069254 | 0.000831 | 0.001041 |
| *ATP6V1C2* | 0.418035 | 3.661251 | 3.130641 | 2.78E-23 | 1.46E-22 |
| *SIRPG* | 0.632001 | 1.866529 | 1.56236 | 3.90E-13 | 8.76E-13 |
| *LIME1* | 0.435717 | 1.17869 | 1.435721 | 2.53E-12 | 5.40E-12 |
| *GFRA1* | 1.643245 | 0.554907 | -1.56623 | 7.43E-20 | 2.73E-19 |
| *SIRPB1* | 5.56308 | 0.861128 | -2.69158 | 1.58E-30 | 3.61E-29 |
| *UMODL1* | 0.050566 | 1.03563 | 4.35621 | 9.14E-13 | 2.01E-12 |
| *SIRPA* | 26.08732 | 11.93659 | -1.12796 | 1.53E-22 | 7.42E-22 |
| *PDE2A* | 2.806664 | 0.901475 | -1.6385 | 1.91E-24 | 1.13E-23 |
| *GFOD1* | 2.766104 | 0.887094 | -1.6407 | 5.67E-25 | 3.58E-24 |
| *ATP6V0D2* | 3.217253 | 1.147406 | -1.48745 | 0.002643 | 0.003209 |
| *PDCD5* | 10.95598 | 22.51523 | 1.039183 | 9.80E-24 | 5.42E-23 |
| *LILRA6* | 2.460055 | 1.020976 | -1.26874 | 1.97E-19 | 7.02E-19 |
| *UHRF1* | 0.291512 | 3.367557 | 3.530074 | 4.84E-31 | 1.58E-29 |
| *SIGLEC6* | 1.090425 | 0.52269 | -1.06086 | 1.83E-16 | 5.08E-16 |
| *PDCD2L* | 2.100053 | 6.489331 | 1.627644 | 5.63E-29 | 7.28E-28 |
| *LILRA5* | 8.633873 | 2.291627 | -1.91364 | 1.30E-26 | 1.04E-25 |
| *GEMIN2* | 2.184983 | 4.412433 | 1.013952 | 3.98E-15 | 1.00E-14 |
| *CSAG1* | 0.023101 | 3.780728 | 7.354557 | 1.19E-07 | 1.88E-07 |
| *PDCD1* | 0.953591 | 2.074048 | 1.121007 | 2.18E-06 | 3.20E-06 |
| *UFSP1* | 0.903948 | 2.018241 | 1.158787 | 1.32E-13 | 3.05E-13 |
| *SIGLEC14* | 5.679287 | 2.413295 | -1.23471 | 5.39E-17 | 1.56E-16 |
| *PCSK9* | 7.681051 | 2.96162 | -1.37492 | 4.36E-21 | 1.80E-20 |
| *UCN* | 0.545546 | 1.555178 | 1.511308 | 9.88E-11 | 1.90E-10 |
| *LIG1* | 2.63579 | 5.347747 | 1.020696 | 5.20E-20 | 1.94E-19 |
| *SIGLEC1* | 9.491113 | 4.34949 | -1.12573 | 1.02E-17 | 3.12E-17 |
| *CRYAB* | 13.50757 | 3.767833 | -1.84196 | 3.66E-27 | 3.22E-26 |
| *UCHL1* | 2.778538 | 34.27293 | 3.624672 | 2.28E-10 | 4.25E-10 |
| *CRY2* | 16.22663 | 7.9792 | -1.02405 | 2.30E-22 | 1.10E-21 |
| *ATP1A3* | 0.188491 | 0.85411 | 2.179926 | 1.36E-05 | 1.91E-05 |
| *SHROOM4* | 7.677313 | 2.576234 | -1.57534 | 3.88E-24 | 2.24E-23 |
| *PCOLCE2* | 9.903489 | 2.162324 | -2.19535 | 2.83E-25 | 1.85E-24 |
| *GDF15* | 14.89709 | 47.10163 | 1.660746 | 1.96E-05 | 2.72E-05 |
| *UBE2T* | 1.771084 | 19.25115 | 3.44224 | 3.00E-32 | 4.01E-30 |
| *SHOX2* | 0.045667 | 0.576562 | 3.658238 | 1.03E-15 | 2.70E-15 |
| *LGSN* | 0.141796 | 4.17581 | 4.880167 | 1.06E-15 | 2.77E-15 |
| *UBE2S* | 2.097127 | 7.586483 | 1.855017 | 1.79E-20 | 6.93E-20 |
| *SHOC1* | 0.074214 | 0.767457 | 3.370329 | 1.85E-11 | 3.72E-11 |
| *PCNA* | 26.98007 | 65.04043 | 1.269443 | 4.14E-24 | 2.37E-23 |
| *LFNG* | 8.638184 | 19.4653 | 1.172105 | 2.99E-07 | 4.61E-07 |
| *GCSH* | 1.017168 | 2.268863 | 1.157411 | 1.96E-23 | 1.04E-22 |
| *ATIC* | 10.37542 | 26.80955 | 1.369577 | 1.41E-30 | 3.35E-29 |
| *SHCBP1* | 0.507138 | 2.809787 | 2.47001 | 3.39E-27 | 3.00E-26 |
| *PCLAF* | 0.633038 | 5.040345 | 2.993158 | 1.51E-30 | 3.52E-29 |
| *UBE2C* | 1.635823 | 33.50758 | 4.356399 | 8.79E-31 | 2.44E-29 |
| *UBD* | 3.667382 | 13.7296 | 1.904467 | 1.36E-07 | 2.13E-07 |
| *SHANK3* | 8.452463 | 3.357949 | -1.33179 | 1.21E-19 | 4.41E-19 |
| *LDHA* | 51.30674 | 144.9287 | 1.498123 | 2.40E-28 | 2.63E-27 |
| *ATAD5* | 0.395924 | 1.41038 | 1.832787 | 1.50E-23 | 8.19E-23 |
| *LDB2* | 17.21839 | 3.020543 | -2.51107 | 3.76E-32 | 4.12E-30 |
| *ATAD3B* | 1.319174 | 3.9446 | 1.580244 | 6.47E-21 | 2.63E-20 |
| *ATAD2* | 1.885261 | 7.76266 | 2.041787 | 1.33E-26 | 1.05E-25 |
| *TYROBP* | 229.8088 | 88.81406 | -1.37157 | 8.48E-23 | 4.20E-22 |
| *GATA2* | 10.26017 | 3.002139 | -1.77299 | 1.74E-24 | 1.04E-23 |
| *PCDHB2* | 0.978928 | 2.156815 | 1.139627 | 0.002469 | 0.003001 |
| *LCN12* | 0.153612 | 0.86753 | 2.497625 | 4.93E-15 | 1.24E-14 |
| *CREB3L4* | 3.381634 | 9.647337 | 1.51241 | 8.78E-24 | 4.87E-23 |
| *ASPM* | 0.151103 | 2.377935 | 3.976108 | 3.18E-31 | 1.15E-29 |
| *TYMS* | 2.492057 | 10.49907 | 2.074852 | 3.74E-27 | 3.27E-26 |
| *ASPHD1* | 0.71628 | 5.318051 | 2.892302 | 5.52E-17 | 1.60E-16 |
| *SGPL1* | 7.312836 | 16.39058 | 1.164364 | 7.13E-28 | 7.12E-27 |
| *PCDHB11* | 0.647764 | 1.332692 | 1.040804 | 0.013992 | 0.01614 |
| *GAPDH* | 217.5321 | 786.7382 | 1.854655 | 5.50E-28 | 5.62E-27 |
| *ASPH* | 9.40845 | 24.03844 | 1.353314 | 7.09E-08 | 1.13E-07 |
| *SGO2* | 0.571776 | 1.958912 | 1.776531 | 1.80E-23 | 9.73E-23 |
| *CRACR2B* | 4.394407 | 9.687604 | 1.140471 | 8.66E-07 | 1.30E-06 |
| *ASNS* | 2.845765 | 10.04325 | 1.819338 | 2.05E-27 | 1.92E-26 |
| *SGO1* | 0.128079 | 1.351128 | 3.39906 | 4.06E-30 | 8.02E-29 |
| *ASIC1* | 0.264807 | 0.719162 | 1.441374 | 2.12E-09 | 3.71E-09 |
| *TXNRD1* | 20.41002 | 67.0796 | 1.716596 | 6.34E-06 | 9.04E-06 |
| *CPXM1* | 1.59176 | 6.917741 | 2.119678 | 3.43E-18 | 1.09E-17 |
| *ASGR1* | 2.077435 | 0.964776 | -1.10654 | 1.26E-19 | 4.59E-19 |
| *GALNT14* | 0.360678 | 5.091862 | 3.819411 | 1.92E-20 | 7.39E-20 |
| *CPT1B* | 0.186009 | 0.747218 | 2.006158 | 6.73E-16 | 1.79E-15 |
| *ASF1B* | 1.544808 | 9.080425 | 2.555332 | 2.43E-28 | 2.63E-27 |
| *SFXN1* | 2.150144 | 6.872635 | 1.67643 | 1.31E-32 | 2.94E-30 |
| *LAPTM5* | 200.1391 | 99.59205 | -1.0069 | 1.79E-19 | 6.40E-19 |
| *TUBB4A* | 0.196416 | 1.074512 | 2.451698 | 0.020945 | 0.023867 |
| *SFN* | 68.39786 | 142.6068 | 1.06002 | 2.16E-11 | 4.32E-11 |
| *LAPTM4B* | 37.77517 | 116.8438 | 1.629071 | 5.21E-22 | 2.39E-21 |
| *CPSF1* | 9.264999 | 19.08209 | 1.042356 | 6.40E-18 | 1.99E-17 |
| *ASCL2* | 0.737366 | 1.85536 | 1.331246 | 0.008442 | 0.009887 |
| *TUBB3* | 0.07057 | 2.100897 | 4.895801 | 1.29E-30 | 3.27E-29 |
| *TUBB2B* | 0.391012 | 7.028499 | 4.167931 | 7.78E-11 | 1.50E-10 |
| *CPNE7* | 0.297301 | 3.025504 | 3.34718 | 1.71E-24 | 1.02E-23 |
| *SEZ6L2* | 7.000847 | 25.99992 | 1.892906 | 9.41E-14 | 2.20E-13 |
| *LAMP5* | 1.046737 | 3.149242 | 1.589106 | 9.00E-12 | 1.85E-11 |
| *CPNE5* | 1.024771 | 2.534944 | 1.306652 | 4.85E-13 | 1.08E-12 |
| *LAMP3* | 172.7551 | 28.24311 | -2.61276 | 2.39E-31 | 1.01E-29 |
| *ASB16* | 0.388486 | 0.817438 | 1.073246 | 1.49E-10 | 2.80E-10 |
| *TTYH3* | 9.716089 | 27.58169 | 1.505263 | 1.13E-21 | 5.00E-21 |
| *PBK* | 0.418174 | 4.860407 | 3.5389 | 2.38E-28 | 2.61E-27 |
| *TTK* | 0.22584 | 2.58221 | 3.515232 | 4.84E-30 | 8.96E-29 |
| *PAX7* | 0.022517 | 1.92511 | 6.417792 | 9.03E-08 | 1.44E-07 |
| *CPLX1* | 0.977086 | 2.254111 | 1.206001 | 3.75E-05 | 5.12E-05 |
| *ARRB1* | 22.24055 | 10.29667 | -1.11101 | 7.10E-25 | 4.42E-24 |
| *GABRD* | 0.471051 | 1.037979 | 1.139823 | 1.75E-08 | 2.88E-08 |
| *CPD* | 10.11854 | 31.15003 | 1.622232 | 4.60E-17 | 1.34E-16 |
| *LAGE3* | 10.35428 | 25.7707 | 1.315504 | 6.90E-22 | 3.10E-21 |
| *CPA3* | 43.3576 | 14.49602 | -1.58063 | 1.70E-20 | 6.61E-20 |
| *SERPINE2* | 1.494262 | 4.695875 | 1.651961 | 2.07E-08 | 3.41E-08 |
| *LAG3* | 1.153203 | 3.164653 | 1.456401 | 8.35E-07 | 1.25E-06 |
| *GABRA3* | 0.010112 | 0.715863 | 6.14548 | 2.90E-05 | 4.00E-05 |
| *LACTB2* | 9.602531 | 19.55192 | 1.025824 | 5.52E-14 | 1.31E-13 |
| *TSPAN7* | 27.15386 | 8.363492 | -1.69898 | 5.16E-24 | 2.93E-23 |
| *PARPBP* | 0.320719 | 1.565638 | 2.287367 | 2.73E-27 | 2.47E-26 |
| *TSPAN5* | 2.04412 | 4.920885 | 1.267438 | 4.34E-13 | 9.70E-13 |
| *ARL9* | 0.165239 | 1.007699 | 2.608442 | 6.34E-16 | 1.70E-15 |
| *PAQR6* | 0.456026 | 1.542476 | 1.758059 | 4.99E-10 | 9.06E-10 |
| *G6PD* | 18.99821 | 52.44721 | 1.465003 | 1.58E-05 | 2.20E-05 |
| *SERINC1* | 127.3172 | 62.50241 | -1.02644 | 2.86E-31 | 1.12E-29 |
| *PAQR5* | 5.59646 | 2.36873 | -1.2404 | 1.89E-22 | 9.09E-22 |
| *KRTCAP2* | 3.612515 | 8.187816 | 1.180475 | 3.00E-24 | 1.74E-23 |
| *PAQR4* | 2.13932 | 6.563586 | 1.617332 | 7.42E-23 | 3.70E-22 |
| *PAPSS2* | 55.91883 | 22.05491 | -1.34223 | 1.01E-25 | 6.97E-25 |
| *KRT80* | 2.569474 | 15.97717 | 2.636467 | 3.51E-24 | 2.03E-23 |
| *PANX2* | 0.942245 | 5.772589 | 2.615045 | 1.37E-18 | 4.52E-18 |
| *FZD4* | 12.89915 | 4.612064 | -1.48379 | 1.98E-24 | 1.16E-23 |
| *KREMEN2* | 0.075597 | 0.633324 | 3.066546 | 8.42E-21 | 3.40E-20 |
| *FZD3* | 0.515923 | 1.131997 | 1.133642 | 6.79E-09 | 1.15E-08 |
| *ARHGEF39* | 0.202547 | 1.198219 | 2.564563 | 9.41E-32 | 6.35E-30 |
| *TSACC* | 0.090561 | 0.834791 | 3.204449 | 4.27E-30 | 8.39E-29 |
| *PALMD* | 5.832183 | 1.541761 | -1.91946 | 6.99E-28 | 7.01E-27 |
| *KPNA7* | 0.468422 | 2.510169 | 2.421903 | 1.58E-19 | 5.69E-19 |
| *ARHGEF15* | 10.18454 | 1.978411 | -2.36397 | 5.21E-31 | 1.65E-29 |
| *TRPV2* | 26.12591 | 7.638584 | -1.7741 | 3.33E-30 | 6.87E-29 |
| *SEMA3B* | 19.20601 | 6.722344 | -1.51452 | 3.63E-22 | 1.69E-21 |
| *KPNA2* | 8.503594 | 37.66968 | 2.147259 | 2.70E-27 | 2.46E-26 |
| *SEMA3A* | 0.773772 | 2.956698 | 1.934007 | 0.000303 | 0.000391 |
| *PAFAH1B3* | 5.402157 | 27.44948 | 2.345171 | 9.34E-29 | 1.14E-27 |
| *KNTC1* | 0.599531 | 3.002634 | 2.324322 | 1.92E-29 | 2.73E-28 |
| *FXYD6* | 11.42335 | 3.264302 | -1.80714 | 5.20E-30 | 9.45E-29 |
| *TRPM8* | 0.008731 | 1.415488 | 7.340974 | 2.01E-26 | 1.54E-25 |
| *SEM1* | 3.912605 | 8.325003 | 1.089321 | 7.30E-25 | 4.53E-24 |
| *KNOP1* | 1.117031 | 2.78278 | 1.316858 | 1.70E-30 | 3.80E-29 |
| *COLEC12* | 18.39124 | 6.260262 | -1.55472 | 3.87E-26 | 2.83E-25 |
| *SELPLG* | 39.24446 | 15.70779 | -1.32101 | 1.56E-26 | 1.22E-25 |
| *PADI3* | 0.012801 | 1.938819 | 7.242831 | 2.44E-07 | 3.78E-07 |
| *KNL1* | 0.167614 | 1.191284 | 2.829301 | 1.02E-28 | 1.23E-27 |
| *COLCA2* | 1.288977 | 2.625226 | 1.026215 | 0.030528 | 0.034383 |
| *SELP* | 10.5655 | 2.279699 | -2.21245 | 1.03E-29 | 1.61E-28 |
| *PACSIN1* | 0.096287 | 0.617175 | 2.680264 | 4.61E-19 | 1.59E-18 |
| *FUT8* | 3.446398 | 10.67844 | 1.63154 | 9.67E-25 | 5.94E-24 |
| *COL9A2* | 1.817182 | 7.834016 | 2.108049 | 2.24E-09 | 3.91E-09 |
| *TRPM2* | 1.081246 | 2.739358 | 1.341143 | 4.20E-13 | 9.40E-13 |
| *PACRG* | 2.087824 | 0.511298 | -2.02976 | 7.26E-17 | 2.08E-16 |
| *TROAP* | 0.248269 | 3.887893 | 3.969011 | 2.92E-31 | 1.13E-29 |
| *SELE* | 4.712789 | 0.695966 | -2.75949 | 4.79E-10 | 8.72E-10 |
| *SEC61G* | 18.17299 | 39.36572 | 1.115144 | 6.09E-23 | 3.07E-22 |
| *KLRF1* | 2.366262 | 0.874522 | -1.43604 | 3.14E-16 | 8.59E-16 |
| *COL6A3* | 15.09356 | 40.60885 | 1.427861 | 7.27E-12 | 1.50E-11 |
| *PABPC1L* | 1.642303 | 7.709408 | 2.2309 | 8.81E-19 | 2.96E-18 |
| *FURIN* | 40.21692 | 86.72655 | 1.108671 | 0.008375 | 0.009812 |
| *ARHGAP11A* | 0.640823 | 3.098938 | 2.273776 | 4.64E-27 | 4.00E-26 |
| *PABPC1* | 151.6175 | 350.6882 | 1.209753 | 7.07E-26 | 4.99E-25 |
| *TRIP13* | 1.025104 | 6.941103 | 2.759395 | 2.81E-24 | 1.64E-23 |
| *SDK1* | 1.539694 | 3.842238 | 1.319303 | 0.000177 | 0.000232 |
| *P4HB* | 110.3752 | 259.4978 | 1.233307 | 1.97E-28 | 2.22E-27 |
| *P4HA1* | 18.91614 | 40.10955 | 1.084328 | 3.57E-18 | 1.13E-17 |
| *COL5A2* | 6.278432 | 32.26774 | 2.361616 | 6.26E-22 | 2.85E-21 |
| *AQP9* | 12.05712 | 4.581112 | -1.39612 | 6.45E-18 | 2.00E-17 |
| *SCX* | 0.693879 | 2.65657 | 1.936808 | 1.29E-10 | 2.44E-10 |
| *COL4A4* | 7.014719 | 3.409447 | -1.04085 | 2.37E-15 | 6.05E-15 |
| *P2RY14* | 3.595882 | 1.464817 | -1.29563 | 1.55E-20 | 6.05E-20 |
| *COL3A1* | 53.39215 | 354.5232 | 2.73118 | 5.98E-23 | 3.02E-22 |
| *TRIM59* | 0.468272 | 1.488546 | 1.668487 | 3.45E-25 | 2.23E-24 |
| *SCNN1D* | 0.38432 | 1.167358 | 1.602865 | 5.49E-09 | 9.33E-09 |
| *P2RX5* | 0.247207 | 0.64358 | 1.380399 | 5.72E-12 | 1.19E-11 |
| *FSCN1* | 16.12579 | 34.01811 | 1.076933 | 0.001299 | 0.001602 |
| *APOLD1* | 10.80325 | 3.142231 | -1.7816 | 3.62E-13 | 8.16E-13 |
| *P2RX1* | 2.171192 | 1.0252 | -1.08258 | 6.94E-17 | 1.99E-16 |
| *FRY* | 4.703357 | 1.820934 | -1.36901 | 1.43E-26 | 1.12E-25 |
| *COL1A2* | 58.42295 | 185.8753 | 1.669728 | 5.05E-13 | 1.12E-12 |
| *APOL3* | 15.09687 | 6.563581 | -1.20169 | 1.31E-20 | 5.16E-20 |
| *TRIM17* | 0.33248 | 1.60738 | 2.273372 | 5.40E-09 | 9.19E-09 |
| *COL1A1* | 37.78217 | 336.3963 | 3.154384 | 2.64E-24 | 1.54E-23 |
| *TRIM16L* | 1.025593 | 4.1098 | 2.00261 | 6.87E-07 | 1.04E-06 |
| *SCIN* | 1.360274 | 4.342589 | 1.674658 | 0.001307 | 0.001612 |
| *KLHL35* | 0.471113 | 1.293436 | 1.457065 | 5.45E-07 | 8.27E-07 |
| *FRMD4A* | 2.349291 | 1.048869 | -1.16339 | 6.29E-25 | 3.93E-24 |
| *APOC1* | 213.0832 | 85.23053 | -1.32197 | 5.68E-18 | 1.78E-17 |
| *FRMD3* | 2.925496 | 0.604369 | -2.27518 | 1.90E-29 | 2.73E-28 |
| *COL11A2* | 0.121981 | 0.90646 | 2.893584 | 0.005911 | 0.007008 |
| *FRK* | 1.455829 | 3.1478 | 1.112502 | 1.80E-15 | 4.64E-15 |
| *COCH* | 0.146735 | 2.204477 | 3.909152 | 5.25E-19 | 1.80E-18 |
| *APOBEC3A* | 1.816199 | 0.81468 | -1.15662 | 1.45E-11 | 2.92E-11 |
| *SCG5* | 0.645673 | 5.983347 | 3.212077 | 7.73E-21 | 3.12E-20 |
| *KLHL17* | 1.054098 | 3.082968 | 1.548311 | 1.92E-19 | 6.86E-19 |
| *COA6* | 8.721053 | 21.22039 | 1.282877 | 1.12E-22 | 5.50E-22 |
| *SCG2* | 0.866321 | 9.72614 | 3.488894 | 0.012427 | 0.014398 |
| *FPR2* | 6.389966 | 0.953004 | -2.74525 | 3.26E-27 | 2.91E-26 |
| *APLN* | 12.66982 | 4.307941 | -1.55633 | 5.00E-15 | 1.26E-14 |
| *FPR1* | 19.58376 | 5.767473 | -1.76365 | 2.62E-19 | 9.22E-19 |
| *KLF9* | 32.52535 | 11.33096 | -1.52129 | 5.99E-22 | 2.74E-21 |
| *FOXRED2* | 2.850322 | 6.1565 | 1.110986 | 1.74E-12 | 3.74E-12 |
| *APBA2* | 0.686857 | 3.046882 | 2.149253 | 1.70E-12 | 3.65E-12 |
| *SCAMP5* | 1.50098 | 3.867988 | 1.365678 | 7.86E-16 | 2.08E-15 |
| *ORC6* | 0.217158 | 1.982874 | 3.190777 | 1.63E-31 | 8.42E-30 |
| *FOXP3* | 0.606962 | 2.384704 | 1.974133 | 6.84E-22 | 3.08E-21 |
| *CNR1* | 2.408119 | 0.959918 | -1.32692 | 5.15E-17 | 1.49E-16 |
| *ORC1* | 0.342382 | 2.594703 | 2.92189 | 1.31E-29 | 2.00E-28 |
| *TRAIP* | 0.442967 | 1.735077 | 1.969728 | 9.64E-30 | 1.54E-28 |
| *SBK1* | 1.055422 | 7.11406 | 2.752853 | 3.09E-20 | 1.17E-19 |
| *KISS1R* | 0.015324 | 1.235215 | 6.332815 | 1.65E-28 | 1.90E-27 |
| *FOXM1* | 0.681631 | 8.028514 | 3.55807 | 1.45E-30 | 3.43E-29 |
| *CNN1* | 12.15908 | 4.624901 | -1.39454 | 2.21E-18 | 7.17E-18 |
| *TRAF5* | 1.232607 | 3.341421 | 1.438749 | 1.57E-18 | 5.15E-18 |
| *KISS1* | 0.315365 | 1.399932 | 2.150263 | 1.28E-06 | 1.90E-06 |
| *AP1S1* | 17.17008 | 40.96177 | 1.254381 | 1.98E-25 | 1.32E-24 |
| *TRAF4* | 9.338725 | 25.98969 | 1.476642 | 2.15E-28 | 2.39E-27 |
| *FOXI3* | 0.010007 | 1.111428 | 6.795189 | 1.39E-14 | 3.43E-14 |
| *SASS6* | 0.819574 | 2.171628 | 1.40583 | 1.78E-22 | 8.58E-22 |
| *AOX1* | 4.600314 | 2.276671 | -1.01481 | 1.98E-17 | 5.93E-17 |
| *AOC1* | 0.341036 | 11.1892 | 5.036037 | 7.25E-15 | 1.81E-14 |
| *TRAF2* | 4.272344 | 9.270445 | 1.117611 | 1.33E-20 | 5.23E-20 |
| *SARS2* | 0.980426 | 2.006756 | 1.033384 | 6.48E-17 | 1.86E-16 |
| *CMTM1* | 0.373632 | 0.9231 | 1.304869 | 7.55E-19 | 2.55E-18 |
| *SAPCD2* | 0.311422 | 4.735694 | 3.926635 | 1.01E-32 | 2.86E-30 |
| *TPX2* | 1.235213 | 17.74154 | 3.8443 | 8.70E-31 | 2.43E-29 |
| *ANP32E* | 14.96022 | 37.29645 | 1.317907 | 3.24E-18 | 1.04E-17 |
| *SAMD10* | 1.550415 | 5.555407 | 1.841238 | 2.65E-27 | 2.42E-26 |
| *KIFC2* | 1.321902 | 4.899878 | 1.890131 | 5.76E-16 | 1.55E-15 |
| *ANOS1* | 31.91014 | 5.039408 | -2.66269 | 2.55E-31 | 1.04E-29 |
| *KIFC1* | 1.115009 | 8.390485 | 2.911699 | 4.08E-28 | 4.29E-27 |
| *ANO9* | 1.925042 | 6.472267 | 1.749381 | 3.11E-17 | 9.19E-17 |
| *TPSB2* | 35.88981 | 10.59231 | -1.76056 | 1.07E-18 | 3.57E-18 |
| *OLR1* | 62.29078 | 12.04446 | -2.37065 | 4.55E-29 | 5.99E-28 |
| *CLSPN* | 0.20653 | 1.601138 | 2.954675 | 2.13E-26 | 1.63E-25 |
| *ANO7* | 0.16254 | 0.628948 | 1.952147 | 5.96E-26 | 4.22E-25 |
| *TPSAB1* | 31.41418 | 10.07572 | -1.64053 | 1.84E-22 | 8.88E-22 |
| *KIF4A* | 0.301482 | 4.90041 | 4.02276 | 2.17E-31 | 9.93E-30 |
| *CLPTM1L* | 21.52418 | 46.13747 | 1.09998 | 1.17E-21 | 5.16E-21 |
| *SAC3D1* | 3.087264 | 6.577884 | 1.091295 | 2.31E-20 | 8.85E-20 |
| *KIF3C* | 1.427602 | 3.505969 | 1.29622 | 4.99E-08 | 8.03E-08 |
| *CLPSL2* | 0.020744 | 0.985265 | 5.569767 | 2.90E-22 | 1.36E-21 |
| *ANLN* | 0.549268 | 8.975373 | 4.03039 | 1.62E-31 | 8.42E-30 |
| *KIF2C* | 0.534658 | 7.212123 | 3.753736 | 8.37E-32 | 6.08E-30 |
| *FOXA1* | 7.412915 | 22.37774 | 1.593951 | 9.17E-16 | 2.41E-15 |
| *S1PR1* | 52.55097 | 7.907103 | -2.7325 | 2.27E-32 | 3.48E-30 |
| *OIP5* | 0.40866 | 2.354846 | 2.52666 | 8.69E-28 | 8.54E-27 |
| *KIF26B* | 0.374664 | 3.622623 | 3.273364 | 3.72E-29 | 4.98E-28 |
| *TPI1* | 91.29131 | 218.31 | 1.257829 | 2.49E-26 | 1.88E-25 |
| *S100B* | 2.815052 | 7.719893 | 1.455419 | 0.000361 | 0.000463 |
| *FOSB* | 160.3277 | 18.97419 | -3.07891 | 6.60E-18 | 2.04E-17 |
| *TPBG* | 1.833605 | 6.795701 | 1.88994 | 2.60E-25 | 1.71E-24 |
| *OGDHL* | 0.055035 | 0.716462 | 3.702475 | 1.58E-09 | 2.79E-09 |
| *KIF23* | 0.477465 | 3.237003 | 2.761193 | 4.51E-28 | 4.68E-27 |
| *S100A5* | 0.321265 | 1.325968 | 2.04521 | 0.003581 | 0.004305 |
| *ANKRD34B* | 0.018555 | 0.73059 | 5.29916 | 3.48E-12 | 7.36E-12 |
| *S100A4* | 374.906 | 186.0135 | -1.01112 | 3.14E-19 | 1.10E-18 |
| *KIF20A* | 0.530265 | 5.464951 | 3.365423 | 1.48E-31 | 8.30E-30 |
| *S100A12* | 5.48208 | 1.495843 | -1.87376 | 7.02E-19 | 2.37E-18 |
| *KIF1A* | 0.060057 | 3.403376 | 5.824494 | 0.009077 | 0.010614 |
| *FLVCR2* | 7.77271 | 3.609683 | -1.10655 | 5.09E-23 | 2.60E-22 |
| *CLIC6* | 15.95462 | 42.08498 | 1.399332 | 0.000179 | 0.000235 |
| *KIF18B* | 0.203651 | 3.120287 | 3.93751 | 1.55E-30 | 3.57E-29 |
| *FLT4* | 5.601574 | 2.359157 | -1.24756 | 1.02E-19 | 3.73E-19 |
| *OCIAD2* | 9.669101 | 48.07226 | 2.313751 | 2.27E-31 | 9.99E-30 |
| *KIF18A* | 0.583007 | 1.747159 | 1.583426 | 3.51E-20 | 1.32E-19 |
| *RXRG* | 2.498992 | 0.545313 | -2.19619 | 4.41E-26 | 3.19E-25 |
| *OAS3* | 8.381129 | 16.96725 | 1.017537 | 1.93E-08 | 3.18E-08 |
| *KIF15* | 0.23896 | 1.753274 | 2.875212 | 5.99E-29 | 7.63E-28 |
| *FLI1* | 8.259651 | 2.841643 | -1.53936 | 1.56E-28 | 1.81E-27 |
| *KIF14* | 0.099597 | 1.576391 | 3.984374 | 3.11E-31 | 1.15E-29 |
| *FLAD1* | 5.006479 | 14.45154 | 1.529355 | 2.71E-31 | 1.08E-29 |
| *TOPBP1* | 3.71592 | 7.452494 | 1.004004 | 6.15E-18 | 1.92E-17 |
| *OAS1* | 8.197085 | 20.21066 | 1.301933 | 1.15E-10 | 2.18E-10 |
| *TOP2A* | 1.267839 | 21.38435 | 4.076112 | 2.11E-32 | 3.40E-30 |
| *RUNX2* | 0.757828 | 2.607757 | 1.782867 | 1.86E-22 | 8.94E-22 |
| *NXPH4* | 0.140613 | 2.838323 | 4.335238 | 1.45E-18 | 4.77E-18 |
| *FKBP4* | 10.18749 | 24.66043 | 1.2754 | 2.65E-21 | 1.11E-20 |
| *TOP1MT* | 2.596017 | 5.402912 | 1.057437 | 2.50E-19 | 8.82E-19 |
| *KIF11* | 0.815784 | 6.047658 | 2.890117 | 2.95E-31 | 1.13E-29 |
| *TONSL* | 0.724822 | 3.519624 | 2.279723 | 1.59E-29 | 2.36E-28 |
| *FIBIN* | 18.48657 | 4.591351 | -2.00949 | 1.91E-24 | 1.13E-23 |
| *NUSAP1* | 1.712763 | 11.40345 | 2.735073 | 3.66E-30 | 7.34E-29 |
| *FHL2* | 1.929805 | 10.42743 | 2.433857 | 7.08E-22 | 3.18E-21 |
| *CLEC1A* | 4.364527 | 0.94791 | -2.203 | 2.20E-31 | 9.93E-30 |
| *RTKN2* | 29.89797 | 1.959317 | -3.93162 | 3.68E-32 | 4.12E-30 |
| *NUP85* | 4.636014 | 9.452584 | 1.027824 | 3.66E-27 | 3.22E-26 |
| *FGR* | 25.8864 | 6.45257 | -2.00425 | 4.32E-30 | 8.42E-29 |
| *TNFSF4* | 0.526542 | 1.391529 | 1.40205 | 1.40E-12 | 3.01E-12 |
| *KHDC4* | 3.559573 | 10.84487 | 1.607236 | 4.92E-26 | 3.53E-25 |
| *CLEC14A* | 49.64305 | 10.75172 | -2.20702 | 1.38E-30 | 3.34E-29 |
| *NUP210* | 5.450376 | 13.24998 | 1.281562 | 5.56E-17 | 1.61E-16 |
| *KHDC1L* | 0.013682 | 0.664778 | 5.602548 | 8.70E-09 | 1.46E-08 |
| *FGFR4* | 16.16333 | 3.061205 | -2.40055 | 7.04E-29 | 8.71E-28 |
| *NUP155* | 2.373883 | 6.853479 | 1.529588 | 1.19E-28 | 1.41E-27 |
| *FGFBP2* | 5.251348 | 0.71186 | -2.88302 | 1.78E-28 | 2.02E-27 |
| *CLDN9* | 0.806066 | 4.140753 | 2.360923 | 3.07E-05 | 4.22E-05 |
| *AMH* | 0.152588 | 0.538771 | 1.820032 | 0.042173 | 0.046993 |
| *CLDN5* | 47.22884 | 7.395729 | -2.6749 | 1.51E-30 | 3.52E-29 |
| *TNFSF13* | 20.27482 | 9.501594 | -1.09345 | 4.16E-27 | 3.61E-26 |
| *NUF2* | 0.288405 | 4.233179 | 3.875573 | 2.22E-31 | 9.93E-30 |
| *ALPL* | 86.22954 | 39.873 | -1.11277 | 9.27E-17 | 2.63E-16 |
| *TNFRSF9* | 0.457801 | 1.343434 | 1.553134 | 1.50E-10 | 2.81E-10 |
| *RRM2* | 1.120227 | 11.8647 | 3.404813 | 1.17E-29 | 1.81E-28 |
| *NUDT8* | 2.276467 | 6.182302 | 1.441348 | 1.85E-20 | 7.16E-20 |
| *CLDN11* | 2.171393 | 0.993597 | -1.12789 | 1.63E-21 | 7.02E-21 |
| *NUDT1* | 3.478984 | 7.521771 | 1.112406 | 3.14E-19 | 1.10E-18 |
| *ALOX5AP* | 88.78344 | 25.67442 | -1.78996 | 1.08E-25 | 7.40E-25 |
| *RPUSD1* | 4.512718 | 9.501846 | 1.074211 | 2.81E-23 | 1.47E-22 |
| *KCP* | 0.131083 | 0.612674 | 2.224634 | 1.71E-07 | 2.68E-07 |
| *FGD5* | 11.87256 | 2.671211 | -2.15206 | 2.17E-31 | 9.93E-30 |
| *ALOX5* | 46.25472 | 14.17334 | -1.70642 | 4.96E-27 | 4.27E-26 |
| *TNFRSF25* | 1.026244 | 4.031503 | 1.973944 | 2.08E-18 | 6.75E-18 |
| *ALOX15* | 9.771303 | 2.355191 | -2.05271 | 4.25E-12 | 8.90E-12 |
| *CKS2* | 21.01651 | 49.21363 | 1.227535 | 4.79E-12 | 1.00E-11 |
| *ALKBH6* | 0.396213 | 0.865731 | 1.127643 | 2.00E-13 | 4.56E-13 |
| *FEZ1* | 5.028548 | 1.218557 | -2.04497 | 6.36E-31 | 1.93E-29 |
| *CKS1B* | 3.709295 | 13.60062 | 1.874456 | 1.57E-27 | 1.50E-26 |
| *TNFRSF18* | 0.973393 | 6.601404 | 2.761679 | 1.01E-21 | 4.48E-21 |
| *KCNQ3* | 0.698866 | 3.316988 | 2.246786 | 8.33E-16 | 2.20E-15 |
| *ALG8* | 6.035788 | 12.72777 | 1.076365 | 1.63E-29 | 2.40E-28 |
| *TNFRSF17* | 0.979781 | 3.872548 | 1.982752 | 2.80E-11 | 5.55E-11 |
| *CKAP4* | 19.23489 | 44.33288 | 1.204651 | 5.47E-23 | 2.78E-22 |
| *ALG1L* | 0.811794 | 8.494894 | 3.38741 | 5.78E-19 | 1.98E-18 |
| *FERMT1* | 0.60846 | 6.263125 | 3.363648 | 8.70E-30 | 1.41E-28 |
| *CKAP2L* | 0.223957 | 2.313368 | 3.368698 | 5.47E-30 | 9.76E-29 |
| *NT5DC3* | 1.016785 | 2.116368 | 1.057577 | 1.55E-13 | 3.55E-13 |
| *FEN1* | 4.29346 | 14.63181 | 1.768896 | 3.24E-28 | 3.44E-27 |
| *CKAP2* | 2.632768 | 6.539473 | 1.312594 | 2.56E-21 | 1.08E-20 |
| *ALDOA* | 95.59709 | 239.0571 | 1.322316 | 5.12E-25 | 3.23E-24 |
| *TNFRSF11A* | 0.493682 | 1.266439 | 1.359125 | 4.55E-12 | 9.52E-12 |
| *KCNK5* | 5.909758 | 18.39504 | 1.638146 | 3.48E-13 | 7.85E-13 |
| *CIT* | 2.506077 | 11.29708 | 2.172448 | 3.24E-10 | 5.96E-10 |
| *ALDH1B1* | 6.122095 | 14.50188 | 1.244142 | 9.09E-21 | 3.65E-20 |
| *NSUN5* | 4.589599 | 9.864181 | 1.103831 | 3.58E-25 | 2.30E-24 |
| *KCNJ15* | 13.30577 | 5.751971 | -1.20992 | 5.73E-18 | 1.79E-17 |
| *CIP2A* | 0.529282 | 2.366309 | 2.160531 | 2.05E-22 | 9.81E-22 |
| *RPL39L* | 2.875973 | 16.26846 | 2.499955 | 2.51E-22 | 1.20E-21 |
| *NSMF* | 4.661073 | 12.01807 | 1.366471 | 8.31E-24 | 4.63E-23 |
| *FCRLA* | 0.68517 | 1.463668 | 1.095054 | 7.15E-10 | 1.29E-09 |
| *CHTF18* | 0.838807 | 3.163968 | 1.915324 | 1.94E-23 | 1.03E-22 |
| *TMSB15A* | 3.837132 | 1.345977 | -1.51137 | 6.53E-19 | 2.22E-18 |
| *AKR1E2* | 0.576382 | 1.551608 | 1.428666 | 8.75E-09 | 1.47E-08 |
| *FCRL5* | 0.215095 | 1.367965 | 2.668985 | 6.63E-17 | 1.90E-16 |
| *CHST15* | 5.948602 | 13.40742 | 1.172409 | 4.42E-17 | 1.30E-16 |
| *AKR1B10* | 0.289059 | 48.66904 | 7.395498 | 1.21E-12 | 2.64E-12 |
| *ROBO4* | 20.7051 | 3.077157 | -2.75032 | 7.60E-32 | 5.67E-30 |
| *FCRL2* | 0.218171 | 0.896969 | 2.039597 | 1.04E-10 | 2.00E-10 |
| *NRIP3* | 0.725314 | 2.775577 | 1.93611 | 8.08E-09 | 1.36E-08 |
| *CHRNA5* | 0.111232 | 1.538414 | 3.789794 | 3.26E-26 | 2.40E-25 |
| *RNFT2* | 0.217828 | 1.131264 | 2.376675 | 1.60E-22 | 7.73E-22 |
| *FCN1* | 8.499475 | 2.800633 | -1.60162 | 5.83E-19 | 1.99E-18 |
| *RNF43* | 2.286034 | 5.960607 | 1.382612 | 1.97E-16 | 5.46E-16 |
| *AIM2* | 0.77235 | 5.994536 | 2.956322 | 1.06E-13 | 2.46E-13 |
| *FCGR3B* | 4.17588 | 0.903162 | -2.20902 | 1.67E-17 | 5.02E-17 |
| *CHML* | 2.403452 | 6.784384 | 1.49711 | 7.51E-14 | 1.77E-13 |
| *RNF207* | 0.692238 | 2.20656 | 1.672459 | 5.76E-16 | 1.55E-15 |
| *FCGR3A* | 82.79638 | 39.7959 | -1.05695 | 1.85E-17 | 5.54E-17 |
| *CHL1* | 1.043054 | 3.377483 | 1.695135 | 0.002965 | 0.003582 |
| *TMEM63C* | 0.110122 | 2.173266 | 4.302692 | 5.33E-20 | 1.98E-19 |
| *KBTBD12* | 0.161771 | 0.795463 | 2.297837 | 0.001418 | 0.001745 |
| *NR4A3* | 10.51635 | 2.479258 | -2.08465 | 1.61E-15 | 4.16E-15 |
| *CHI3L2* | 16.45547 | 5.827821 | -1.49754 | 6.43E-19 | 2.19E-18 |
| *RNF183* | 0.048813 | 1.02646 | 4.394282 | 6.68E-14 | 1.58E-13 |
| *KAZALD1* | 1.45032 | 2.930266 | 1.01466 | 9.24E-08 | 1.47E-07 |
| *CHI3L1* | 21.891 | 62.13519 | 1.505073 | 0.001259 | 0.001555 |
| *NR2F6* | 11.22092 | 23.83098 | 1.086647 | 1.14E-20 | 4.55E-20 |
| *CHEK2* | 1.241805 | 3.859698 | 1.63605 | 3.72E-26 | 2.73E-25 |
| *AHNAK* | 122.6824 | 35.40462 | -1.79292 | 9.51E-26 | 6.60E-25 |
| *RNF144B* | 20.80676 | 7.164831 | -1.53805 | 5.63E-29 | 7.28E-28 |
| *CHEK1* | 0.535895 | 2.892017 | 2.432054 | 4.74E-30 | 8.83E-29 |
| *FCER1G* | 124.5139 | 58.42805 | -1.09157 | 5.99E-20 | 2.21E-19 |
| *KANK2* | 32.4618 | 11.30212 | -1.52215 | 1.17E-29 | 1.81E-28 |
| *CHD1L* | 9.727515 | 20.4061 | 1.068857 | 1.69E-26 | 1.31E-25 |
| *CHAF1B* | 0.659029 | 2.86079 | 2.117999 | 1.41E-31 | 8.03E-30 |
| *NQO1* | 11.71607 | 101.1012 | 3.109239 | 6.41E-21 | 2.61E-20 |
| *CHAF1A* | 2.06491 | 4.637397 | 1.167236 | 4.25E-19 | 1.47E-18 |
| *RNASEH2A* | 4.344221 | 11.77988 | 1.439155 | 6.32E-23 | 3.17E-22 |
| *NPTX2* | 0.446391 | 2.989534 | 2.743541 | 6.19E-08 | 9.94E-08 |
| *FBXO5* | 1.298425 | 2.67808 | 1.044436 | 9.03E-14 | 2.11E-13 |
| *CHAD* | 0.726143 | 3.154158 | 2.11893 | 0.002346 | 0.00286 |
| *NPR1* | 15.98451 | 2.84166 | -2.49187 | 1.29E-30 | 3.27E-29 |
| *FBXO41* | 0.558548 | 2.053627 | 1.87842 | 6.14E-21 | 2.51E-20 |
| *CFTR* | 5.311514 | 2.407005 | -1.14188 | 1.86E-16 | 5.16E-16 |
| *AFF3* | 3.001342 | 0.61566 | -2.2854 | 1.24E-27 | 1.20E-26 |
| *TMEM198* | 0.764808 | 1.593828 | 1.059326 | 3.99E-07 | 6.13E-07 |
| *CFP* | 3.175685 | 0.80592 | -1.97836 | 3.13E-29 | 4.23E-28 |
| *TMEM178A* | 2.925225 | 1.406187 | -1.05676 | 3.76E-19 | 1.31E-18 |
| *NPIPB15* | 0.717824 | 1.891886 | 1.398122 | 0.000592 | 0.00075 |
| *AFAP1L1* | 8.607494 | 2.838648 | -1.60039 | 1.39E-26 | 1.10E-25 |
| *RMI2* | 1.102625 | 6.234727 | 2.499384 | 4.94E-31 | 1.58E-29 |
| *TMEM156* | 0.507973 | 3.043863 | 2.583079 | 2.68E-18 | 8.64E-18 |
| *ITM2A* | 35.29543 | 9.390546 | -1.9102 | 4.42E-28 | 4.63E-27 |
| *FBXL6* | 3.117646 | 7.645501 | 1.294154 | 9.53E-22 | 4.22E-21 |
| *ADRB2* | 12.85155 | 1.681538 | -2.93409 | 9.40E-33 | 2.86E-30 |
| *RIPPLY3* | 0.357151 | 1.025863 | 1.52223 | 0.021146 | 0.024086 |
| *CERS6* | 2.40516 | 5.389922 | 1.164132 | 6.92E-18 | 2.13E-17 |
| *FBXL16* | 1.212959 | 3.294781 | 1.441652 | 0.011278 | 0.013087 |
| *ADGRL4* | 11.27431 | 5.067666 | -1.15365 | 4.01E-19 | 1.39E-18 |
| *TMEM132A* | 1.976008 | 8.814277 | 2.157253 | 4.77E-22 | 2.20E-21 |
| *ITGB8* | 1.049085 | 3.330977 | 1.666814 | 0.00621 | 0.00735 |
| *FBN3* | 1.059642 | 0.513391 | -1.04545 | 7.51E-15 | 1.87E-14 |
| *RILPL2* | 9.664597 | 4.209285 | -1.19913 | 8.17E-31 | 2.33E-29 |
| *NOXA1* | 3.391926 | 9.068375 | 1.418739 | 1.22E-12 | 2.65E-12 |
| *FBN2* | 0.108564 | 0.621187 | 2.516487 | 0.027484 | 0.031039 |
| *CEP72* | 0.951267 | 2.63339 | 1.468999 | 2.73E-23 | 1.44E-22 |
| *ADGRG1* | 7.919642 | 22.60702 | 1.513264 | 1.55E-17 | 4.66E-17 |
| *NOX4* | 0.345453 | 0.954574 | 1.466368 | 1.21E-11 | 2.46E-11 |
| *ITGB4* | 10.9322 | 36.43665 | 1.736806 | 1.21E-10 | 2.29E-10 |
| *RHPN1* | 2.348083 | 9.534287 | 2.021642 | 3.60E-20 | 1.35E-19 |
| *FBLN5* | 33.10079 | 10.18653 | -1.7002 | 1.47E-28 | 1.73E-27 |
| *ADGRE5* | 51.97937 | 23.4383 | -1.14907 | 2.03E-24 | 1.19E-23 |
| *NOX1* | 0.303717 | 0.882442 | 1.538776 | 9.17E-16 | 2.41E-15 |
| *FBLN1* | 55.60627 | 23.09533 | -1.26765 | 1.19E-18 | 3.92E-18 |
| *CEP55* | 0.717329 | 7.873441 | 3.456288 | 1.39E-30 | 3.35E-29 |
| *ADGRE3* | 2.145396 | 0.322897 | -2.7321 | 1.28E-26 | 1.02E-25 |
| *RHOJ* | 9.890522 | 2.931987 | -1.75417 | 6.94E-30 | 1.17E-28 |
| *CEP131* | 2.972676 | 6.082768 | 1.032966 | 2.68E-14 | 6.49E-14 |
| *ADGRE1* | 3.777243 | 0.667732 | -2.49999 | 1.83E-26 | 1.40E-25 |
| *ITGAL* | 13.57449 | 6.51129 | -1.05988 | 6.20E-18 | 1.93E-17 |
| *CENPW* | 3.183869 | 9.849802 | 1.629314 | 4.59E-15 | 1.16E-14 |
| *ADGRB2* | 0.314863 | 0.989889 | 1.652543 | 6.17E-12 | 1.28E-11 |
| *CENPO* | 1.107323 | 2.547348 | 1.20192 | 3.27E-22 | 1.53E-21 |
| *ADGRB1* | 0.185727 | 1.59457 | 3.101915 | 2.38E-05 | 3.29E-05 |
| *CENPN* | 1.302826 | 3.250454 | 1.318997 | 1.27E-18 | 4.21E-18 |
| *TM6SF1* | 3.839932 | 1.577262 | -1.28366 | 3.71E-24 | 2.14E-23 |
| *FAP* | 0.664083 | 3.549354 | 2.418121 | 1.24E-23 | 6.79E-23 |
| *CENPM* | 1.366318 | 5.094735 | 1.898713 | 8.79E-21 | 3.53E-20 |
| *RHBDD3* | 3.960794 | 8.155115 | 1.041916 | 1.26E-19 | 4.59E-19 |
| *ITGA11* | 0.571167 | 4.47688 | 2.970509 | 3.02E-23 | 1.57E-22 |
| *FANCI* | 0.974966 | 4.34152 | 2.154777 | 1.29E-30 | 3.27E-29 |
| *CENPL* | 0.682234 | 2.055585 | 1.59121 | 3.75E-29 | 5.01E-28 |
| *FANCG* | 2.084857 | 5.341486 | 1.357292 | 7.70E-27 | 6.45E-26 |
| *CENPK* | 0.346978 | 1.829643 | 2.398647 | 9.44E-27 | 7.76E-26 |
| *ADCY4* | 3.584324 | 1.099103 | -1.70537 | 1.33E-25 | 9.03E-25 |
| *CENPI* | 0.186008 | 1.427883 | 2.940445 | 1.99E-27 | 1.87E-26 |
| *RGS20* | 0.08949 | 0.720983 | 3.010173 | 1.03E-13 | 2.40E-13 |
| *NOL3* | 7.161345 | 14.50172 | 1.017921 | 2.58E-15 | 6.58E-15 |
| *FANCD2* | 0.637534 | 2.093408 | 1.715279 | 5.56E-28 | 5.65E-27 |
| *CENPH* | 1.384321 | 4.16234 | 1.588216 | 2.37E-26 | 1.79E-25 |
| *ADCK5* | 1.613244 | 5.426068 | 1.749943 | 2.67E-25 | 1.75E-24 |
| *CENPF* | 0.396683 | 5.207733 | 3.714596 | 8.00E-31 | 2.33E-29 |
| *TLR8* | 5.792703 | 1.942092 | -1.57663 | 2.58E-22 | 1.22E-21 |
| *RGS17* | 0.115002 | 1.766264 | 3.94097 | 1.90E-26 | 1.46E-25 |
| *ISG15* | 34.43242 | 107.6985 | 1.645159 | 8.01E-10 | 1.44E-09 |
| *FANCA* | 0.439054 | 1.806334 | 2.040595 | 1.53E-28 | 1.78E-27 |
| *CENPE* | 0.207598 | 1.540663 | 2.891684 | 1.99E-27 | 1.87E-26 |
| *CENPA* | 0.257235 | 3.618955 | 3.814414 | 1.41E-29 | 2.11E-28 |
| *FAM90A1* | 0.270764 | 0.851071 | 1.652244 | 1.86E-06 | 2.75E-06 |
| *ADARB1* | 7.351807 | 2.38411 | -1.62465 | 3.16E-26 | 2.35E-25 |
| *TLR10* | 0.466098 | 1.125859 | 1.272321 | 1.59E-08 | 2.63E-08 |
| *CEMIP* | 2.259292 | 10.23219 | 2.179172 | 7.23E-10 | 1.30E-09 |
| *CELSR3* | 0.123226 | 1.466274 | 3.572779 | 3.39E-29 | 4.56E-28 |
| *CELF3* | 0.029789 | 0.836914 | 4.812207 | 0.00022 | 0.000286 |
| *TLCD1* | 4.797983 | 17.94565 | 1.903134 | 9.08E-27 | 7.48E-26 |
| *IRF7* | 8.281184 | 21.37489 | 1.368008 | 2.41E-21 | 1.02E-20 |
| *CEL* | 0.188049 | 1.677859 | 3.157441 | 0.000826 | 0.001034 |
| *RGCC* | 303.6026 | 44.18656 | -2.7805 | 1.05E-32 | 2.86E-30 |
| *IRF4* | 1.27338 | 2.803022 | 1.13832 | 0.000813 | 0.001019 |
| *TK1* | 5.027055 | 35.35409 | 2.814092 | 5.79E-28 | 5.84E-27 |
| *CEACAM6* | 207.6236 | 603.8682 | 1.540263 | 5.15E-07 | 7.83E-07 |
| *ADAMDEC1* | 1.038593 | 5.816702 | 2.48557 | 1.32E-13 | 3.05E-13 |
| *RFC5* | 2.878918 | 6.063575 | 1.074642 | 2.91E-23 | 1.52E-22 |
| *IQGAP3* | 0.369073 | 5.130486 | 3.797116 | 7.37E-32 | 5.67E-30 |
| *ADAM8* | 3.255456 | 15.45728 | 2.247355 | 5.03E-22 | 2.32E-21 |
| *RFC4* | 1.800629 | 6.928636 | 1.94407 | 3.20E-30 | 6.64E-29 |
| *CDT1* | 0.723608 | 5.517472 | 2.930728 | 1.04E-27 | 1.01E-26 |
| *TIMM8A* | 1.250724 | 3.20881 | 1.359275 | 5.76E-30 | 1.02E-28 |
| *RFC3* | 2.600432 | 5.545089 | 1.09246 | 5.91E-18 | 1.85E-17 |
| *FAM43A* | 7.241647 | 3.453211 | -1.06838 | 3.62E-15 | 9.18E-15 |
| *CDKN3* | 0.673503 | 5.55535 | 3.044122 | 2.38E-27 | 2.20E-26 |
| *IPCEF1* | 0.649087 | 1.685075 | 1.37633 | 0.005502 | 0.006544 |
| *FAM3C* | 8.519867 | 22.42759 | 1.396372 | 1.45E-20 | 5.67E-20 |
| *ADAM28* | 0.799511 | 3.985412 | 2.317539 | 2.97E-22 | 1.39E-21 |
| *RETREG1* | 7.195506 | 3.022842 | -1.25119 | 1.67E-20 | 6.51E-20 |
| *INTS8* | 2.562895 | 6.467699 | 1.335478 | 2.13E-30 | 4.63E-29 |
| *CDKN2A* | 0.513272 | 6.642782 | 3.693992 | 1.11E-13 | 2.57E-13 |
| *TIMELESS* | 2.379692 | 8.805811 | 1.887681 | 2.52E-30 | 5.31E-29 |
| *INTS7* | 3.348325 | 6.903911 | 1.043975 | 5.80E-27 | 4.95E-26 |
| *FAM24B* | 0.588577 | 1.576937 | 1.421823 | 4.36E-20 | 1.63E-19 |
| *RET* | 0.236939 | 1.972846 | 3.057694 | 0.013818 | 0.015951 |
| *ADAM15* | 17.68644 | 37.22162 | 1.073497 | 5.88E-19 | 2.00E-18 |
| *TIGIT* | 0.665666 | 1.699044 | 1.351854 | 3.47E-10 | 6.38E-10 |
| *INSYN2A* | 0.089918 | 0.558625 | 2.635203 | 2.49E-06 | 3.64E-06 |
| *CDK5RAP3* | 9.261264 | 19.732 | 1.091256 | 3.85E-17 | 1.13E-16 |
| *ADAM12* | 0.323864 | 3.295097 | 3.346858 | 7.62E-23 | 3.79E-22 |
| *ACVRL1* | 36.98182 | 6.668024 | -2.47148 | 4.32E-32 | 4.19E-30 |
| *TIGD3* | 0.190526 | 0.598636 | 1.651687 | 5.91E-17 | 1.70E-16 |
| *CDK5R1* | 0.480812 | 1.623706 | 1.755746 | 6.24E-20 | 2.30E-19 |
| *TIE1* | 14.80812 | 4.029716 | -1.87764 | 1.01E-29 | 1.60E-28 |
| *TICRR* | 0.087765 | 0.765546 | 3.124763 | 1.99E-28 | 2.22E-27 |
| *THY1* | 6.023386 | 16.11914 | 1.420128 | 3.02E-14 | 7.28E-14 |
| *FAM178B* | 0.116894 | 0.93185 | 2.994897 | 5.91E-18 | 1.85E-17 |
| *NHLRC1* | 1.269634 | 2.56892 | 1.016749 | 1.13E-11 | 2.30E-11 |
| *INPP4B* | 1.147797 | 2.445732 | 1.091398 | 3.56E-06 | 5.16E-06 |
| *ACTL8* | 0.001705 | 0.568534 | 8.381357 | 9.75E-10 | 1.73E-09 |
| *FAM171A2* | 0.418428 | 1.817263 | 2.118717 | 5.65E-12 | 1.17E-11 |
| *ACTL6A* | 8.038317 | 17.27424 | 1.103657 | 1.56E-25 | 1.05E-24 |
| *REG4* | 0.010939 | 15.83585 | 10.49947 | 1.59E-05 | 2.23E-05 |
| *INKA2* | 1.800913 | 0.660221 | -1.44771 | 1.00E-29 | 1.60E-28 |
| *ACTL10* | 0.557316 | 1.360711 | 1.287794 | 4.87E-10 | 8.86E-10 |
| *RECQL4* | 0.705719 | 6.336777 | 3.166584 | 1.98E-31 | 9.91E-30 |
| *CDK1* | 1.559508 | 9.759314 | 2.645688 | 3.42E-27 | 3.02E-26 |
| *NFE2L3* | 4.142549 | 12.13706 | 1.550828 | 9.13E-17 | 2.59E-16 |
| *INA* | 0.049985 | 1.317756 | 4.720452 | 7.83E-10 | 1.41E-09 |
| *FAM136A* | 6.670386 | 16.56066 | 1.311918 | 9.00E-33 | 2.86E-30 |
| *ACSS3* | 2.395515 | 0.858073 | -1.48116 | 2.66E-26 | 2.00E-25 |
| *NFATC4* | 1.975681 | 4.957471 | 1.327254 | 4.58E-16 | 1.24E-15 |
| *FAM133A* | 0.031257 | 1.569954 | 5.650389 | 0.001066 | 0.001325 |
| *FAM124B* | 1.630616 | 0.494873 | -1.72029 | 1.68E-23 | 9.10E-23 |
| *CDH5* | 43.47411 | 7.952209 | -2.45073 | 9.61E-32 | 6.35E-30 |
| *THBS2* | 4.433527 | 36.99781 | 3.060913 | 2.53E-25 | 1.67E-24 |
| *RCOR2* | 0.282288 | 2.108835 | 2.901204 | 7.60E-18 | 2.33E-17 |
| *ILF2* | 47.44773 | 109.2724 | 1.203519 | 1.51E-28 | 1.77E-27 |
| *RCN3* | 6.000123 | 22.18183 | 1.886315 | 1.05E-15 | 2.73E-15 |
| *NFATC1* | 3.947911 | 1.958811 | -1.01111 | 8.33E-17 | 2.37E-16 |
| *FAM111B* | 0.445517 | 3.559087 | 2.997954 | 1.09E-28 | 1.30E-27 |
| *CDH24* | 1.256913 | 4.011046 | 1.674094 | 1.17E-17 | 3.56E-17 |
| *ACP6* | 0.91972 | 2.519866 | 1.45408 | 6.11E-25 | 3.84E-24 |
| *THBD* | 54.57155 | 10.84783 | -2.33074 | 5.31E-30 | 9.58E-29 |
| *RCC2* | 20.19849 | 44.55302 | 1.141276 | 1.61E-26 | 1.25E-25 |
| *CDH2* | 0.41401 | 1.815395 | 2.132544 | 0.026983 | 0.030496 |
| *ACP5* | 132.7722 | 41.05215 | -1.69342 | 2.76E-27 | 2.48E-26 |
| *RCC1* | 4.910618 | 18.47689 | 1.911745 | 8.25E-33 | 2.86E-30 |
| *IL7R* | 38.18495 | 10.28555 | -1.89239 | 1.84E-23 | 9.89E-23 |
| *CDH1* | 35.14423 | 79.79412 | 1.182995 | 4.59E-20 | 1.71E-19 |
| *ACHE* | 0.862498 | 5.165416 | 2.582291 | 2.88E-07 | 4.45E-07 |
| *CDCA8* | 0.947233 | 8.775097 | 3.211623 | 1.65E-31 | 8.42E-30 |
| *ACBD7* | 0.321432 | 0.721615 | 1.166716 | 9.87E-06 | 1.40E-05 |
| *FABP5* | 25.35651 | 7.718577 | -1.71595 | 1.18E-25 | 8.10E-25 |
| *CDCA7* | 0.90617 | 7.522252 | 3.053312 | 3.48E-25 | 2.25E-24 |
| *NETO2* | 1.385941 | 3.983333 | 1.52311 | 5.91E-17 | 1.70E-16 |
| *FABP4* | 107.7517 | 3.960279 | -4.76596 | 8.90E-33 | 2.86E-30 |
| *CDCA5* | 0.728917 | 6.539719 | 3.165403 | 2.19E-29 | 3.06E-28 |
| *TGFBR2* | 115.9618 | 39.50553 | -1.55352 | 9.11E-32 | 6.35E-30 |
| *RBMS3* | 4.768581 | 1.869252 | -1.3511 | 4.53E-25 | 2.88E-24 |
| *IL4I1* | 1.798208 | 7.689022 | 2.09624 | 5.76E-16 | 1.55E-15 |
| *CDCA4* | 2.855766 | 8.717651 | 1.610062 | 3.98E-26 | 2.90E-25 |
| *CDCA3* | 0.225419 | 2.188775 | 3.279444 | 3.76E-31 | 1.32E-29 |
| *ACADL* | 6.603539 | 0.815548 | -3.0174 | 6.69E-32 | 5.62E-30 |
| *NEK5* | 1.305836 | 0.475867 | -1.45634 | 1.15E-10 | 2.19E-10 |
| *IL3RA* | 19.54266 | 6.55744 | -1.57542 | 1.42E-29 | 2.12E-28 |
| *FAAP24* | 0.876971 | 2.32521 | 1.40676 | 1.68E-23 | 9.10E-23 |
| *CDCA2* | 0.191744 | 1.735359 | 3.177984 | 5.14E-30 | 9.41E-29 |
| *ACAD8* | 3.21075 | 8.9252 | 1.474974 | 7.37E-19 | 2.49E-18 |
| *IL37* | 0.132428 | 13.09671 | 6.627853 | 7.14E-13 | 1.58E-12 |
| *CDC7* | 1.22178 | 3.337412 | 1.449745 | 4.67E-18 | 1.47E-17 |
| *ACACB* | 2.35728 | 1.008217 | -1.22532 | 2.99E-20 | 1.14E-19 |
| *TFF2* | 0.017491 | 33.80501 | 10.9164 | 9.15E-06 | 1.30E-05 |
| *NEK2* | 0.366625 | 5.616045 | 3.937178 | 3.88E-31 | 1.34E-29 |
| *IL34* | 4.688876 | 2.054584 | -1.1904 | 5.05E-21 | 2.07E-20 |
| *F8* | 5.934268 | 1.868018 | -1.66756 | 3.90E-25 | 2.50E-24 |
| *CDC6* | 0.386968 | 4.752026 | 3.618257 | 2.47E-31 | 1.02E-29 |
| *TFF1* | 0.174817 | 80.34236 | 8.84417 | 4.38E-11 | 8.61E-11 |
| *NEIL3* | 0.087203 | 1.827769 | 4.389561 | 1.24E-30 | 3.22E-29 |
| *RBBP8NL* | 0.424943 | 2.004598 | 2.237972 | 3.25E-23 | 1.68E-22 |
| *F2RL3* | 5.989738 | 1.771486 | -1.75753 | 4.85E-12 | 1.01E-11 |
| *CDC45* | 0.406009 | 4.15314 | 3.354619 | 4.45E-30 | 8.52E-29 |
| *F2RL2* | 0.364203 | 0.906639 | 1.315784 | 0.002375 | 0.002893 |
| *CDC25C* | 0.112499 | 1.629943 | 3.856837 | 7.44E-32 | 5.67E-30 |
| *ABI3BP* | 13.86403 | 2.720714 | -2.34929 | 2.03E-30 | 4.46E-29 |
| *IL2RA* | 1.249014 | 3.659566 | 1.550883 | 6.71E-13 | 1.48E-12 |
| *F12* | 0.267405 | 2.071288 | 2.953431 | 1.58E-24 | 9.51E-24 |
| *CDC25A* | 0.247299 | 1.447831 | 2.549567 | 1.77E-26 | 1.37E-25 |
| *RASIP1* | 12.27942 | 2.64515 | -2.21482 | 8.09E-31 | 2.33E-29 |
| *NDUFS6* | 21.52446 | 45.31698 | 1.074075 | 8.49E-21 | 3.42E-20 |
| *CDC20* | 1.24206 | 19.04258 | 3.938422 | 2.15E-31 | 9.93E-30 |
| *RASGRP2* | 2.652816 | 1.268276 | -1.06466 | 1.09E-18 | 3.65E-18 |
| *EZH2* | 0.782538 | 5.176213 | 2.725665 | 1.41E-31 | 8.03E-30 |
| *NDUFAF6* | 1.507466 | 3.223517 | 1.09651 | 5.37E-20 | 2.00E-19 |
| *CDA* | 3.299273 | 19.26112 | 2.545472 | 9.47E-05 | 0.000126 |
| *ABHD11* | 8.087676 | 23.62862 | 1.546738 | 1.08E-23 | 5.97E-23 |
| *IL1RL1* | 9.922415 | 1.491551 | -2.73388 | 1.92E-23 | 1.03E-22 |
| *ABCG2* | 3.954676 | 1.051075 | -1.91169 | 3.36E-27 | 2.98E-26 |
| *CD93* | 59.62016 | 14.11108 | -2.07897 | 6.13E-30 | 1.07E-28 |
| *TESMIN* | 0.135429 | 1.028901 | 2.925498 | 5.38E-18 | 1.69E-17 |
| *TESC* | 3.488228 | 42.42654 | 3.604401 | 5.60E-08 | 8.99E-08 |
| *IL18R1* | 3.503218 | 1.342496 | -1.38376 | 2.35E-14 | 5.72E-14 |
| *NDC80* | 0.367021 | 3.715302 | 3.339543 | 2.39E-30 | 5.08E-29 |
| *EXOSC5* | 5.958078 | 14.71317 | 1.304189 | 3.25E-23 | 1.68E-22 |
| *ABCB9* | 0.282491 | 0.766444 | 1.439974 | 1.26E-22 | 6.16E-22 |
| *TEKT2* | 5.838338 | 1.968814 | -1.56823 | 1.35E-07 | 2.13E-07 |
| *TEK* | 21.62083 | 2.449167 | -3.14206 | 1.42E-32 | 2.94E-30 |
| *CD79A* | 7.8141 | 22.37414 | 1.517681 | 2.90E-12 | 6.16E-12 |
| *TEDC2* | 0.188797 | 2.39082 | 3.662598 | 3.56E-32 | 4.12E-30 |
| *RANBP1* | 6.798576 | 14.41317 | 1.084083 | 1.03E-25 | 7.09E-25 |
| *NCKAP1L* | 10.81911 | 5.085224 | -1.0892 | 5.07E-20 | 1.89E-19 |
| *EXO1* | 0.186808 | 2.596686 | 3.79704 | 3.60E-31 | 1.29E-29 |
| *AARD* | 3.232487 | 1.004794 | -1.68575 | 4.03E-23 | 2.07E-22 |
| *RAMP3* | 84.08874 | 9.520961 | -3.14273 | 5.64E-32 | 4.97E-30 |
| *NCF2* | 44.78803 | 15.51373 | -1.52957 | 4.18E-26 | 3.03E-25 |
| *RAMP2* | 82.12676 | 11.76472 | -2.80339 | 1.85E-32 | 3.17E-30 |
| *NCF1* | 3.511559 | 1.554217 | -1.17592 | 1.46E-14 | 3.58E-14 |
| *CD70* | 0.33715 | 0.887711 | 1.396699 | 2.60E-05 | 3.59E-05 |
| *RALGPS2* | 1.19995 | 4.25715 | 1.826913 | 7.17E-25 | 4.45E-24 |
| *EVI2B* | 28.46731 | 13.3547 | -1.09196 | 5.42E-20 | 2.01E-19 |
| *NCAPH* | 0.523839 | 4.738195 | 3.177142 | 1.37E-29 | 2.07E-28 |
| *NCAPG2* | 1.259738 | 4.942581 | 1.97214 | 4.56E-28 | 4.71E-27 |
| *ETV4* | 0.930833 | 14.52454 | 3.963827 | 7.68E-31 | 2.28E-29 |
| *CD68* | 3.434397 | 1.209457 | -1.5057 | 8.73E-22 | 3.88E-21 |
| *TDRD5* | 0.062673 | 0.947419 | 3.918097 | 3.98E-09 | 6.83E-09 |
| *NCAPG* | 0.326885 | 3.435043 | 3.393472 | 1.66E-29 | 2.43E-28 |
| *NCAPD2* | 6.449172 | 14.72603 | 1.191183 | 4.63E-18 | 1.46E-17 |
| *RADX* | 1.5449 | 0.543633 | -1.50681 | 3.80E-21 | 1.58E-20 |
| *ETS1* | 33.49288 | 16.45821 | -1.02505 | 2.34E-19 | 8.29E-19 |
| *RAD54L* | 0.196781 | 2.191693 | 3.47738 | 1.08E-30 | 2.92E-29 |
| *IGLL5* | 9.59852 | 60.10627 | 2.646632 | 1.05E-14 | 2.59E-14 |
| *RAD54B* | 0.156777 | 0.582894 | 1.894515 | 8.08E-27 | 6.73E-26 |
| *ESCO2* | 0.104689 | 0.741962 | 2.825234 | 2.20E-28 | 2.43E-27 |
| *RAD51AP1* | 0.902129 | 4.285659 | 2.248111 | 4.22E-24 | 2.41E-23 |
| *ESAM* | 58.30328 | 16.45005 | -1.82549 | 2.60E-29 | 3.59E-28 |
| *CD52* | 210.5565 | 44.47107 | -2.24327 | 6.90E-29 | 8.57E-28 |
| *NAXE* | 29.93308 | 68.58875 | 1.196231 | 3.45E-22 | 1.60E-21 |
| *TCF19* | 3.071091 | 7.798323 | 1.344413 | 4.47E-20 | 1.67E-19 |
| *RACGAP1* | 2.77111 | 8.79154 | 1.665652 | 2.86E-22 | 1.35E-21 |
| *ERICH5* | 1.217718 | 3.055986 | 1.327458 | 6.14E-05 | 8.29E-05 |
| *RAC3* | 1.875082 | 10.52677 | 2.489036 | 1.19E-23 | 6.51E-23 |
| *NAV1* | 1.442084 | 2.957053 | 1.036005 | 9.70E-11 | 1.86E-10 |
| *IGFBP3* | 16.95369 | 83.52385 | 2.300589 | 4.15E-19 | 1.43E-18 |
| *NATD1* | 6.910456 | 3.346138 | -1.04628 | 7.66E-22 | 3.42E-21 |
| *IGF2BP3* | 0.103474 | 1.901143 | 4.199528 | 1.29E-12 | 2.79E-12 |
| *ERG* | 9.136734 | 2.208982 | -2.0483 | 5.55E-31 | 1.72E-29 |
| *RAB6B* | 1.433418 | 2.944309 | 1.03847 | 8.37E-05 | 0.000112 |
| *ERCC6L* | 0.108555 | 1.221379 | 3.492009 | 1.13E-32 | 2.86E-30 |
| *IGF2BP1* | 0.011225 | 1.061195 | 6.56289 | 2.73E-11 | 5.44E-11 |
| *TBX6* | 0.502354 | 1.152582 | 1.198095 | 5.37E-10 | 9.74E-10 |
| *NARF* | 3.787218 | 9.860264 | 1.380488 | 2.54E-30 | 5.32E-29 |
| *ERBB2* | 16.34843 | 34.99061 | 1.097816 | 2.07E-14 | 5.06E-14 |
| *TBX2* | 15.11617 | 4.245049 | -1.83224 | 2.66E-26 | 2.00E-25 |
| *RAB3B* | 0.038223 | 0.785392 | 4.360911 | 4.08E-16 | 1.11E-15 |
| *TBRG4* | 5.13092 | 11.88956 | 1.212406 | 7.27E-28 | 7.24E-27 |
| *EPYC* | 0.017043 | 0.824927 | 5.597016 | 6.54E-16 | 1.75E-15 |
| *CD37* | 17.7838 | 8.871054 | -1.00339 | 1.58E-17 | 4.76E-17 |
| *RAB38* | 4.832669 | 10.47071 | 1.115467 | 1.34E-10 | 2.54E-10 |
| *NAALADL2* | 0.578435 | 1.491198 | 1.366246 | 2.75E-09 | 4.75E-09 |
| *IFNG* | 0.33131 | 0.908773 | 1.455736 | 0.004785 | 0.005706 |
| *CD36* | 20.17133 | 2.869619 | -2.81338 | 2.13E-30 | 4.63E-29 |
| *EPOP* | 0.779305 | 2.24388 | 1.525735 | 4.74E-17 | 1.38E-16 |
| *CD34* | 15.96743 | 5.43002 | -1.5561 | 7.05E-27 | 5.94E-26 |
| *RAB27B* | 4.107298 | 8.382795 | 1.029242 | 0.000787 | 0.000988 |
| *CD33* | 2.561932 | 1.03385 | -1.3092 | 1.27E-22 | 6.20E-22 |
| *EPHX4* | 0.278351 | 1.840126 | 2.72483 | 3.98E-18 | 1.26E-17 |
| *CD302* | 9.531553 | 4.175075 | -1.19091 | 6.86E-24 | 3.87E-23 |
| *RAB15* | 5.004882 | 14.4776 | 1.532414 | 2.45E-14 | 5.96E-14 |
| *CD300LF* | 13.84778 | 4.433484 | -1.64314 | 8.00E-27 | 6.69E-26 |
| *MZT2A* | 2.768463 | 6.757027 | 1.287304 | 5.26E-24 | 2.98E-23 |
| *EPHB3* | 2.755304 | 7.581288 | 1.460232 | 1.62E-13 | 3.72E-13 |
| *RAB11FIP1* | 31.42833 | 12.92186 | -1.28225 | 1.23E-21 | 5.37E-21 |
| *EPHB2* | 0.669444 | 3.575547 | 2.417129 | 5.56E-20 | 2.06E-19 |
| *CD300E* | 2.962795 | 1.254759 | -1.23955 | 2.12E-16 | 5.88E-16 |
| *TBC1D31* | 0.513779 | 1.197886 | 1.221271 | 1.82E-17 | 5.46E-17 |
| *MZB1* | 4.509928 | 21.14925 | 2.22943 | 3.72E-13 | 8.38E-13 |
| *EPHA1* | 2.329268 | 4.798682 | 1.042762 | 1.38E-13 | 3.17E-13 |
| *QPCT* | 2.878821 | 19.76489 | 2.77939 | 2.12E-09 | 3.71E-09 |
| *MYO7A* | 0.432974 | 1.896877 | 2.131273 | 2.76E-27 | 2.48E-26 |
| *CD27* | 2.87755 | 7.632332 | 1.407283 | 5.52E-11 | 1.08E-10 |
| *CD244* | 1.769258 | 0.786532 | -1.16957 | 6.78E-16 | 1.80E-15 |
| *PYCR3* | 2.724226 | 7.842162 | 1.525405 | 1.10E-25 | 7.52E-25 |
| *ID3* | 52.17126 | 25.04898 | -1.0585 | 5.12E-17 | 1.48E-16 |
| *ENPP3* | 0.570981 | 2.928309 | 2.358554 | 0.02655 | 0.03003 |
| *PYCR1* | 3.075311 | 44.82049 | 3.865354 | 1.18E-32 | 2.86E-30 |
| *MYL9* | 173.9653 | 57.13179 | -1.60643 | 3.55E-30 | 7.21E-29 |
| *PUS1* | 1.778315 | 4.354842 | 1.292109 | 1.30E-24 | 7.91E-24 |
| *TAL1* | 3.085335 | 0.481679 | -2.67928 | 2.39E-31 | 1.01E-29 |
| *ICAM2* | 9.175839 | 3.648665 | -1.33047 | 1.55E-26 | 1.21E-25 |
| *ENG* | 109.0457 | 38.34384 | -1.50787 | 6.59E-27 | 5.57E-26 |
| *MYEOV* | 0.072356 | 5.574777 | 6.267664 | 7.95E-25 | 4.92E-24 |
| *ICA1* | 4.357359 | 10.72119 | 1.298939 | 1.91E-24 | 1.13E-23 |
| *ENC1* | 6.889974 | 17.84031 | 1.37257 | 3.74E-18 | 1.19E-17 |
| *PTRH2* | 2.481353 | 5.418583 | 1.126789 | 5.84E-28 | 5.88E-27 |
| *MYCT1* | 12.10109 | 2.761106 | -2.13182 | 3.72E-31 | 1.32E-29 |
| *IBSP* | 0.043766 | 1.535763 | 5.132995 | 1.53E-18 | 5.04E-18 |
| *MYCN* | 0.15131 | 2.513851 | 4.054321 | 6.02E-09 | 1.02E-08 |
| *HYLS1* | 0.897705 | 2.480094 | 1.466081 | 5.40E-28 | 5.53E-27 |
| *EMP1* | 58.71203 | 18.82106 | -1.64131 | 4.46E-17 | 1.30E-16 |
| *CD1A* | 1.478375 | 5.071861 | 1.778503 | 0.002698 | 0.003273 |
| *CD19* | 0.777729 | 1.837927 | 1.24074 | 4.82E-13 | 1.07E-12 |
| *HYAL2* | 37.0282 | 16.0692 | -1.20433 | 4.67E-24 | 2.66E-23 |
| *EME2* | 0.968571 | 2.144513 | 1.14672 | 1.57E-09 | 2.77E-09 |
| *PTPRB* | 16.56042 | 2.991529 | -2.46878 | 4.45E-30 | 8.52E-29 |
| *EME1* | 0.191969 | 1.485486 | 2.951993 | 6.80E-30 | 1.16E-28 |
| *MYBL2* | 1.341937 | 20.88981 | 3.96041 | 4.46E-29 | 5.90E-28 |
| *EMCN* | 15.75972 | 2.537414 | -2.63481 | 4.09E-31 | 1.40E-29 |
| *MYBL1* | 0.480425 | 1.236264 | 1.363605 | 5.46E-11 | 1.06E-10 |
| *TACC3* | 2.41833 | 8.579051 | 1.826807 | 7.35E-26 | 5.18E-25 |
| *HTR2B* | 0.451526 | 1.906328 | 2.077915 | 0.014311 | 0.016488 |
| *TAC4* | 0.241976 | 1.971355 | 3.02625 | 4.30E-05 | 5.85E-05 |
| *MXD3* | 0.586303 | 1.582224 | 1.432235 | 4.25E-18 | 1.34E-17 |
| *CD101* | 6.624504 | 1.096525 | -2.59487 | 1.16E-29 | 1.81E-28 |
| *TAC3* | 0.020913 | 0.847049 | 5.339944 | 0.009735 | 0.011369 |
| *HSPD1* | 31.16234 | 84.66243 | 1.441918 | 1.08E-30 | 2.92E-29 |
| *MX2* | 1.679919 | 5.15473 | 1.617505 | 2.16E-19 | 7.65E-19 |
| *PTGIS* | 12.30017 | 5.539555 | -1.15084 | 4.21E-18 | 1.33E-17 |
| *HSPB7* | 3.180773 | 1.258246 | -1.33796 | 2.54E-21 | 1.07E-20 |
| *CCT6A* | 26.68779 | 64.61561 | 1.275703 | 1.80E-29 | 2.61E-28 |
| *PTGIR* | 3.480651 | 1.441028 | -1.27226 | 3.93E-22 | 1.82E-21 |
| *MUC20* | 2.445182 | 13.51792 | 2.466859 | 1.91E-16 | 5.31E-16 |
| *HSPB6* | 22.71359 | 4.278094 | -2.40852 | 7.46E-30 | 1.25E-28 |
| *CCT5* | 16.65184 | 44.51873 | 1.418731 | 8.65E-27 | 7.17E-26 |
| *SYT2* | 0.081027 | 0.659572 | 3.025057 | 2.58E-12 | 5.50E-12 |
| *PTGFRN* | 6.288227 | 15.85501 | 1.334214 | 4.21E-21 | 1.74E-20 |
| *MTX1* | 3.41628 | 6.911943 | 1.016665 | 2.19E-26 | 1.67E-25 |
| *CCT3* | 31.34997 | 88.4318 | 1.496101 | 6.16E-31 | 1.89E-29 |
| *PTGER4* | 10.28822 | 3.883394 | -1.4056 | 1.30E-26 | 1.04E-25 |
| *HSPA6* | 2.694124 | 7.366297 | 1.451123 | 1.33E-05 | 1.87E-05 |
| *CCT2* | 19.89766 | 42.7624 | 1.103744 | 4.60E-17 | 1.34E-16 |
| *PTGDS* | 87.65013 | 28.54209 | -1.61867 | 7.16E-23 | 3.58E-22 |
| *SYNGR3* | 0.1804 | 1.347253 | 2.900748 | 3.29E-24 | 1.91E-23 |
| *MTHFD2* | 6.031852 | 19.05726 | 1.659668 | 3.45E-22 | 1.60E-21 |
| *HSF4* | 1.148065 | 4.452234 | 1.955325 | 4.06E-13 | 9.10E-13 |
| *SYNE4* | 2.72192 | 5.921777 | 1.121405 | 4.36E-12 | 9.12E-12 |
| *HSF2BP* | 0.101526 | 0.552636 | 2.444479 | 9.72E-27 | 7.94E-26 |
| *MTFR2* | 0.54491 | 1.762871 | 1.693837 | 5.51E-16 | 1.49E-15 |
| *PTCRA* | 2.060007 | 0.412799 | -2.31914 | 6.55E-29 | 8.22E-28 |
| *SYCP2* | 0.310645 | 1.285395 | 2.048872 | 0.001251 | 0.001545 |
| *SYCE3* | 0.36513 | 1.408781 | 1.947966 | 5.40E-14 | 1.28E-13 |
| *MTBP* | 0.227906 | 0.858425 | 1.913253 | 3.55E-26 | 2.61E-25 |
| *CCNO* | 1.307427 | 6.338333 | 2.277373 | 1.72E-16 | 4.78E-16 |
| *SYCE2* | 0.229311 | 0.594721 | 1.374911 | 4.21E-18 | 1.33E-17 |
| *MSRB1* | 13.49801 | 29.89983 | 1.147391 | 2.56E-14 | 6.20E-14 |
| *CCNI2* | 0.158675 | 0.581726 | 1.874265 | 1.13E-12 | 2.47E-12 |
| *SYCE1L* | 0.672884 | 1.607142 | 1.256068 | 3.46E-08 | 5.61E-08 |
| *MSR1* | 37.7082 | 8.555869 | -2.13989 | 1.99E-28 | 2.22E-27 |
| *CCNF* | 0.813442 | 3.406404 | 2.066137 | 2.14E-29 | 3.03E-28 |
| *PSRC1* | 1.148028 | 2.967473 | 1.370077 | 4.38E-16 | 1.19E-15 |
| *CCNE2* | 0.300655 | 1.433913 | 2.253778 | 9.91E-27 | 8.05E-26 |
| *MSLNL* | 0.202976 | 1.594445 | 2.973677 | 0.041839 | 0.046652 |
| *CCNE1* | 0.364848 | 4.137125 | 3.503261 | 2.18E-30 | 4.69E-29 |
| *SVEP1* | 6.553906 | 1.719636 | -1.93025 | 1.99E-25 | 1.33E-24 |
| *PSPH* | 2.925579 | 7.655782 | 1.387828 | 1.54E-17 | 4.63E-17 |
| *EGR3* | 6.006515 | 1.918688 | -1.64641 | 2.75E-10 | 5.08E-10 |
| *CCND2* | 13.41507 | 6.687367 | -1.00434 | 2.39E-20 | 9.14E-20 |
| *EGR2* | 13.07469 | 4.109201 | -1.66985 | 7.25E-18 | 2.23E-17 |
| *PSMG3* | 7.172846 | 22.74497 | 1.66493 | 2.76E-28 | 2.98E-27 |
| *MSH5* | 0.213288 | 0.757278 | 1.828019 | 5.16E-16 | 1.39E-15 |
| *EGLN3* | 1.123861 | 9.935402 | 3.144115 | 2.73E-19 | 9.59E-19 |
| *CCNB2* | 0.853986 | 7.891069 | 3.207937 | 1.32E-30 | 3.31E-29 |
| *HPGDS* | 5.203569 | 2.215886 | -1.23162 | 1.95E-18 | 6.34E-18 |
| *CCNB1* | 1.996667 | 17.65845 | 3.144693 | 6.76E-32 | 5.62E-30 |
| *SULT1C4* | 3.669968 | 0.800563 | -2.19668 | 2.74E-28 | 2.96E-27 |
| *CCNA2* | 0.980256 | 8.193149 | 3.063188 | 8.18E-30 | 1.35E-28 |
| *SULT1C2* | 0.774142 | 2.487732 | 1.684162 | 0.000794 | 0.000996 |
| *MS4A7* | 32.91064 | 8.418406 | -1.96694 | 1.41E-29 | 2.11E-28 |
| *HPDL* | 0.111884 | 1.412269 | 3.657942 | 1.39E-24 | 8.39E-24 |
| *CCNA1* | 1.207094 | 0.587545 | -1.03877 | 1.01E-07 | 1.61E-07 |
| *SULF1* | 3.2877 | 20.93015 | 2.670432 | 7.93E-22 | 3.53E-21 |
| *EFNA5* | 2.331707 | 6.985329 | 1.582942 | 1.36E-16 | 3.82E-16 |
| *CCN4* | 0.750896 | 3.930672 | 2.388091 | 2.94E-22 | 1.38E-21 |
| *MS4A4A* | 20.18084 | 9.798363 | -1.04237 | 7.11E-18 | 2.19E-17 |
| *EFNA4* | 3.416884 | 17.06855 | 2.320588 | 8.00E-31 | 2.33E-29 |
| *CCN2* | 233.0455 | 85.76436 | -1.44216 | 2.50E-18 | 8.05E-18 |
| *STXBP6* | 6.596201 | 0.674383 | -3.29 | 1.57E-31 | 8.42E-30 |
| *HOXC13* | 0.006641 | 0.998089 | 7.231553 | 1.08E-12 | 2.36E-12 |
| *PSMB4* | 59.4835 | 119.6211 | 1.007911 | 6.17E-25 | 3.87E-24 |
| *MS4A2* | 3.8792 | 1.146233 | -1.75886 | 2.72E-22 | 1.28E-21 |
| *CCL7* | 0.348485 | 1.395245 | 2.001352 | 1.34E-09 | 2.37E-09 |
| *STX1A* | 0.576335 | 5.1932 | 3.171643 | 2.57E-31 | 1.04E-29 |
| *HOXB9* | 0.034223 | 5.640993 | 7.364853 | 6.88E-12 | 1.42E-11 |
| *EFEMP1* | 83.33421 | 30.30884 | -1.45917 | 6.52E-27 | 5.55E-26 |
| *EFCAB13* | 0.277315 | 0.71176 | 1.359863 | 7.19E-10 | 1.29E-09 |
| *HOXB13* | 0.009114 | 0.865584 | 6.569382 | 2.48E-08 | 4.06E-08 |
| *EDN1* | 48.42873 | 13.23203 | -1.87183 | 2.65E-21 | 1.11E-20 |
| *PSAT1* | 1.117012 | 14.35338 | 3.683673 | 6.69E-29 | 8.35E-28 |
| *HOXA4* | 1.837302 | 0.700514 | -1.3911 | 2.61E-23 | 1.38E-22 |
| *EDARADD* | 0.350888 | 1.126497 | 1.682761 | 0.027233 | 0.030766 |
| *CCL26* | 0.34467 | 0.887998 | 1.365342 | 0.000216 | 0.000281 |
| *STRA6* | 0.089037 | 2.291752 | 4.685902 | 5.78E-25 | 3.64E-24 |
| *CCL23* | 8.161916 | 1.564325 | -2.38337 | 2.91E-28 | 3.12E-27 |
| *PRX* | 22.04358 | 2.238714 | -3.29962 | 7.60E-31 | 2.28E-29 |
| *CCL22* | 2.71189 | 5.544523 | 1.031764 | 0.002125 | 0.002599 |
| *HOXA1* | 0.189521 | 0.868628 | 2.196381 | 0.00056 | 0.000712 |
| *ECT2* | 2.067709 | 10.52534 | 2.347762 | 5.47E-30 | 9.76E-29 |
| *MRPL24* | 17.27505 | 39.65103 | 1.198668 | 2.70E-25 | 1.77E-24 |
| *HORMAD1* | 0.121469 | 2.762715 | 4.507421 | 1.11E-05 | 1.56E-05 |
| *ECSCR* | 13.66605 | 2.812321 | -2.28076 | 2.97E-32 | 4.01E-30 |
| *CCL20* | 10.88857 | 29.14343 | 1.420356 | 6.00E-05 | 8.11E-05 |
| *ECE2* | 0.45081 | 2.133916 | 2.242911 | 2.88E-29 | 3.93E-28 |
| *CCL2* | 62.46581 | 24.09782 | -1.37416 | 1.67E-06 | 2.46E-06 |
| *STMN1* | 11.67343 | 26.46883 | 1.181066 | 8.28E-10 | 1.48E-09 |
| *CCL19* | 14.49633 | 32.44598 | 1.162352 | 2.31E-06 | 3.38E-06 |
| *STK32A* | 0.694086 | 3.355045 | 2.273146 | 1.23E-17 | 3.74E-17 |
| *PRRX2* | 0.930312 | 4.558731 | 2.292846 | 5.70E-09 | 9.68E-09 |
| *MRGBP* | 4.017238 | 9.67613 | 1.268226 | 1.28E-29 | 1.98E-28 |
| *CCL18* | 167.8241 | 82.63528 | -1.02212 | 7.38E-06 | 1.05E-05 |
| *STK31* | 0.228665 | 1.069172 | 2.225186 | 1.15E-17 | 3.51E-17 |
| *PRR7* | 0.607576 | 1.794636 | 1.562555 | 1.71E-15 | 4.41E-15 |
| *MRC1* | 84.70508 | 19.05436 | -2.15233 | 5.29E-28 | 5.44E-27 |
| *CCL14* | 2.145932 | 0.418934 | -2.35681 | 3.62E-23 | 1.87E-22 |
| *STIL* | 0.299118 | 2.045426 | 2.773615 | 1.32E-31 | 7.84E-30 |
| *E2F7* | 0.105446 | 0.804401 | 2.931412 | 1.79E-23 | 9.66E-23 |
| *STEAP1* | 2.096081 | 16.30018 | 2.959121 | 2.21E-21 | 9.37E-21 |
| *PRR19* | 0.1747 | 0.860591 | 2.30045 | 3.77E-27 | 3.29E-26 |
| *E2F5* | 1.157881 | 3.071487 | 1.40745 | 1.94E-23 | 1.03E-22 |
| *MPP6* | 0.697882 | 1.808514 | 1.373749 | 7.78E-17 | 2.22E-16 |
| *HMMR* | 0.458661 | 4.277791 | 3.221365 | 4.45E-30 | 8.52E-29 |
| *E2F3* | 2.157324 | 6.327146 | 1.552312 | 1.14E-28 | 1.36E-27 |
| *PRR11* | 0.876787 | 4.801469 | 2.453178 | 3.55E-23 | 1.84E-22 |
| *HMGB3* | 6.23546 | 78.42326 | 3.652714 | 1.11E-30 | 2.95E-29 |
| *E2F1* | 3.161181 | 9.920722 | 1.649981 | 9.27E-17 | 2.63E-16 |
| *HMGB2* | 16.47547 | 33.5615 | 1.026488 | 4.97E-14 | 1.19E-13 |
| *HMGA1* | 23.2964 | 125.8554 | 2.433588 | 8.03E-28 | 7.94E-27 |
| *CCDC34* | 1.244229 | 3.690533 | 1.568577 | 1.63E-21 | 7.02E-21 |
| *CCDC28B* | 1.074289 | 2.158365 | 1.006557 | 4.51E-13 | 1.01E-12 |
| *STAG3* | 0.35466 | 0.936637 | 1.401052 | 3.70E-05 | 5.07E-05 |
| *MOCOS* | 1.512699 | 3.233486 | 1.095966 | 4.92E-11 | 9.64E-11 |
| *STAC* | 6.67208 | 1.765308 | -1.91822 | 2.74E-26 | 2.05E-25 |
| *CCDC18* | 0.488842 | 0.986644 | 1.01316 | 6.15E-09 | 1.04E-08 |
| *MNDA* | 23.73555 | 9.641592 | -1.29971 | 2.30E-22 | 1.10E-21 |
| *DUSP23* | 31.1214 | 68.35065 | 1.135048 | 2.43E-13 | 5.52E-13 |
| *PRKCQ* | 4.196596 | 1.59792 | -1.39302 | 1.22E-24 | 7.46E-24 |
| *MND1* | 0.283239 | 2.431105 | 3.10152 | 2.08E-31 | 9.93E-30 |
| *CCDC167* | 9.933829 | 28.57483 | 1.524323 | 1.22E-24 | 7.46E-24 |
| *MMS22L* | 0.309213 | 0.648123 | 1.067668 | 3.26E-14 | 7.85E-14 |
| *CCDC154* | 0.174691 | 1.096018 | 2.649391 | 9.94E-11 | 1.91E-10 |
| *MMRN2* | 17.98892 | 4.400529 | -2.03136 | 4.74E-31 | 1.57E-29 |
| *CCDC151* | 1.926369 | 0.79672 | -1.27374 | 4.62E-05 | 6.27E-05 |
| *MMRN1* | 8.154556 | 1.742238 | -2.22666 | 1.87E-24 | 1.11E-23 |
| *MMP9* | 8.633327 | 36.96419 | 2.09814 | 7.56E-15 | 1.88E-14 |
| *DUS4L* | 0.819335 | 1.993076 | 1.282471 | 3.21E-28 | 3.42E-27 |
| *ST6GALNAC2* | 5.855441 | 2.181009 | -1.42478 | 3.65E-23 | 1.88E-22 |
| *PRIM1* | 1.981292 | 4.173344 | 1.074762 | 8.23E-13 | 1.81E-12 |
| *DUS1L* | 7.68331 | 19.22121 | 1.322899 | 2.73E-27 | 2.47E-26 |
| *DTYMK* | 5.28131 | 13.92916 | 1.39914 | 4.49E-25 | 2.86E-24 |
| *PRF1* | 15.22979 | 7.015996 | -1.11818 | 8.06E-15 | 2.01E-14 |
| *HK3* | 15.55438 | 4.999439 | -1.63748 | 8.16E-24 | 4.56E-23 |
| *CBX8* | 1.168083 | 3.267471 | 1.484032 | 2.43E-28 | 2.63E-27 |
| *PRELP* | 39.38951 | 14.19507 | -1.47242 | 2.91E-21 | 1.22E-20 |
| *MMP15* | 10.71559 | 23.74834 | 1.148116 | 7.23E-12 | 1.49E-11 |
| *HJURP* | 0.280555 | 4.596154 | 4.03407 | 1.19E-31 | 7.51E-30 |
| *PRELID3A* | 0.491229 | 1.072638 | 1.126695 | 4.43E-15 | 1.12E-14 |
| *MMP12* | 1.25021 | 21.25016 | 4.087232 | 5.09E-21 | 2.08E-20 |
| *DTL* | 0.898736 | 3.663616 | 2.027299 | 2.58E-22 | 1.22E-21 |
| *CBX3* | 21.0803 | 48.71002 | 1.208323 | 7.77E-30 | 1.29E-28 |
| *CBX2* | 0.594703 | 3.47408 | 2.54639 | 2.48E-19 | 8.76E-19 |
| *MME* | 10.39281 | 2.117597 | -2.29509 | 3.03E-29 | 4.12E-28 |
| *DSN1* | 4.066731 | 9.337309 | 1.199137 | 2.76E-23 | 1.45E-22 |
| *DSCC1* | 0.857122 | 3.260835 | 1.927668 | 5.44E-22 | 2.50E-21 |
| *PRC1* | 1.011182 | 6.573056 | 2.700522 | 5.43E-31 | 1.70E-29 |
| *PRAP1* | 0.09801 | 1.783385 | 4.185545 | 0.002775 | 0.003364 |
| *SRXN1* | 0.610746 | 1.743401 | 1.513261 | 3.52E-05 | 4.82E-05 |
| *PRAME* | 0.03597 | 7.449689 | 7.694233 | 1.17E-19 | 4.28E-19 |
| *MKI67* | 0.674343 | 6.723742 | 3.31771 | 9.63E-29 | 1.17E-27 |
| *CAVIN2* | 119.2031 | 13.02845 | -3.19369 | 6.87E-33 | 2.86E-30 |
| *MITF* | 4.425299 | 2.180711 | -1.02098 | 1.30E-23 | 7.09E-23 |
| *CAV1* | 346.1085 | 35.14271 | -3.29993 | 9.40E-33 | 2.86E-30 |
| *MIS18A* | 3.04584 | 8.152594 | 1.420419 | 8.35E-30 | 1.37E-28 |
| *DPY19L1* | 8.197175 | 20.2105 | 1.301906 | 9.06E-17 | 2.58E-16 |
| *CAT* | 100.1085 | 29.37069 | -1.76912 | 1.58E-32 | 3.06E-30 |
| *SRSF12* | 0.113524 | 0.559966 | 2.302342 | 6.39E-16 | 1.71E-15 |
| *DPT* | 35.46102 | 13.74013 | -1.36784 | 9.37E-16 | 2.46E-15 |
| *SRRM3* | 0.127494 | 0.718747 | 2.495058 | 7.66E-22 | 3.42E-21 |
| *PPP1R35* | 6.330869 | 16.62339 | 1.392739 | 1.71E-23 | 9.25E-23 |
| *MIF* | 17.81028 | 44.55402 | 1.322846 | 4.76E-19 | 1.64E-18 |
| *DPP4* | 10.72089 | 29.92875 | 1.481107 | 0.000545 | 0.000694 |

**Table S4.** 326 prognostic-related genes for establishing the signature.

| ID | HR | HR.95L | HR.95H | *p*-value |
| --- | --- | --- | --- | --- |
| *SRPK1* | 1.51067 | 1.1043 | 2.06659 | 0.009866 |
| *PPP1R16B* | 0.74406 | 0.60387 | 0.9168 | 0.005511 |
| *DPEP2* | 0.6538 | 0.5034 | 0.84914 | 0.001442 |
| *CAPN13* | 0.89258 | 0.79983 | 0.99607 | 0.042328 |
| *MFAP4* | 0.85029 | 0.75324 | 0.95985 | 0.008726 |
| *SPOCK2* | 0.79997 | 0.67645 | 0.94603 | 0.009098 |
| *SPN* | 0.72313 | 0.57388 | 0.9112 | 0.005989 |
| *PPAT* | 1.33616 | 1.04357 | 1.7108 | 0.021554 |
| *DNAJC9* | 1.60905 | 1.15153 | 2.24834 | 0.005327 |
| *MELK* | 1.19028 | 1.02852 | 1.37748 | 0.019426 |
| *SPC25* | 1.45288 | 1.15606 | 1.82592 | 0.001357 |
| *POU2AF1* | 0.82761 | 0.70159 | 0.97626 | 0.024769 |
| *SPC24* | 1.19641 | 1.0068 | 1.42173 | 0.04165 |
| *CA9* | 1.11403 | 1.02795 | 1.20732 | 0.008489 |
| *CA4* | 0.78101 | 0.63903 | 0.95454 | 0.015758 |
| *SOX9* | 1.159 | 1.03432 | 1.29871 | 0.01105 |
| *POLE2* | 1.26322 | 1.02842 | 1.55163 | 0.025946 |
| *HELLS* | 1.28573 | 1.00665 | 1.64219 | 0.044113 |
| *MCM8* | 1.50047 | 1.11105 | 2.02636 | 0.008124 |
| *MCM7* | 1.28331 | 1.02937 | 1.59991 | 0.026608 |
| *MCM6* | 1.32166 | 1.04954 | 1.66434 | 0.017739 |
| *MCM4* | 1.31547 | 1.10589 | 1.56476 | 0.001957 |
| *DLC1* | 0.8179 | 0.68965 | 0.97 | 0.020889 |
| *MCM2* | 1.24419 | 1.04852 | 1.47638 | 0.012325 |
| *HAVCR1* | 1.33894 | 1.11815 | 1.60334 | 0.001501 |
| *C1QTNF6* | 1.41305 | 1.12346 | 1.7773 | 0.003129 |
| *ZIC2* | 1.20435 | 1.01121 | 1.43436 | 0.037068 |
| *SNX22* | 0.67401 | 0.45444 | 0.99969 | 0.049819 |
| *HAL* | 1.19567 | 1.02113 | 1.40006 | 0.026445 |
| *DHFR* | 1.81188 | 1.29584 | 2.53343 | 0.00051 |
| *SNRPE* | 1.52791 | 1.09501 | 2.13195 | 0.012632 |
| *SNRPA1* | 1.42151 | 1.01533 | 1.99017 | 0.040504 |
| *MARCKSL1* | 1.26348 | 1.03499 | 1.54241 | 0.021567 |
| *DEPDC1B* | 1.42914 | 1.17028 | 1.74526 | 0.000461 |
| *DEPDC1* | 1.40347 | 1.14108 | 1.7262 | 0.001329 |
| *C19orf48* | 1.42716 | 1.15337 | 1.76595 | 0.001065 |
| *PLK4* | 1.2945 | 1.03294 | 1.62228 | 0.024999 |
| *PLK1* | 1.38182 | 1.15326 | 1.65568 | 0.000455 |
| *GTSE1* | 1.29591 | 1.07244 | 1.56593 | 0.00727 |
| *MAOB* | 0.81011 | 0.6809 | 0.96385 | 0.017536 |
| *PLEKHH2* | 0.72544 | 0.56931 | 0.9244 | 0.00944 |
| *BZW2* | 1.39255 | 1.03098 | 1.88092 | 0.030862 |
| *MAL* | 0.79996 | 0.66744 | 0.9588 | 0.015725 |
| *BUB1B* | 1.33004 | 1.09535 | 1.61501 | 0.003984 |
| *BUB1* | 1.2444 | 1.04995 | 1.47486 | 0.011661 |
| *DDX11* | 1.34648 | 1.03693 | 1.74844 | 0.025614 |
| *PLEK2* | 1.47573 | 1.25041 | 1.74165 | 4.15E-06 |
| *BST1* | 0.72205 | 0.55102 | 0.94616 | 0.018216 |
| *DCN* | 0.85667 | 0.74434 | 0.98596 | 0.030993 |
| *BRIP1* | 1.47239 | 1.06856 | 2.02885 | 0.018011 |
| *GPX2* | 1.08171 | 1.02314 | 1.14362 | 0.005684 |
| *MAD2L1* | 1.32657 | 1.09522 | 1.60678 | 0.00385 |
| *BRCA1* | 1.33969 | 1.031 | 1.74082 | 0.02864 |
| *WNT7A* | 1.24489 | 1.0117 | 1.53184 | 0.038465 |
| *SLC7A11* | 1.2307 | 1.07696 | 1.40638 | 0.002297 |
| *PKMYT1* | 1.37639 | 1.10471 | 1.71488 | 0.004405 |
| *DAPK2* | 0.62941 | 0.47311 | 0.83735 | 0.001479 |
| *BOP1* | 1.24696 | 1.00306 | 1.55017 | 0.046867 |
| *LY86* | 0.83741 | 0.71105 | 0.98623 | 0.033492 |
| *GPR18* | 0.68233 | 0.50242 | 0.92667 | 0.01438 |
| *WDR76* | 1.47315 | 1.11119 | 1.95301 | 0.007085 |
| *GPR174* | 0.58395 | 0.41525 | 0.82119 | 0.001985 |
| *PIP5KL1* | 0.79989 | 0.64011 | 0.99957 | 0.04956 |
| *BLM* | 1.35509 | 1.04411 | 1.75871 | 0.022346 |
| *WDHD1* | 1.44753 | 1.13071 | 1.85312 | 0.003339 |
| *SLC31A2* | 0.70631 | 0.52808 | 0.94469 | 0.019108 |
| *GPR146* | 0.56164 | 0.32191 | 0.97989 | 0.042204 |
| *PIM2* | 0.81115 | 0.68447 | 0.96129 | 0.015708 |
| *BIRC5* | 1.24291 | 1.0679 | 1.44659 | 0.004978 |
| *LST1* | 0.79577 | 0.66347 | 0.95447 | 0.013805 |
| *SLC2A1* | 1.30124 | 1.14163 | 1.48315 | 8.01E-05 |
| *CYP27A1* | 0.76288 | 0.64332 | 0.90467 | 0.001859 |
| *BIK* | 1.27352 | 1.06429 | 1.52388 | 0.008282 |
| *GPI* | 1.53575 | 1.19906 | 1.967 | 0.00068 |
| *LRRN3* | 0.62943 | 0.43614 | 0.90837 | 0.013383 |
| *BCL2L10* | 1.62053 | 1.29629 | 2.02588 | 2.25E-05 |
| *BARX2* | 1.1741 | 1.00021 | 1.37823 | 0.0497 |
| *BARX1* | 1.09589 | 1.00709 | 1.19251 | 0.033676 |
| *LRFN4* | 1.30386 | 1.07842 | 1.57644 | 0.006154 |
| *SLC16A3* | 1.43581 | 1.17931 | 1.74811 | 0.000315 |
| *PFN2* | 1.17744 | 1.01357 | 1.36781 | 0.032658 |
| *PFKP* | 1.35255 | 1.15506 | 1.5838 | 0.000177 |
| *LPGAT1* | 1.41089 | 1.05062 | 1.89469 | 0.022123 |
| *BACE2* | 1.2049 | 1.03544 | 1.40208 | 0.015938 |
| *CTSG* | 0.79529 | 0.65012 | 0.97288 | 0.025926 |
| *SLC15A2* | 0.76754 | 0.63713 | 0.92465 | 0.005361 |
| *PECAM1* | 0.72459 | 0.58311 | 0.90039 | 0.003654 |
| *LMNB2* | 1.3112 | 1.05507 | 1.62952 | 0.01455 |
| *B3GALNT1* | 1.33764 | 1.09065 | 1.64057 | 0.005221 |
| *LMNB1* | 1.35702 | 1.11395 | 1.65314 | 0.002433 |
| *CTLA4* | 0.67442 | 0.52963 | 0.85879 | 0.001401 |
| *AURKB* | 1.20943 | 1.04815 | 1.39554 | 0.009217 |
| *AURKA* | 1.25775 | 1.07149 | 1.4764 | 0.005043 |
| *GIMAP5* | 0.5851 | 0.41448 | 0.82597 | 0.002312 |
| *GGH* | 1.17878 | 1.03807 | 1.33856 | 0.011208 |
| *SIRPG* | 0.75157 | 0.58079 | 0.97256 | 0.029892 |
| *GFRA1* | 0.55836 | 0.3425 | 0.91025 | 0.019434 |
| *LILRA6* | 0.67104 | 0.46576 | 0.9668 | 0.03226 |
| *UHRF1* | 1.344 | 1.07926 | 1.67368 | 0.008256 |
| *SIGLEC6* | 0.41531 | 0.23725 | 0.72701 | 0.002098 |
| *PCSK9* | 1.16657 | 1.01143 | 1.34551 | 0.034337 |
| *CRY2* | 0.77522 | 0.61286 | 0.9806 | 0.033725 |
| *GDF15* | 0.88548 | 0.79447 | 0.98691 | 0.02794 |
| *UBE2T* | 1.2555 | 1.06399 | 1.48148 | 0.007051 |
| *SHOX2* | 1.328 | 1.04172 | 1.69294 | 0.022024 |
| *UBE2S* | 1.36846 | 1.12083 | 1.67079 | 0.00207 |
| *PCNA* | 1.33476 | 1.04769 | 1.70048 | 0.019436 |
| *ATIC* | 1.64691 | 1.14619 | 2.36639 | 0.006981 |
| *SHCBP1* | 1.36892 | 1.07495 | 1.74328 | 0.010899 |
| *UBE2C* | 1.16241 | 1.02744 | 1.31509 | 0.016851 |
| *LDHA* | 1.62301 | 1.25151 | 2.10479 | 0.000261 |
| *LDB2* | 0.71028 | 0.55644 | 0.90664 | 0.006016 |
| *ATAD2* | 1.21465 | 1.01966 | 1.44693 | 0.029403 |
| *ASPM* | 1.33688 | 1.12872 | 1.58343 | 0.000773 |
| *TYMS* | 1.36907 | 1.15424 | 1.62389 | 0.00031 |
| *GAPDH* | 1.59651 | 1.27748 | 1.99523 | 3.91E-05 |
| *ASPH* | 1.27568 | 1.08067 | 1.50588 | 0.004021 |
| *ASF1B* | 1.2526 | 1.0457 | 1.50043 | 0.01448 |
| *SFXN1* | 1.43499 | 1.11044 | 1.85439 | 0.005767 |
| *CPNE5* | 0.77719 | 0.6111 | 0.98842 | 0.039886 |
| *PBK* | 1.31967 | 1.13496 | 1.53444 | 0.000312 |
| *TTK* | 1.23964 | 1.04271 | 1.47376 | 0.01494 |
| *ARRB1* | 0.66336 | 0.52703 | 0.83496 | 0.000471 |
| *CPA3* | 0.87636 | 0.78505 | 0.97829 | 0.018725 |
| *PAQR5* | 1.2582 | 1.02413 | 1.54577 | 0.028746 |
| *PAQR4* | 1.31194 | 1.06262 | 1.61976 | 0.011578 |
| *ARHGEF15* | 0.72287 | 0.52706 | 0.99142 | 0.044069 |
| *TRPV2* | 0.78583 | 0.63766 | 0.96842 | 0.023762 |
| *KPNA2* | 1.33058 | 1.08651 | 1.62947 | 0.005737 |
| *COLEC12* | 0.82523 | 0.69228 | 0.9837 | 0.03209 |
| *SELP* | 0.73148 | 0.57905 | 0.92404 | 0.008726 |
| *TROAP* | 1.16692 | 1.00465 | 1.35539 | 0.043311 |
| *SEC61G* | 1.55797 | 1.24311 | 1.95257 | 0.000118 |
| *FURIN* | 1.20835 | 1.05275 | 1.38694 | 0.007127 |
| *ARHGAP11A* | 1.53431 | 1.22314 | 1.92463 | 0.000214 |
| *PABPC1* | 1.36417 | 1.04009 | 1.78924 | 0.024835 |
| *TRIP13* | 1.22402 | 1.05109 | 1.42541 | 0.009292 |
| *P4HA1* | 1.44371 | 1.14728 | 1.81673 | 0.001738 |
| *P2RY14* | 0.51642 | 0.35567 | 0.74981 | 0.000514 |
| *TRIM59* | 1.41687 | 1.01976 | 1.96862 | 0.03784 |
| *FSCN1* | 1.23458 | 1.07695 | 1.41529 | 0.002498 |
| *P2RX1* | 0.59707 | 0.42613 | 0.83657 | 0.002728 |
| *TRIM16L* | 1.20825 | 1.02534 | 1.42378 | 0.023895 |
| *SCG2* | 1.13538 | 1.0145 | 1.27065 | 0.027059 |
| *FPR1* | 0.8606 | 0.74263 | 0.99731 | 0.045963 |
| *FOXP3* | 0.74185 | 0.56841 | 0.96821 | 0.02797 |
| *FOXM1* | 1.29202 | 1.10976 | 1.50422 | 0.000959 |
| *AP1S1* | 1.35633 | 1.04484 | 1.76068 | 0.022054 |
| *TPX2* | 1.26637 | 1.10587 | 1.45016 | 0.000637 |
| *KIFC1* | 1.23826 | 1.05585 | 1.45218 | 0.008581 |
| *CLSPN* | 1.36992 | 1.07709 | 1.74235 | 0.010311 |
| *KIF4A* | 1.30901 | 1.1065 | 1.54859 | 0.001689 |
| *ANLN* | 1.35148 | 1.15903 | 1.57589 | 0.000122 |
| *KIF2C* | 1.2572 | 1.07795 | 1.46626 | 0.003542 |
| *OIP5* | 1.36966 | 1.13963 | 1.64613 | 0.000799 |
| *TPI1* | 1.55261 | 1.17135 | 2.05797 | 0.002213 |
| *KIF23* | 1.33511 | 1.09706 | 1.62481 | 0.00392 |
| *KIF20A* | 1.39408 | 1.15818 | 1.67803 | 0.000444 |
| *CLIC6* | 0.86407 | 0.78671 | 0.94903 | 0.002264 |
| *KIF18A* | 1.58184 | 1.17169 | 2.13555 | 0.002747 |
| *RXRG* | 0.64177 | 0.41622 | 0.98956 | 0.044698 |
| *KIF15* | 1.24085 | 1.00405 | 1.53349 | 0.045785 |
| *FLI1* | 0.71323 | 0.53866 | 0.94437 | 0.018296 |
| *KIF14* | 1.44698 | 1.16971 | 1.78999 | 0.000664 |
| *TOP2A* | 1.17582 | 1.03291 | 1.33851 | 0.014297 |
| *FKBP4* | 1.4445 | 1.17479 | 1.77614 | 0.000487 |
| *KIF11* | 1.3357 | 1.11206 | 1.6043 | 0.001961 |
| *NUSAP1* | 1.32286 | 1.11022 | 1.57621 | 0.001751 |
| *CLEC1A* | 0.65116 | 0.45895 | 0.92387 | 0.016234 |
| *FGR* | 0.7846 | 0.64178 | 0.95921 | 0.017974 |
| *TNFSF13* | 0.75755 | 0.593 | 0.96776 | 0.02627 |
| *NUF2* | 1.24453 | 1.02962 | 1.5043 | 0.023712 |
| *RRM2* | 1.37719 | 1.17079 | 1.61998 | 0.000112 |
| *FGD5* | 0.77627 | 0.61028 | 0.98741 | 0.039093 |
| *CKS1B* | 1.34197 | 1.11396 | 1.61665 | 0.001963 |
| *TNFRSF17* | 0.82441 | 0.70903 | 0.95858 | 0.012078 |
| *CKAP4* | 1.46475 | 1.13583 | 1.88892 | 0.003266 |
| *FEN1* | 1.40584 | 1.11112 | 1.77874 | 0.004543 |
| *ALDOA* | 1.4409 | 1.11384 | 1.864 | 0.005424 |
| *TNFRSF11A* | 1.41536 | 1.02735 | 1.9499 | 0.033583 |
| *KCNJ15* | 0.83731 | 0.71767 | 0.9769 | 0.024007 |
| *FCRL5* | 0.74263 | 0.5713 | 0.96534 | 0.02618 |
| *AKR1B10* | 1.1123 | 1.04358 | 1.18556 | 0.001073 |
| *FCRL2* | 0.65306 | 0.46445 | 0.91827 | 0.014273 |
| *FCN1* | 0.75255 | 0.62925 | 0.90001 | 0.001847 |
| *CHML* | 1.21052 | 1.00165 | 1.46295 | 0.048039 |
| *KAZALD1* | 1.27634 | 1.02554 | 1.58847 | 0.028823 |
| *CHI3L1* | 0.8932 | 0.79877 | 0.9988 | 0.047597 |
| *CHEK1* | 1.40535 | 1.15977 | 1.70293 | 0.000516 |
| *CHD1L* | 1.46046 | 1.07278 | 1.98824 | 0.016114 |
| *CHAF1B* | 1.40263 | 1.09192 | 1.80176 | 0.00809 |
| *FBXO5* | 1.32524 | 1.00016 | 1.75597 | 0.049869 |
| *CFTR* | 0.76585 | 0.6412 | 0.91473 | 0.003248 |
| *AFF3* | 0.70575 | 0.50063 | 0.99491 | 0.046692 |
| *ADRB2* | 0.69858 | 0.55132 | 0.88518 | 0.002981 |
| *FBN2* | 1.28936 | 1.07722 | 1.54326 | 0.005587 |
| *ITGB4* | 1.25854 | 1.09415 | 1.44763 | 0.001283 |
| *FBLN5* | 0.77396 | 0.63728 | 0.93995 | 0.009749 |
| *CEP55* | 1.25916 | 1.07306 | 1.47753 | 0.00474 |
| *ITGAL* | 0.74242 | 0.62196 | 0.88621 | 0.000976 |
| *CENPO* | 1.45223 | 1.04111 | 2.0257 | 0.028006 |
| *TM6SF1* | 0.66446 | 0.5095 | 0.86655 | 0.002551 |
| *CENPM* | 1.3727 | 1.14256 | 1.6492 | 0.000716 |
| *RGS20* | 1.53854 | 1.22247 | 1.93632 | 0.000241 |
| *FANCD2* | 1.43533 | 1.02721 | 2.0056 | 0.034238 |
| *CENPH* | 1.69619 | 1.27941 | 2.24876 | 0.00024 |
| *CENPF* | 1.2774 | 1.09326 | 1.49256 | 0.002052 |
| *TLR8* | 0.73971 | 0.58835 | 0.93 | 0.009844 |
| *FANCA* | 1.47768 | 1.07036 | 2.04001 | 0.017633 |
| *CENPE* | 1.36228 | 1.08113 | 1.71653 | 0.008756 |
| *CENPA* | 1.24243 | 1.053 | 1.46594 | 0.010116 |
| *TLR10* | 0.68409 | 0.50652 | 0.92389 | 0.013277 |
| *IRF4* | 0.72299 | 0.57733 | 0.90541 | 0.00472 |
| *TK1* | 1.28532 | 1.08936 | 1.51652 | 0.002938 |
| *IQGAP3* | 1.26613 | 1.04485 | 1.53427 | 0.016055 |
| *RFC4* | 1.254 | 1.03992 | 1.51215 | 0.017795 |
| *CDT1* | 1.44925 | 1.19401 | 1.75906 | 0.000174 |
| *CDKN3* | 1.35569 | 1.15483 | 1.59148 | 0.0002 |
| *TIMELESS* | 1.29898 | 1.0511 | 1.60531 | 0.015468 |
| *ACVRL1* | 0.78746 | 0.62532 | 0.99166 | 0.042235 |
| *CDK5R1* | 1.53228 | 1.20778 | 1.94396 | 0.00044 |
| *ACTL6A* | 1.35639 | 1.05686 | 1.74081 | 0.016648 |
| *NFATC1* | 0.65111 | 0.44391 | 0.95501 | 0.028129 |
| *FAM111B* | 1.39022 | 1.12414 | 1.71928 | 0.002369 |
| *CDH24* | 1.32544 | 1.06757 | 1.64561 | 0.010703 |
| *CDH2* | 1.22329 | 1.05941 | 1.41253 | 0.006025 |
| *ACP5* | 0.83793 | 0.70394 | 0.99742 | 0.046698 |
| *IL7R* | 0.81872 | 0.70973 | 0.94444 | 0.006066 |
| *CDCA8* | 1.27404 | 1.07651 | 1.50783 | 0.004838 |
| *CDCA5* | 1.30158 | 1.10644 | 1.53113 | 0.001471 |
| *CDCA4* | 1.4276 | 1.10255 | 1.84848 | 0.006922 |
| *CDCA3* | 1.31018 | 1.08346 | 1.58436 | 0.005324 |
| *IL3RA* | 0.72937 | 0.55889 | 0.95186 | 0.020169 |
| *CDCA2* | 1.48625 | 1.18855 | 1.85852 | 0.000512 |
| *ACAD8* | 0.70095 | 0.5565 | 0.88289 | 0.002546 |
| *TFF2* | 1.08673 | 1.0124 | 1.16652 | 0.021402 |
| *NEK2* | 1.34523 | 1.13616 | 1.59277 | 0.000579 |
| *F8* | 0.6814 | 0.47379 | 0.97999 | 0.038547 |
| *CDC6* | 1.29832 | 1.0794 | 1.56165 | 0.00559 |
| *TFF1* | 1.07741 | 1.01985 | 1.13822 | 0.007777 |
| *NEIL3* | 1.33907 | 1.11055 | 1.61461 | 0.002226 |
| *CDC25C* | 1.67611 | 1.31313 | 2.13943 | 3.36E-05 |
| *ABI3BP* | 0.74251 | 0.60578 | 0.9101 | 0.004142 |
| *F12* | 1.36939 | 1.1217 | 1.67177 | 0.002014 |
| *CDC25A* | 1.44773 | 1.11844 | 1.87397 | 0.004953 |
| *CDC20* | 1.20876 | 1.05575 | 1.38395 | 0.006041 |
| *RASGRP2* | 0.55676 | 0.39223 | 0.79031 | 0.00105 |
| *TEK* | 0.76274 | 0.61556 | 0.94511 | 0.013285 |
| *CD79A* | 0.82653 | 0.73061 | 0.93503 | 0.002468 |
| *RANBP1* | 1.29723 | 1.01706 | 1.65458 | 0.03606 |
| *EXO1* | 1.42941 | 1.16234 | 1.75784 | 0.00071 |
| *NCF1* | 0.7901 | 0.6368 | 0.98032 | 0.032308 |
| *EVI2B* | 0.81182 | 0.67691 | 0.97362 | 0.024558 |
| *NCAPG2* | 1.40151 | 1.10744 | 1.77367 | 0.004966 |
| *RAD54L* | 1.25701 | 1.00888 | 1.56617 | 0.041471 |
| *ESCO2* | 1.69651 | 1.18984 | 2.41895 | 0.003497 |
| *RAD51AP1* | 1.18455 | 1.00182 | 1.40061 | 0.047558 |
| *CD52* | 0.83181 | 0.72173 | 0.95868 | 0.011002 |
| *RACGAP1* | 1.27899 | 1.04184 | 1.57011 | 0.018688 |
| *IGF2BP3* | 1.20328 | 1.06179 | 1.36362 | 0.00374 |
| *ERG* | 0.54333 | 0.37799 | 0.781 | 0.000984 |
| *IGF2BP1* | 1.40442 | 1.20022 | 1.64335 | 2.27E-05 |
| *RAB3B* | 1.25017 | 1.03842 | 1.50509 | 0.018365 |
| *CD37* | 0.81145 | 0.68869 | 0.95609 | 0.012541 |
| *NAALADL2* | 1.39176 | 1.05514 | 1.83576 | 0.019289 |
| *CD33* | 0.66927 | 0.51434 | 0.87087 | 0.002797 |
| *CD302* | 0.63095 | 0.49989 | 0.79636 | 0.000106 |
| *CD300LF* | 0.78472 | 0.65514 | 0.93993 | 0.008471 |
| *CD244* | 0.65546 | 0.4384 | 0.97997 | 0.039537 |
| *TAL1* | 0.57323 | 0.33473 | 0.98167 | 0.042626 |
| *MYEOV* | 1.11873 | 1.00308 | 1.24772 | 0.043893 |
| *CD19* | 0.79538 | 0.66845 | 0.94641 | 0.009856 |
| *MYBL2* | 1.14262 | 1.00852 | 1.29454 | 0.036329 |
| *TACC3* | 1.38854 | 1.12008 | 1.72133 | 0.002749 |
| *HSPD1* | 1.68521 | 1.26722 | 2.24106 | 0.000333 |
| *HSPB7* | 0.73224 | 0.54101 | 0.99106 | 0.043576 |
| *CCT6A* | 1.61349 | 1.28434 | 2.02699 | 3.96E-05 |
| *HSPB6* | 0.81464 | 0.67726 | 0.9799 | 0.029595 |
| *CCT5* | 1.32477 | 1.0462 | 1.67753 | 0.019548 |
| *PTGFRN* | 1.31563 | 1.03044 | 1.67976 | 0.027772 |
| *CCT3* | 1.46011 | 1.0855 | 1.964 | 0.012338 |
| *CCT2* | 1.18745 | 1.02924 | 1.36998 | 0.018523 |
| *PTGDS* | 0.82026 | 0.72505 | 0.92797 | 0.001646 |
| *MTHFD2* | 1.23381 | 1.00904 | 1.50864 | 0.040591 |
| *HSF2BP* | 1.56067 | 1.09491 | 2.22456 | 0.013842 |
| *PTCRA* | 0.49371 | 0.27853 | 0.87512 | 0.015661 |
| *MTBP* | 1.42951 | 1.0534 | 1.9399 | 0.021794 |
| *PSPH* | 1.28406 | 1.05669 | 1.56034 | 0.011918 |
| *CCND2* | 0.79945 | 0.65856 | 0.97048 | 0.023644 |
| *EGR2* | 0.8172 | 0.67791 | 0.9851 | 0.034224 |
| *EGLN3* | 1.16391 | 1.02923 | 1.31622 | 0.015559 |
| *CCNB2* | 1.2824 | 1.09638 | 1.49999 | 0.001866 |
| *CCNB1* | 1.40173 | 1.17894 | 1.66663 | 0.000131 |
| *CCNA2* | 1.32276 | 1.12931 | 1.54935 | 0.000526 |
| *MS4A7* | 0.82167 | 0.69548 | 0.97076 | 0.020953 |
| *EFNA5* | 1.25114 | 1.02835 | 1.5222 | 0.025134 |
| *MS4A2* | 0.72344 | 0.56889 | 0.91997 | 0.008283 |
| *CCL23* | 0.78351 | 0.62076 | 0.98892 | 0.039996 |
| *HOXA1* | 1.33116 | 1.04477 | 1.69605 | 0.02065 |
| *ECT2* | 1.42504 | 1.19858 | 1.69429 | 6.03E-05 |
| *STK32A* | 0.8313 | 0.6978 | 0.99035 | 0.038586 |
| *PRR7* | 1.2815 | 1.01157 | 1.62346 | 0.039849 |
| *MRC1* | 0.87047 | 0.76485 | 0.99068 | 0.035572 |
| *CCL14* | 0.67606 | 0.50803 | 0.89967 | 0.00725 |
| *STIL* | 1.28285 | 1.00825 | 1.63224 | 0.042684 |
| *E2F7* | 1.69087 | 1.35535 | 2.10944 | 3.25E-06 |
| *HMMR* | 1.4522 | 1.21485 | 1.73593 | 4.18E-05 |
| *PRR11* | 1.34523 | 1.11648 | 1.62084 | 0.001817 |
| *HMGB2* | 1.25504 | 1.02398 | 1.53824 | 0.028651 |
| *HMGA1* | 1.33105 | 1.11585 | 1.58776 | 0.001482 |
| *CCDC34* | 1.38855 | 1.10351 | 1.74722 | 0.005108 |
| *MNDA* | 0.82878 | 0.71464 | 0.96115 | 0.012986 |
| *MND1* | 1.34437 | 1.06261 | 1.70084 | 0.013663 |
| *MMRN1* | 0.80659 | 0.66245 | 0.98209 | 0.032367 |
| *PRIM1* | 1.32217 | 1.06753 | 1.63756 | 0.010509 |
| *DTYMK* | 1.49764 | 1.1473 | 1.95497 | 0.002972 |
| *HK3* | 0.83457 | 0.71156 | 0.97883 | 0.026221 |
| *PRELP* | 0.84603 | 0.71764 | 0.9974 | 0.046487 |
| *DTL* | 1.50294 | 1.19674 | 1.88749 | 0.000456 |
| *CBX2* | 1.23876 | 1.02087 | 1.50315 | 0.030067 |
| *PRC1* | 1.43774 | 1.20004 | 1.72251 | 8.22E-05 |
| *SRXN1* | 1.22173 | 1.03487 | 1.44233 | 0.018044 |
| *MKI67* | 1.32491 | 1.11159 | 1.57917 | 0.001683 |
| *CAT* | 0.76655 | 0.6119 | 0.96027 | 0.020745 |
| *MIF* | 1.34538 | 1.0936 | 1.65514 | 0.005012 |
| *DPP4* | 0.88274 | 0.79526 | 0.97983 | 0.019149 |

**Table S5.** The relationship between immune cells and RS.

| Method | Immune | Cor | *p*-value |
| --- | --- | --- | --- |
| CIBERSORT | B cell memory | -0.22 | 0.00 |
| CIBERSORT | Macrophage M0 | 0.17 | 0.00 |
| CIBERSORT | Mast cell activated | -0.16 | 0.00 |
| CIBERSORT | Mast cell resting | 0.18 | 0.00 |
| CIBERSORT | Monocyte | -0.14 | 0.00 |
| CIBERSORT | Myeloid dendritic cell activated | 0.10 | 0.02 |
| CIBERSORT | Myeloid dendritic cell resting | -0.09 | 0.05 |
| CIBERSORT | T cell CD4+ memory resting | -0.18 | 0.00 |
| CIBERSORT | T cell gamma delta | -0.11 | 0.01 |
| CIBERSORT-ABS | B cell memory | -0.28 | 0.00 |
| CIBERSORT-ABS | B cell plasma | -0.13 | 0.01 |
| CIBERSORT-ABS | Macrophage M1 | -0.18 | 0.00 |
| CIBERSORT-ABS | Macrophage M2 | -0.34 | 0.00 |
| CIBERSORT-ABS | Mast cell activated | -0.24 | 0.00 |
| CIBERSORT-ABS | Mast cell resting | 0.14 | 0.00 |
| CIBERSORT-ABS | Monocyte | -0.24 | 0.00 |
| CIBERSORT-ABS | Myeloid dendritic cell resting | -0.15 | 0.00 |
| CIBERSORT-ABS | NK cell activated | -0.17 | 0.00 |
| CIBERSORT-ABS | T cell CD4+ memory resting | -0.36 | 0.00 |
| CIBERSORT-ABS | T cell CD8+ | -0.28 | 0.00 |
| CIBERSORT-ABS | T cell follicular helper | -0.23 | 0.00 |
| CIBERSORT-ABS | T cell gamma delta | -0.12 | 0.01 |
| CIBERSORT-ABS | T cell regulatory (Tregs) | -0.17 | 0.00 |
| EPIC | B cell | -0.37 | 0.00 |
| EPIC | Endothelial cell | -0.25 | 0.00 |
| EPIC | Macrophage | -0.29 | 0.00 |
| EPIC | T cell CD4+ | -0.14 | 0.00 |
| EPIC | T cell CD8+ | -0.15 | 0.00 |
| EPIC | uncharacterized cell | 0.29 | 0.00 |
| MCPCOUNTER | B cell | -0.40 | 0.00 |
| MCPCOUNTER | cytotoxicity score | -0.16 | 0.00 |
| MCPCOUNTER | Endothelial cell | -0.27 | 0.00 |
| MCPCOUNTER | Macrophage/Monocyte | -0.16 | 0.00 |
| MCPCOUNTER | Monocyte | -0.16 | 0.00 |
| MCPCOUNTER | Myeloid dendritic cell | -0.36 | 0.00 |
| MCPCOUNTER | Neutrophil | -0.13 | 0.00 |
| MCPCOUNTER | NK cell | -0.17 | 0.00 |
| MCPCOUNTER | T cell | -0.39 | 0.00 |
| MCPCOUNTER | T cell CD8+ | -0.25 | 0.00 |
| QUANTISEQ | B cell | -0.30 | 0.00 |
| QUANTISEQ | Macrophage M1 | -0.15 | 0.00 |
| QUANTISEQ | Macrophage M2 | -0.47 | 0.00 |
| QUANTISEQ | Myeloid dendritic cell | 0.10 | 0.04 |
| QUANTISEQ | NK cell | -0.16 | 0.00 |
| QUANTISEQ | T cell CD4+ (non-regulatory) | 0.11 | 0.01 |
| QUANTISEQ | T cell CD8+ | -0.28 | 0.00 |
| QUANTISEQ | T cell regulatory (Tregs) | -0.39 | 0.00 |
| QUANTISEQ | uncharacterized cell | 0.35 | 0.00 |
| TIMER | B cell | -0.38 | 0.00 |
| TIMER | Macrophage | -0.21 | 0.00 |
| TIMER | Myeloid dendritic cell | -0.31 | 0.00 |
| TIMER | Neutrophil | -0.25 | 0.00 |
| TIMER | T cell CD4+ | -0.33 | 0.00 |
| TIMER | T cell CD8+ | -0.26 | 0.00 |
| XCELL | B cell | -0.34 | 0.00 |
| XCELL | B cell memory | -0.23 | 0.00 |
| XCELL | B cell naive | -0.11 | 0.02 |
| XCELL | B cell plasma | -0.19 | 0.00 |
| XCELL | Cancer associated fibroblast | -0.35 | 0.00 |
| XCELL | Class-switched memory B cell | -0.31 | 0.00 |
| XCELL | Common lymphoid progenitor | 0.34 | 0.00 |
| XCELL | Common myeloid progenitor | -0.11 | 0.02 |
| XCELL | Endothelial cell | -0.24 | 0.00 |
| XCELL | Granulocyte-monocyte progenitor | -0.26 | 0.00 |
| XCELL | Hematopoietic stem cell | -0.39 | 0.00 |
| XCELL | immune score | -0.46 | 0.00 |
| XCELL | Macrophage | -0.30 | 0.00 |
| XCELL | Macrophage M1 | -0.20 | 0.00 |
| XCELL | Macrophage M2 | -0.29 | 0.00 |
| XCELL | Mast cell | -0.33 | 0.00 |
| XCELL | microenvironment score | -0.50 | 0.00 |
| XCELL | Monocyte | -0.30 | 0.00 |
| XCELL | Myeloid dendritic cell | -0.38 | 0.00 |
| XCELL | Myeloid dendritic cell activated | -0.37 | 0.00 |
| XCELL | Plasmacytoid dendritic cell | -0.14 | 0.00 |
| XCELL | stroma score | -0.35 | 0.00 |
| XCELL | T cell CD4+ (non-regulatory) | -0.11 | 0.02 |
| XCELL | T cell CD4+ central memory | -0.23 | 0.00 |
| XCELL | T cell CD4+ effector memory | -0.24 | 0.00 |
| XCELL | T cell CD4+ naive | -0.37 | 0.00 |
| XCELL | T cell CD4+ Th1 | 0.21 | 0.00 |
| XCELL | T cell CD4+ Th2 | 0.28 | 0.00 |
| XCELL | T cell CD8+ | -0.37 | 0.00 |
| XCELL | T cell CD8+ central memory | -0.32 | 0.00 |
| XCELL | T cell NK | -0.22 | 0.00 |
| XCELL | T cell regulatory (Tregs) | -0.11 | 0.01 |
